# Towards Selective Mycobacterial ClpP1P2 Inhibitors with Reduced Activity against the Human Proteasome

**DOI:** 10.1128/AAC.02307-16

**Published:** 2017-04-24

**Authors:** Wilfried Moreira, Sridhar Santhanakrishnan, Grace J. Y. Ngan, Choon Bing Low, Kanda Sangthongpitag, Anders Poulsen, Brian W. Dymock, Thomas Dick

**Affiliations:** a Department of Microbiology and Immunology, Yong Loo Lin School of Medicine, National University of Singapore, Singapore; b Department of Pharmacy, National University of Singapore, Singapore; c Experimental Therapeutics Center, Agency for Science, Technology and Research (A*STAR), Singapore; d Department of Chemistry, National University of Singapore, Singapore

**Keywords:** caseinolytic protease, antimycobacterial, dipeptidyl boronic acid

## Abstract

Mycobacterium tuberculosis is responsible for the greatest number of deaths worldwide due to a bacterial agent. We recently identified bortezomib (Velcade; compound 1) as a promising antituberculosis (anti-TB) compound. We showed that compound 1 inhibits the mycobacterial caseinolytic proteases P1 and P2 (ClpP1P2) and exhibits bactericidal activity, and we established compound 1 and ClpP1P2 as an attractive lead/target couple. However, compound 1 is a human-proteasome inhibitor currently approved for cancer therapy and, as such, exhibits significant toxicity. Selective inhibition of the bacterial protease over the human proteasome is desirable in order to maintain antibacterial activity while reducing toxicity. We made use of structural data in order to design a series of dipeptidyl-boronate derivatives of compound 1. We tested these derivatives for whole-cell ClpP1P2 and human-proteasome inhibition as well as bacterial-growth inhibition and identified compounds that were up to 100-fold-less active against the human proteasome but that retained ClpP1P2 and mycobacterial-growth inhibition as well as bactericidal potency. The lead compound, compound 58, had low micromolar ClpP1P2 and anti-M. tuberculosis activity, good aqueous solubility, no cytochrome P450 liabilities, moderate plasma protein binding, and low toxicity in two human liver cell lines, and despite high clearance in microsomes, this compound was only moderately cleared when administered intravenously or orally to mice. Higher-dose oral pharmacokinetics indicated good dose linearity. Furthermore, compound 58 was inhibitory to only 11% of a panel of 62 proteases. Our work suggests that selectivity over the human proteasome can be achieved with a drug-like template while retaining potency against ClpP1P2 and, crucially, anti-M. tuberculosis activity.

## INTRODUCTION

Mycobacterium tuberculosis, the causative agent of tuberculosis (TB), remains the biggest bacterial killer throughout history. In 2015 alone, there were 10.4 million recorded cases of TB infection, with an estimated 1.8 million deaths, including 0.4 million deaths due to TB disease in HIV-infected people. There were 580,000 new cases of multidrug-resistant patients in 2015, further compounding the situation (1). There is an urgent medical need for new drugs with new mechanisms of action to control drug-resistant disease (2–4).

**FIG 1:**
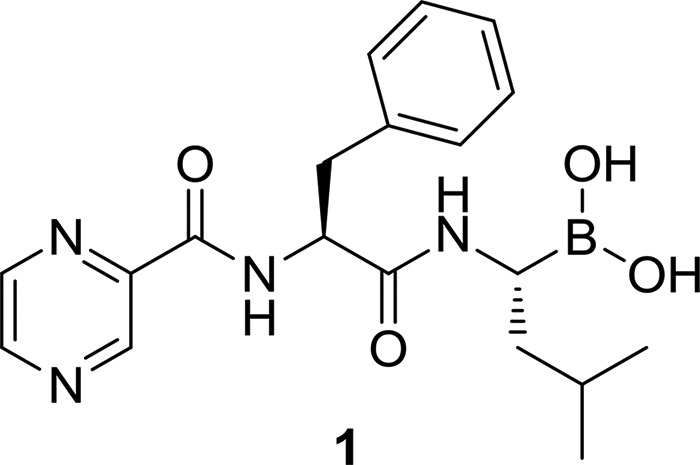
Structure of bortezomib (compound 1).

Caseinolytic proteases P1 and P2 (ClpP1P2) are serine proteases found in a wide range of bacteria (5–7). In contrast to site-specific proteases, caseinolytic proteases form a degradative complex involved in the removal of partially synthesized and misfolded proteins. The caseinolytic protease complex is composed of catalytic subunits (ClpP) and regulatory subunits (ATPases). The regulatory subunits recognize substrates and provide the energy for unfolding proteins that are to be degraded. The catalytic ClpP subunits form a degradative chamber in which proteolysis occurs. The proteolytic chamber of mycobacterial caseinolytic protease consists of two different subunits, ClpP1 and ClpP2 (8–10) Caseinolytic proteases are involved in the removal of incomplete translation products. The transfer-messenger RNA (tmRNA) *trans*-translation rescue system frees ribosomes by tagging aborted proteins with a caseinolytic-protease-specific degradation peptide (SsrA), identifying the partially synthesized product for Clp-specific degradation. We made use of this mechanism and employed this ClpP1P2-specific degradation tag to develop a fluorescence-based synthetic phenotype to detect and measure intracellular ClpP1P2 inhibition. Using this approach, we identified compound 1 as the first mycobacterial caseinolytic protease inhibitor with whole-cell bactericidal activity and as a promising lead candidate against TB (11). Compound 1 is an N-substituted dipeptidyl-boronate with a CAP-Phe-Leu-boronate sequence (Fig. 1). We modeled compound 1 into the ClpP1 and ClpP2 catalytic sites and showed good site complementarity following covalent-bond formation between the boronic acid warhead and the serine of the catalytic triad (Ser98 and Ser110 in ClpP1 and ClpP2, respectively). The hydrophobic side chain of compound 1 is also consistent with the protease P1 site preference for a hydrophobic residue (9, 12).

Boronic acid 1 is the first proteasome inhibitor approved by the U.S. FDA for the treatment of multiple myeloma and mantle cell lymphoma (13–15). Like the bacterial caseinolytic protease, the human proteasome is a degradative protease complex involved in proteome housekeeping. The crystal structure of the yeast proteasome in complex with compound 1 has been resolved (16). A covalent adduct between the boronic acid warhead of compound 1 and the catalytic hydroxyl group of threonine forms in the active site of the proteasome. This strong blockade leads to enzyme dysfunction, cell cycle arrest, and apoptosis in cancer cells (16, 17). However, compound 1 is given intravenously (i.v.), an unfavorable route of administration that is compounded by poor pharmacokinetics, high costs, and significant adverse events, including peripheral neuropathy, neutropenia, and cytopenia (18, 19), all factors which limit its direct use for tuberculosis. Despite these negatives, compound 1 has been used to successfully treat many cancer patients, and boronic acid prodrugs have led to orally available compounds (20). It was against this background that we set out to explore how we could modify the structure of compound 1 to reduce its strong proteasome inhibition while maintaining, or improving, its activity against ClpP1P2. Structural differences between the human proteasome and the mycobacterial caseinolytic protease led us to believe that optimization of compound 1 was an attractive opportunity (9, 16, 21). We synthesized and tested a series of dipeptidyl boronate derivatives of compound 1, with variation at the P1, P2, and CAP side chains. We report here the identification of new compounds 100-fold-less active against the human proteasome but which retain low-micromolar potency against ClpP1P2 and inhibit bacterial growth. Before embarking on a medicinal-chemistry program, we required suitable assays to measure the effects of new compounds. An important consideration was the ability to track ClpP1P2 activity and bacterial structure-activity relationships (SAR) without interference from mycobacterial proteasome inhibition. We therefore developed a target-based cell reporter assay for on-target potency determination. This assay is against a cellular background; hence, it requires compounds to penetrate bacteria, a key challenge in antibacterial drug discovery.

## RESULTS AND DISCUSSION

### Assay development. (i) Genetic engineering of M. smegmatis proteasome null mutant strain.

**FIG 2:**
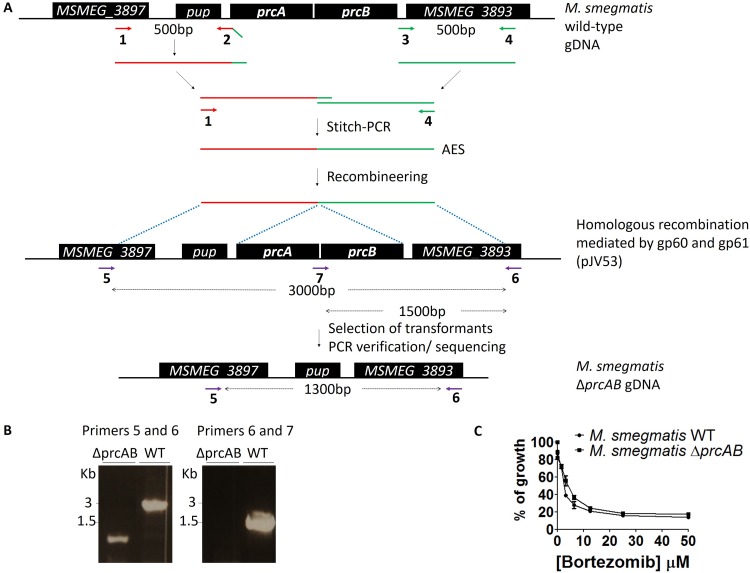
Genetic engineering of M. smegmatis *prcAB* null mutant strain by recombineering. (A) Schematic representation of the recombineering strategy used to delete *prcAB* genes in M. smegmatis. (B) PCR verification of the M. smegmatis *prcAB* null mutant. The primers used to generate the AES and to verify the null mutant are described in Table S1 in the supplemental material. (C) Compound 1 growth inhibition of the M. smegmatis wild-type and *prcAB* null mutant strains. WT, wild type.

We previously identified compound 1, a human-proteasome inhibitor, as a whole-cell-active ClpP1P2 protease inhibitor in mycobacteria (11). Compound 1 was first identified using Mycobacterium smegmatis as a nonpathogenic screening strain in which the proteasome is known to be nonessential (22). The activity of compound 1 against M. tuberculosis, in which the proteasome, while remaining nonessential, has been shown to be critical for virulence and survival, was subsequently confirmed (23). In order to exclude any proteasome cross-reactivity of the derivatives of compound 1 that we wished to test, we sought to delete the genes coding for the two proteasome subunits, *prcA* and *prcB*, in M. smegmatis. We employed a recombineering approach and selectively targeted and deleted these two genes to generate an *M. smegmatis prcAB* null mutant strain (Fig. 2). An allelic-exchange substrate (AES) with homology to the flanking regions of the *prcAB* locus was constructed using stitch-PCR (Fig. 2A) and electroporated into a strain of M. smegmatis previously transformed with the plasmid pJV53. pJV53 carries the genes encoding the recombinase gp60 and the resolvase gp61, which mediate the homologous recombination of the AES. Upon recombination and gene deletion, we verified the recombinant null mutant strain using discriminatory PCR (Fig. 2B). As expected, PCR amplification using primers 5 and 6 flanking the *prcAB* locus gave rise to amplicons of 1.3 kb in the recombinant *prcAB* null mutant, compared to amplicons of 3 kb in the wild-type strain. Similarly, PCR amplification using primers 6 and 7 generated amplicons visible only in the wild-type strain. Finally, we confirmed that the susceptibility of compound 1 was not affected by the *prcAB* deletion (Fig. 2C). Both the wild-type and the null mutant strains showed similar levels of growth inhibition when exposed to increasing concentrations of compound 1 with the same MIC_50_ of 5 μM. The *M. smegmatis ΔprcAB* strain was subsequently transformed with a plasmid carrying an SsrA-tagged red fluorescent protein (RFP).

### ClpP1P2, proteasome, and bacterial-growth inhibition assays.

**FIG 3:**
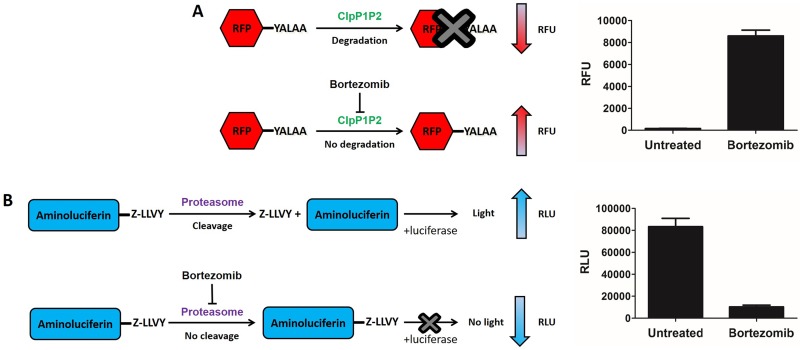
ClpP1P2 and proteasome inhibition assays. (A) ClpP1P2 inhibition assay principle. Without any interference, ClpP1P2 recognizes and degrades SsrA-tagged (YALAA) RFP protein, resulting in a low fluorescence level. In the presence of a ClpP1P2 inhibitor (compound 1, bortezomib), RFP is not degraded. Its accumulation results in an increase in fluorescence. (B) Proteasome inhibition assay principle. Without any interference, the proteasome recognizes the Z-LLVY tag and cleaves it. The aminoluciferin is used as a substrate by the luciferase enzyme to generate luminescence. In the presence of a proteasome inhibitor (compound 1), the cleavage of Z-LRR is prevented. The lack of the luciferase substrate results in a reduced luminescence emission. RFU, relative fluorescence units; RLU, relative luminescence units.

We employed two target-based whole-cell assays, the mycobacterial-ClpP1P2 and human-proteasome inhibition assays, in order to evaluate the selectivity of derivatives of compound 1 for the bacterial target. The ClpP1P2 inhibition assay measures the intracellular accumulation of RFP-SsrA as a result of ClpP1P2 inhibition (11). The principle of the assay is as follows. Under undisturbed conditions, ClpP1P2 recognizes and degrades the RFP-SsrA protein to a background level of fluorescence. An inhibitor of ClpP1P2, like compound 1, binds to the catalytic sites of the protease and prevents the degradation of the RFP-SsrA protein, resulting in its accumulation and a gain-of-fluorescence signal (Fig. 3A). Similarly, the human-proteasome inhibition assay makes use of a proteasome-specific cleavage tag (Z-LLVY) fused to an aminoluciferin molecule. Under undisturbed conditions, the chymotrypsin-like catalytic site of the proteasome recognizes the tag and cleaves it, releasing the aminoluciferin molecule. The free aminoluciferin is a substrate for the luciferase enzyme in a reaction that produces luminescence. In the presence of a proteasome inhibitor, like compound 1, the cleavage is blocked, preventing the release of the aminoluciferin and the subsequent emission of luminescence (Fig. 3B). In order to determine both the ClpP1P2 and proteasome 50% inhibitory concentrations (IC_50_s) for all derivatives of compound 1 (i.e., the concentration required to inhibit 50% of the ClpP1P2 or proteasome activity), we tested new compounds in a dose-response analysis in both assays, from which we determined the IC_50_s. We further evaluated the growth inhibition potency of each derivative using a standard turbidity-based growth inhibition assay. Exponentially growing cultures of *M. smegmatis ΔprcAB* were exposed to increasing concentrations of a given derivative. After 24 h of exposure, culture turbidity (i.e., growth) was assessed by measuring optical density at 600 nm (OD_600_) and plotted as a function of compound concentration. MIC_50_s were then determined (i.e., the concentration required to inhibit 50% of growth compared to the growth of an untreated control). In all assays, compound 1 was used as a positive control and a reference compound.

From these three whole-cell biological assays, we could evaluate the activities of our derivatives both against the bacterial target, ClpP1P2 (ClpP1P2 IC_50_), and against bacterial growth itself (MIC_50_) as well as against the human proteasome (proteasome IC_50_). Measuring both ClpP1P2 and bacterial-growth dose responses ensured that new compounds remained on target and were correlated with desired phenotypic disease activity. Using cell-based assays for the assessment of new compounds ensures that bacterial penetration is built in and, hence, increases the drug-like character of new compounds.

### Design of compounds.

**FIG 4:**
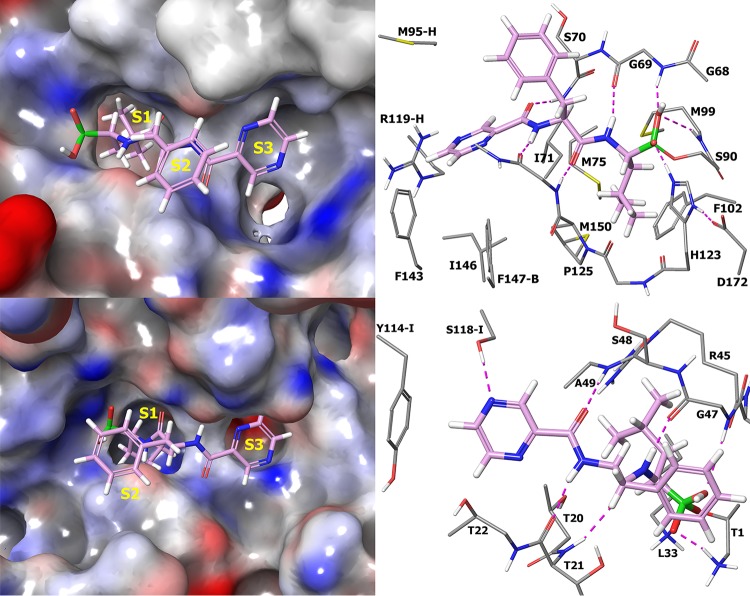
Docking of compound 1 into M. tuberculosis ClpP1P2 (top) (PDB accession number 4U0G) and the human proteasome (bottom) (PDB accession number 4R67). Compound 1 is shown as a thick tube with a plum carbon. (Left) Molecular surface of M. tuberculosis ClpP1P2 and the human proteasome in gray, blue, and red, indicating neutral, positive, and negative electrostatics, respectively, with substrate sites 1 to 3 (S1 to S3) labeled in yellow. There is more available space in the S1 and S3 sites of ClpP1P2 than in the human proteasome. (Right) Selected residues of M. tuberculosis ClpP1P2 and the human proteasome are shown as a thin tube, with gray carbon and hydrogen bonds as dashed magenta lines. Residues are from the same protein subunit unless marked by a suffix indicating the PDB chain. The hydrogen bond network between compound 1 and the human proteasome is retained when compound 1 binds to M. tuberculosis ClpP1P2, and consequently, we did not try to modify the backbone of compound 1. The orientation of the P2 side chain differs, but there is little interaction between this and either protein, and modeling indicates that it is free to move around in the binding site.

At the outset of this project, our primary goal was to identify compounds which inhibit bacterial ClpP1P2 in a bacterial cell but which have reduced potency against the human proteasome compared to that of compound 1. Modeling studies suggested that a larger P1 substituent could be tolerated by the ClpP1P2 P1 pocket but should be less well accommodated in the P1 pocket of the human proteasome (Fig. 4). We therefore prioritized exploration of both aromatic and saturated rings directly attached to the P1 backbone carbon of the inhibitor. Given the uncertainty in the precise nature of the P1 group, we also planned to prepare a range of larger substituents connected at P1 via progressively longer linkers. For initial studies, we planned only minimal variations of the P2 phenylalanine side chain but wider variations of the CAP group. Our rationale was that CAP group changes would be well tolerated in this area of the molecule, and given the significant role that bacterial cell penetration is likely to play, the CAP group represents a good opportunity for tuning of physicochemical properties and hence of ClpP1P2 and antibacterial activity.

### Chemistry.

**FIG 5:**
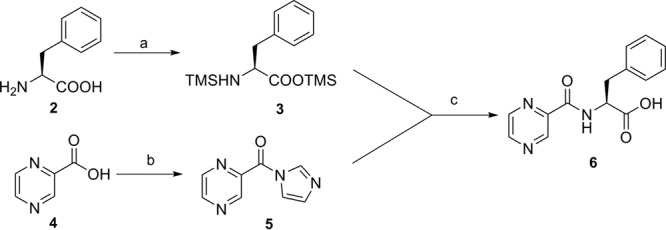
Synthesis of pyrazine acid 6. Reagents and conditions: (a) BSA, DCM, rt, 16 h; (b) CDI, DCM, rt, 16 h; (c) −40°C to rt, 16 h.

Preparation of final compounds with the P2 substituent fixed as phenylalanine (benzyl side chain) and the CAP group fixed as pyrazine was accomplished via acid intermediate 6, prepared from l-phenylalanine (compound 2) and 2-pyrazinecarboxylic acid (compound 4) (Fig. 5). Silylation of compound 2 was achieved with *N*,*O*-bis(trimethylsilyl)-acetamide (BSA), furnishing compound 3. Subsequently, acylation of compound 3 with pyrazine imidazolide 5, prepared from the coupling of compound 4 with imidazole, gave compound 6 in overall good yield (24).

**FIG 6:**
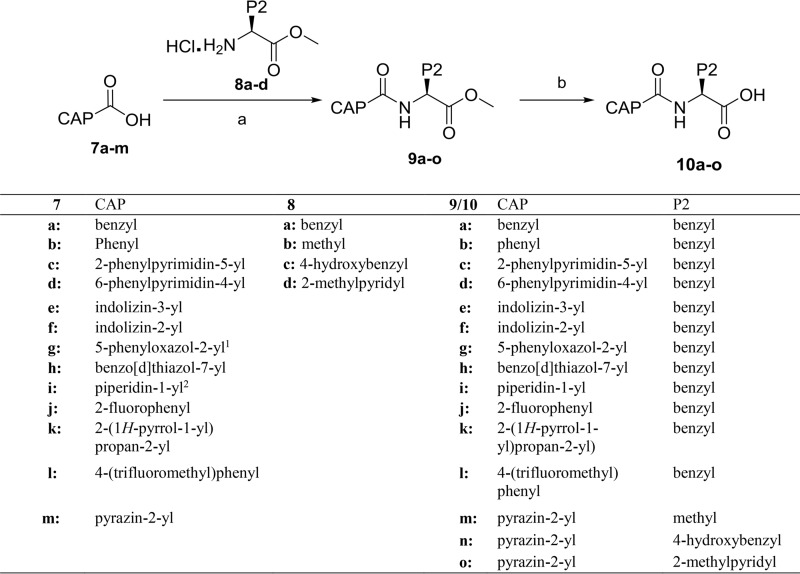
General synthesis of carboxylic acid derivatives 10a to -o. Reagents and conditions: (a) 8a-d, TBTU, DIPEA, CH_2_Cl_2_, 0°C to rt; (b) LiOH, THF:MeOH:H_2_O, 0°C to rt; ^1^lithium salt of 5-phenyloxazole-2-carboxylic acid; ^2^piperidine-1-carbonyl chloride.

Alternatively, acid intermediates 10a to -o, with variations in both P2 and CAP groups, were prepared using standard peptide synthesis procedures (Fig. 6). Esters 9a to -o were obtained by coupling of carboxylic acids 7a to -m and amines 8a to -d in the presence of 2-(1*H*-benzotriazole-1-yl)-1,1,3,3-tetramethyluronium tetrafluoroborate (TBTU). Subsequently, esters 9a to -o were hydrolyzed under basic conditions, furnishing carboxylic acids 10a to -o.

**FIG 7:**
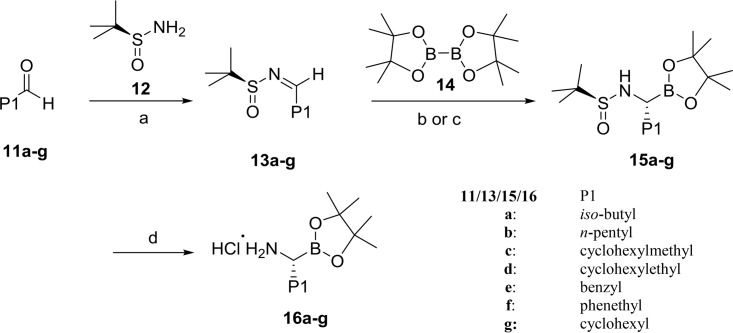
General synthesis of amino boronate salts 16a to -g. Reagents and conditions: (a) CuSO_4_·5H_2_O, 4 Å mol. sieves, CH_2_Cl_2_, rt; (b) CuSO_4_, BnNH_2_, PCy_3_·HBF_4_, 5:1 toluene/H_2_O, rt; (c) (ICy)CuOt-Bu, dry toluene, rt; (d) 4.0 M HCl in dioxane, dry MeOH, dry dioxane, rt.

Key P1 amino boronate salts 16a to -g were prepared using Ellman's protocol (Fig. 7) (25). *N*-Sulfinimines 13a to -g were synthesized by condensation between *tert*-butylsulfinamide (compound 12) and aldehydes 11a to -g in the presence of copper sulfate and molecular sieves. Boronate esters 15a to -f were obtained in high diastereoselectivity from the borylation of compounds 13a to -f with bis(pinacolato)diboron (compound 14) in the presence of copper sulfate. Alternatively, cyclohexyl boronate ester 15g was obtained from compound 13g in a yield of 33% by employing (ICy)CuO*t*-Bu as a catalyst (26). In order to obtain an acceptable yield, the catalyst (ICy)CuO*t*-Bu should be prepared freshly from (ICy)CuCl (27). Selective removal of the *N*-sulfinyl group under acidic conditions afforded amine hydrochlorides 16a to -g.

**FIG 8:**
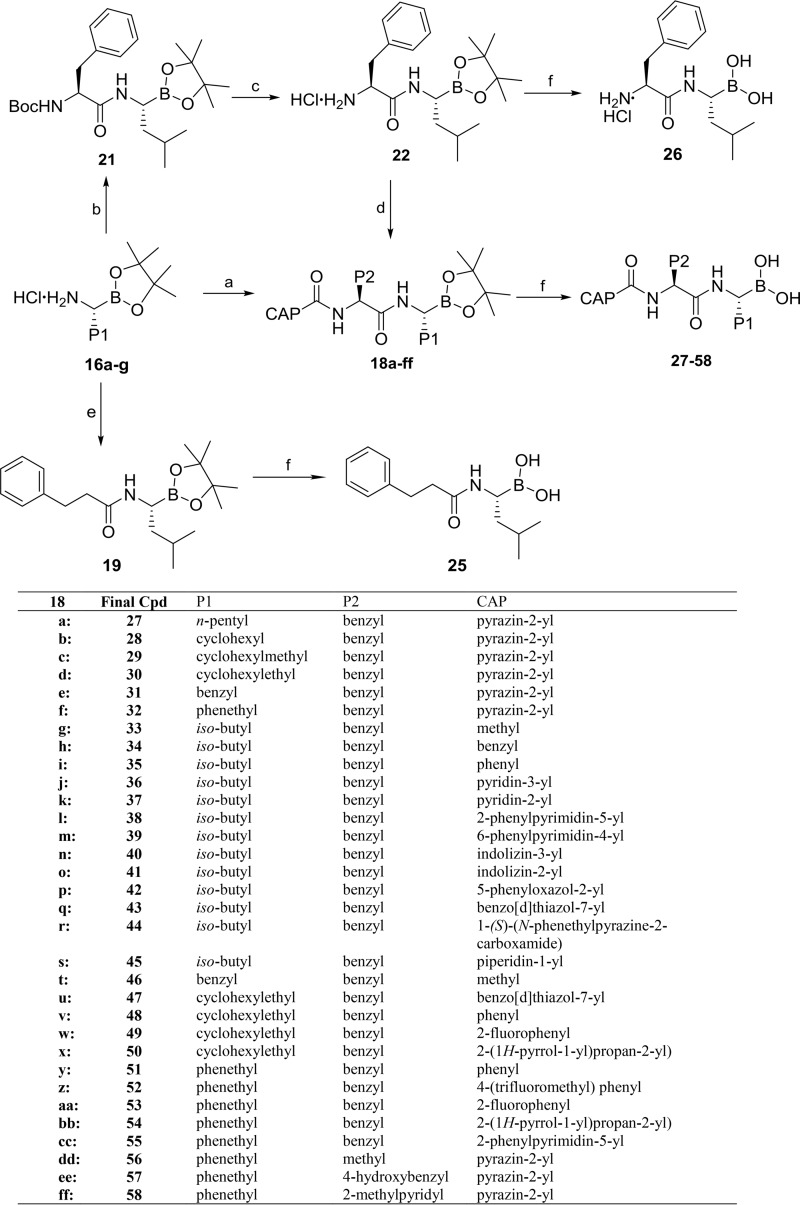
General synthesis of boronic acids 25 to 58. Reagents and conditions: (a) 6/10a-o,TBTU, *i*Pr_2_EtN, CH_2_Cl_2_, 0°C to rt; (b) 20, TBTU, *i*Pr2EtN, dry CH_2_Cl_2_, 0°C to rt; (c) 4.0 M HCl in dioxane, CH_2_Cl_2_, rt; (d) 6/7b/23/24, TBTU, *i*Pr_2_EtN, dry CH_2_Cl_2_, 0°C to rt; (e) 17, TBTU, *i*Pr_2_EtN, CH_2_Cl_2_, 0°C to rt; (f) *i*BuB(OH)_2_, 1 N HCl, CH_3_OH, pentane, rt.

Synthesis of penultimate diamide boronic esters 19, 22, and 18a to -ff was achieved by employing a linear or convergent approach (Fig. 8). TBTU facilitated coupling between amine salts 16a to -g with 3-phenylpropanoic acid (compound 17), and acid intermediates 6/10a to -o gave boronic esters compound 19 and 18a to -ff, respectively. Alternatively, in a linear approach (done only for P2 [= Phe]), compound 16a was coupled with *N*-butoxycarbonyl-l-phenylalanine (*N*-Boc-l-phenylalanine) (compound 20), furnishing *N*-Boc amide 21. Subsequently, the Boc group was cleaved under acidic conditions to give amine 22, isolated as a hydrochloride salt. Acids 6, benzoic acid (compound 7b), nicotinic acid (compound 23), and picolinic acid (compound 24) were coupled with compound 22 to provide 18a and 18i to -k. Finally, under acidic conditions, boronic esters compounds 19, 22, and 18a to -ff were transesterified with *iso*-butyl boronic acid to provide the target boronic acids 25 to 58.

### Structure-activity relationships.

In order to understand the roles of the P1, P2, and CAP groups of the boronic acids with regard to ClpP1P2 targeting, antimycobacterial-growth inhibition, and selectivity against the human proteasome, various substituents on the P1, P2, and CAP positions were studied. In these assays, compound 1 has an IC_50_ of 6 μM in the ClpP1P2 cell reporter assay, which translates to the same concentration as its MIC_50_ for the growth inhibition of *M. smegmatis ΔprcAB*, whereas potency for the human proteasome was in the single-digit nanomolar range (IC_50_ = 5 nM) (Table 1). This profile translates to a selectivity preference for the human proteasome of 1,200-fold, not surprising given the fact that compound 1 is a highly optimized proteasome inhibitor. Hence, the objective of this work was to prepare new compounds with a reduced preference for the human proteasome while maintaining or improving the potency against ClpP1P2. Guided by modeling, the influence of substituents at the P1 position was initially studied.

**TABLE 1:**
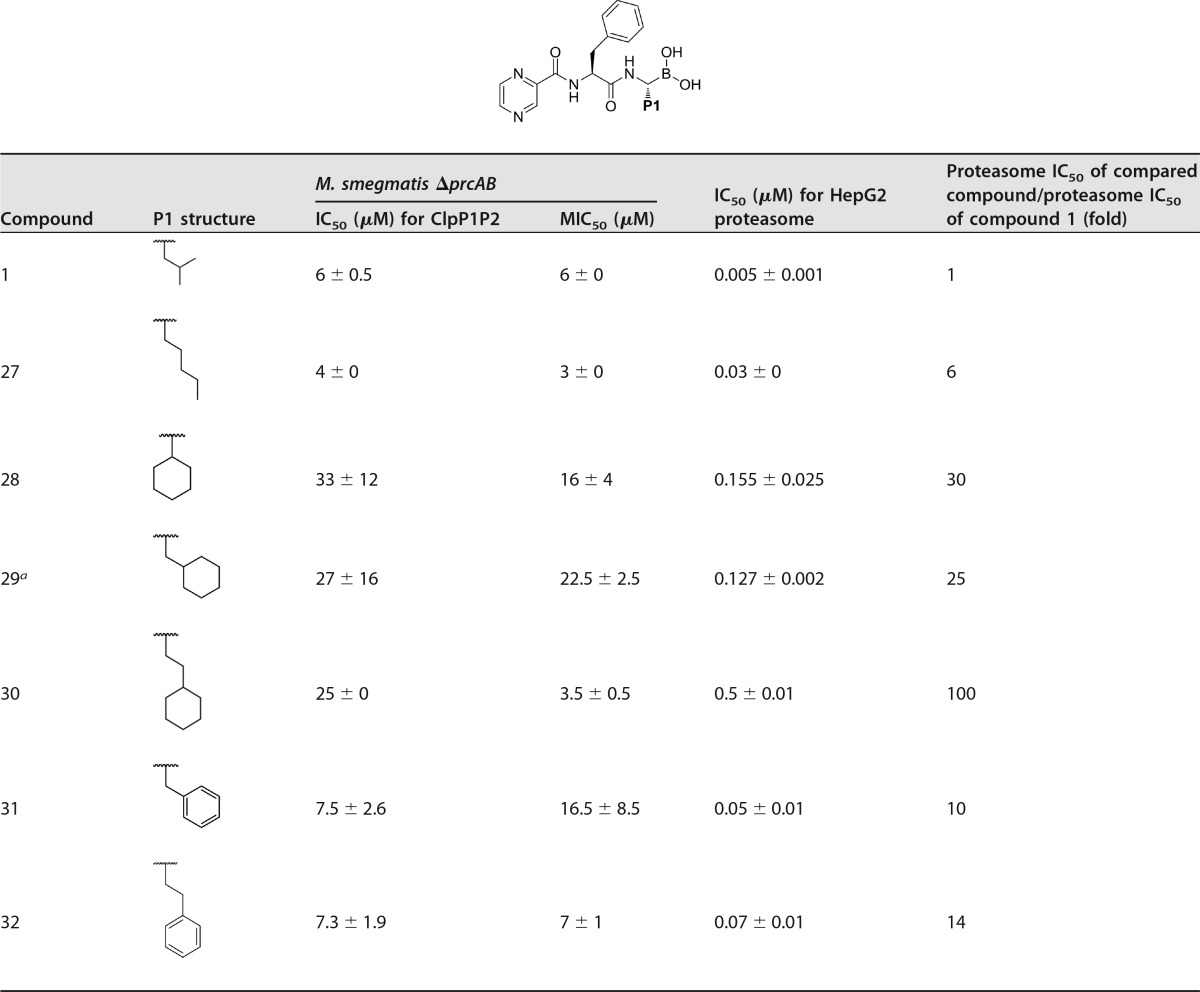
Derivatives of compound 1 with P1 modifications

^a^ dr ratio, 3.5:1 (dr mixture at the P1 center).

Replacing the *iso*-butyl in compound 1 with a less hindered straight-chain *n*-pentyl (compound 27) resulted in a 2-fold improvement in antibacterial activity, while ClpP1P2 potency was maintained at 4 μM. Encouragingly, this compound was 6-fold-less potent than compound 1 in the proteasome assay (see the proteasome potency ratio in Table 1), with an IC_50_ of 30 nM. Increasing the steric bulk of P1 with a cyclohexyl group directly attached (compound 28) further reduced proteasome activity to 155 nM (30-fold less active than compound 1). It appeared that our strategy of increasing the size of the P1 group did influence selectivity. However, disappointingly, the potency against ClpP1P2 of compound 28 was reduced to only 33 μM, with corresponding reduced antibacterial activity.

Inserting a methylene linker to position the cyclohexyl further from the inhibitor backbone (compound 29) produced a similar result, but adding one additional methylene (cyclohexylethyl 30) further reduced proteasome activity to 0.5 μM, an overall 100-fold reduction compared to the activity of compound 1. Unfortunately a significant drop in the potency against ClpP1P2 was also observed. Moreover, this compound still exhibited bacterial-growth inhibition (MIC_50_ = 3.5 μM), suggesting that this activity was not due to ClpP1P2 inhibition. We were also concerned about increasing hydrophobicity and poor aqueous solubility with lipophilic cyclohexyl derivatives. Therefore, we next tested aromatic P1 groups. However, only 10-fold-less proteasome activity over that of compound 1 was achieved with benzyl P1-substituted compound 31, and growth-inhibitory potency was also reduced to 16.5 μM. Potency against ClpP1P2 and the growth MIC_50_ were maintained with a phenethyl P1 group compound (compound 32) which had a 14-fold-lower potency for the proteasome (IC_50_ = 70 nM) than compound 1.

Various groups at the CAP position were next studied to explore their influence on the ClpP1P2 and proteasome potency (Table 2). Removal of the entire CAP group and the P2 amine (compound 25) abolished both the bacterial-growth inhibition and the ClpP1P2 activity. Replacing only the P2 amine, without CAP, gave compound 26, with similar results. These non-CAP compounds still retained proteasome activity, demonstrating the considerable challenge of reducing activity against the proteasome in this series while retaining ClpP1P2 potency.

**TABLE 2:**
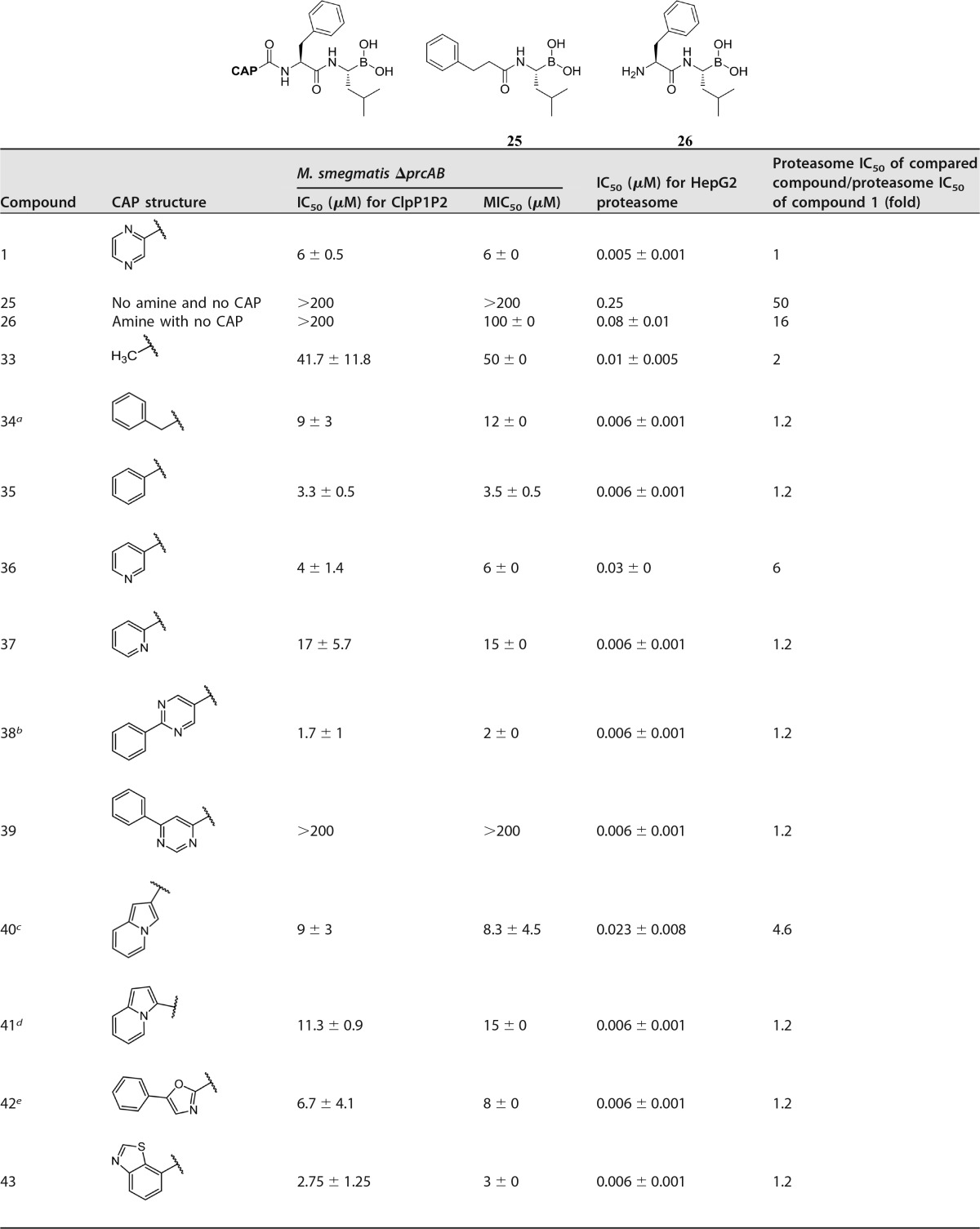

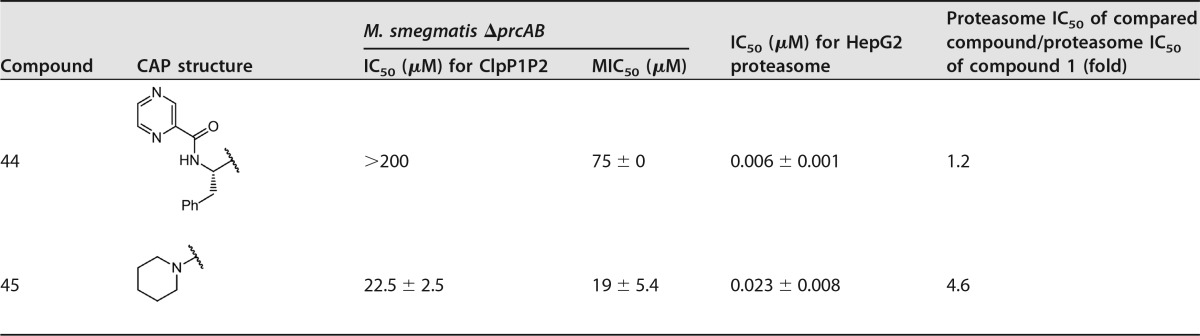
Derivatives of compound 1 with CAP modifications

^a^ dr ratio, 7.3:1 (dr mixture at the P2 center).

^b^ dr ratio, 6:1 (dr mixture at the P2 center).

^c^ dr ratio, 3.4:1 (dr mixture at the P2 center).

^d^ dr ratio, 4.4:1 (dr mixture at the P2 center).

^e^ dr ratio, 6:1 (dr mixture at the P2 center).

Upon reducing the size of the CAP group to methyl (compound 33), ClpP1P2 activity partially returned (42 μM), indicating that a CAP group is strictly required for minimal ClpP1P2, as well as antibacterial activity. Potency was further regained with benzyl (compound 34), suggesting that a bulky CAP is required for ClpP1P2 potency. However, no reduction in proteasome activity at all was achieved with this compound. An increase of 2-fold ClpP1P2 activity was obtained with phenyl analogue 35, compared to the ClpP1P2 activity of pyrazine compound 1. Unfortunately proteasome activity was unchanged.

With a 3-pyridyl CAP group (compound 36) the proteasome activity was reduced by 6-fold, with retention of ClpP1P2 activity, while with a 2-pyridyl (compound 37), both ClpP1P2 potency and proteasome potency were reduced. These data indicate that the specific position of the nitrogen in the aromatic CAP is important for ClpP1P2 activity and may have a role to play in proteasome activity as well. More-bulky heterocyclic groups were also screened, with significant SAR findings. 5-Substituted 2-phenylpyrimidine (compound 38) maintained activities against ClpP1P2 and bacterial growth; however, in contrast, the isomer 4-substituted 6-phenylpyrimidine (compound 39) completely lost potency in the target assays but fully retained proteasome potency. We speculated that this might be due to either poor bacterial-cell penetration or poor target binding due to repulsive interactions in the ClpP1P2 active site. However, modeling studies revealed that this compound binds the ClpP1P2 active site with no apparent repulsive interactions. We therefore concluded that poor bacterial-cell penetration (with maintenance of mammalian-cell penetration) might be the reason for the poor ClpP1P2 and antibacterial potency. 3-Indolizine (compound 40) retained target potency but was only 5-fold-less active for the proteasome than compound 1. However, 2-indolizine (compound 41) was slightly less active against ClpP1P2 and more potent against the proteasome and hence did not offer clues as to a way forward. No change in selectivity or potency was observed with 2-substituted 5-oxazole (compound 42). This compound has a substitution pattern similar to that of compound 39, which was not active. Benzothiazole (compound 43) was quite potent, with a 2-fold increase in ClpP1P2 and bacterial-growth inhibition compared with that of compound 1. Inserting a P2 phenylalanine and maintaining the pyrazine CAP (“extended pyrazine,” compound 44) led to complete loss of ClpP1P2 potency and no improvement in proteasome activity. However, 5-fold-reduced proteasome activity was achieved with piperidine urea 45, but with reduced ClpP1P2 and bacterial-growth inhibition. This series of CAP changes does not provided a clear way forward but does offer options of CAP groups for subsequent P1-P2-CAP combinations for the next phase of SAR exploration.

To obtain increased ClpP1P2 potency and reduced activity against the proteasome, combinations of the best-performing P1 groups, such as benzyl, cyclohexylethyl, and phenethyl, and selected CAP groups were next screened (Table 3).

**TABLE 3:**
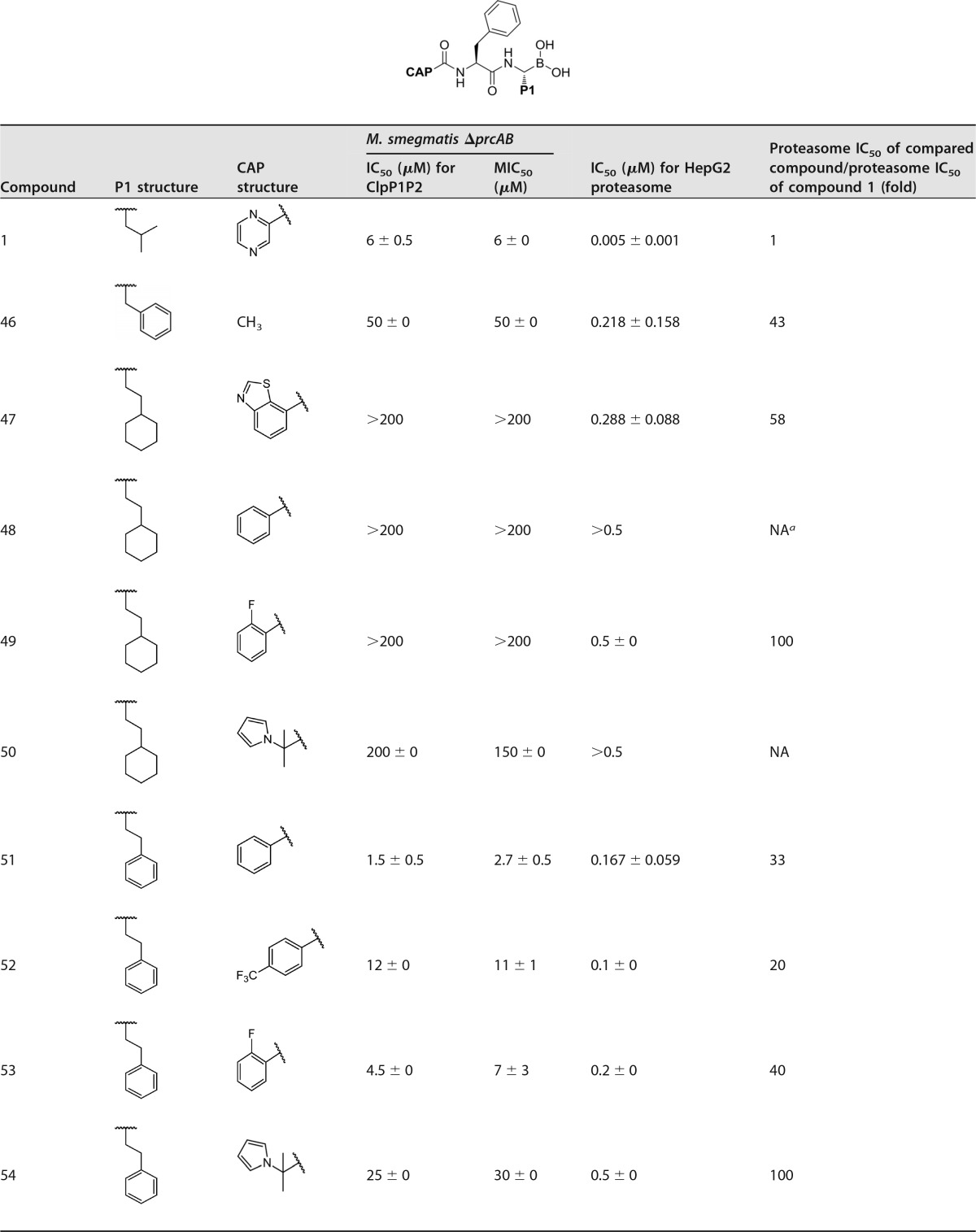

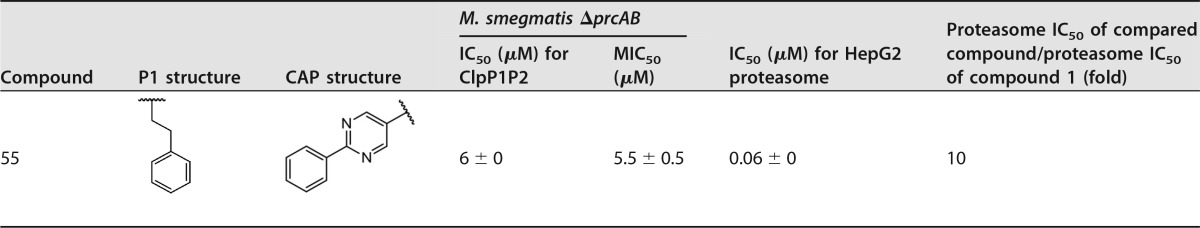
Derivatives of compound 1 with P1 and CAP modifications

^a^ NA, not active in the primary assay.

Encouragingly, a 43-fold reduction in proteasome potency was obtained when benzyl P1 and methyl CAP were combined (compound 46). However, poor target potency was achieved with this compound. This confirmed our previous observation that a bulky CAP is strictly required for ClpP1P2 potency. Disappointingly, no activity was detectable with the combination of cyclohexylethyl with ClpP1 and CAP groups, such as benzothiazole (compound 47), phenyl (compound 48), 2-fluorophenyl (compound 49), and a hindered pyrrole analogue (compound 50). We suspected that the LogP (the partition coefficient of a molecule between an aqueous and a lipophilic phase) of these compounds was too high, reducing aqueous solubility. Interestingly, with phenethyl as ClpP1 and phenyl as CAP (compound 51), proteasome activity was reduced by 33-fold compared to that of compound 1 (0.005 μM), with an IC_50_ of 0.167 μM. Furthermore, this compound is also slightly more active against ClpP1P2 and it has greater bacterial-growth inhibition. However, reduced antibacterial activity was observed with 4-(trifluoromethyl) phenyl (compound 52) and 2-fluorophenyl (compound 53), albeit with reduced proteasome activity. Pyrrole analogue (compound 54) exhibited 100-fold-reduced potency against the proteasome but unfortunately was only weakly potent against ClpP1P2 and bacterial growth. A larger CAP group, 5-substituted 2-phenylpyrimidine (compound 55), was 10-fold-less active than compound 1 against the proteasome but maintained ClpP1P2 and bacterial-growth inhibition. With this study of ClpP1-CAP combinations, we obtained compounds whose potencies against the target were retained but whose activities against the proteasome were reduced. We then considered changes at the P2 side chain in efforts to further optimize the series.

P1/P2 dual modifications were screened with pyrazine as the CAP group (Table 4). When the P2 side chain was reduced to a methyl (compound 56), the proteasome activity decreased 45-fold to 0.222 μM; however, ClpP1P2 inhibition and antibacterial activity significantly dropped as well. As with the CAP SAR from Table 2, this result indicates that a bulky P2 group is required for ClpP1P2 activity and bacterial-growth inhibition. Increasing the hydrophilicity at the P2 position of compound 32 with tyrosine in place of phenylalanine, giving compound 57, resulted in reduced proteasome activity (107-fold-less active than compound 1) and reduced retention of target activity compared to those of compound 1. Introducing a nitrogen into the phenyl ring to give a 2-pyridyl phenylalanine derivative (compound 58) improved potency against ClpP1P2 4-fold, and bacterial-growth inhibition improved just over 2-fold compared to that of compound 1. With 74-fold-reduced proteasome activity (IC_50_ = 0.367 μM) compared to that of compound 1, compound 58 demonstrates that it is possible to retain anti-ClpP1P2 and antibacterial activity while reducing potency against the human proteasome.

**TABLE 4:**
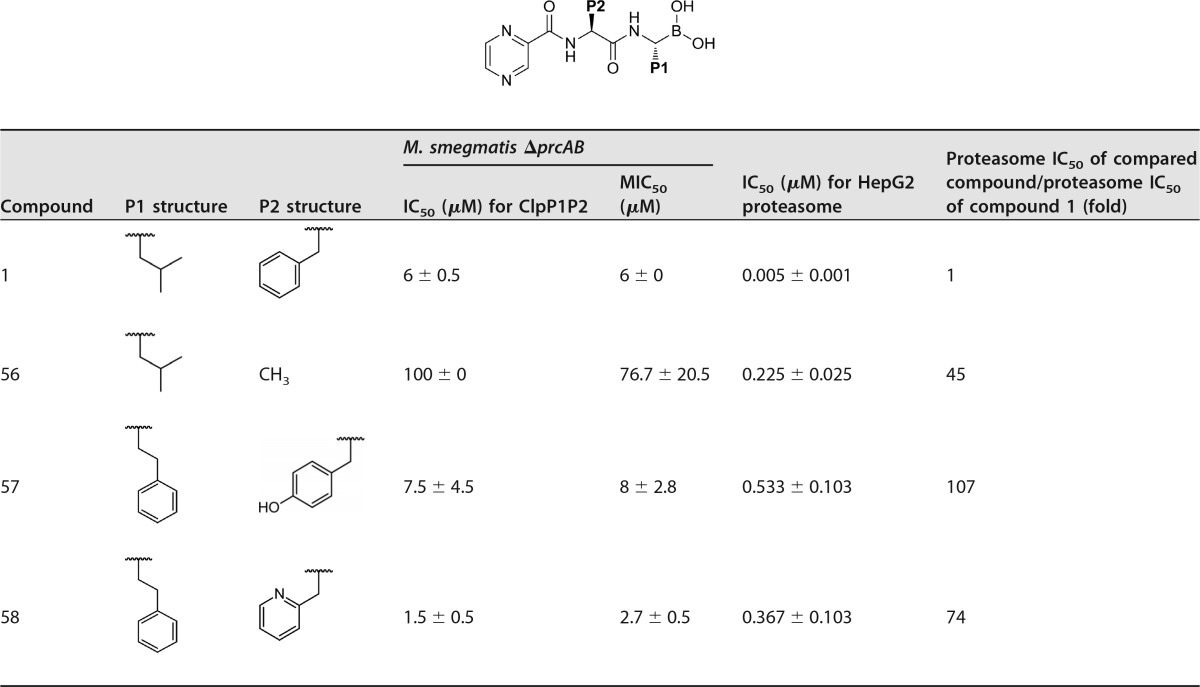
Derivatives of compound 1 with P1 and P2 modifications

### Activity against M. tuberculosis.

To confirm activity against M. tuberculosis, we tested a subset of the most promising compounds against M. tuberculosis H37Rv (Table 5). MIC_50_s and MIC_90_s followed the same trend against M. tuberculosis as they did against the M. smegmatis Δ*prcAB* strain used for SAR studies, with the exception of the MIC_50_ of compound 57, 0.8 μM, which represents an encouraging 5-fold improvement over results produced with compound 1. The MIC_90_ of compound 58 required a <3-fold-higher concentration (6 μM), while the other tested compounds required 3- to 6-fold-higher concentrations. Finally, we tested compound 58 for its bactericidal activity against M. tuberculosis. The MBC_99.9_ (minimum bactericidal concentration required to kill 99.9% of the bacterial population, i.e., to induce a 1,000-fold kill) was 50 μM against M. tuberculosis, similar to that of compound 1.

**TABLE 5:** Activities of preferred compounds against M. tuberculosis H37Rv^*a*^

Compound	MIC_50_ (μM)	MIC_90_ (μM)
1	4.3 ± 1.3^*b*^	12 ± 0.5^*b*^
31	6 ± 0	20 ± 0
32	6 ± 0	25 ± 0
51	1 ± 0.2	6 ± 0.2
57	0.8 ± 0	3 ± 0
58	2.5 ± 1.3^*b*^	6 ± 2.8

a MICs are an averages of 3 determinations (*n* = 3) unless otherwise stated.

b *n* = 6.

### Molecular modeling.

**FIG 9:**
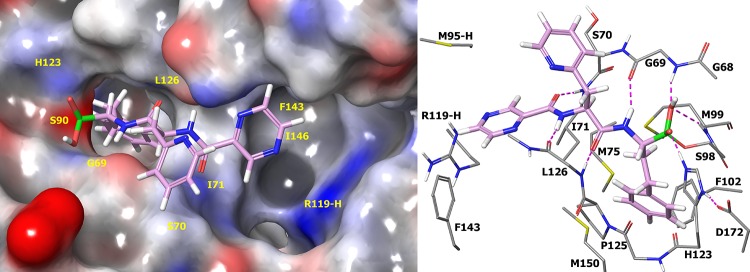
Docking of compound 58 into M. tuberculosis ClpP1 (PDB accession number 4U0G). Compound 58 is shown as a thick tube with a plum carbon. (Left) Electrostatic surface of M. tuberculosis ClpP1P2, with neutral charges in gray, positive partial charges in blue, and negative partial charges in red. (Right) M. tuberculosis ClpP1P2 is shown with selected residues shown as a thin tube with a gray carbon. Residues are from the same protein subunit unless marked by a suffix indicating the PDB chain. The P1 side chain is surrounded by hydrophobic S1 residues I71, M75, M99, F102, P125, L126, and M150.

Docking of compound 58 to the binding site of ClpP1P2 indicates that the hydrophobic S1 residues Ile71, Met75, Met99, Phe102, Pro125, Leu126, and Met150 make close contacts with the P1 phenethyl side chain (Fig. 9). Important hydrogen bonds are formed between the P2 amine and the backbone carbonyl of Leu126 and between the CAP carbonyl and the backbone amine of Ile71. The pyridyl P2 side chain is close to Ser70, and the CAP pyrazine group appears to be orientated in space, suggesting further potential for tuning of molecular properties.

### Protease selectivity.

ClpP1P2 is a serine protease, and as such, any inhibitor of ClpP1P2 has the potential to inhibit other serine proteases or other proteases of other classes, which in turn may lead to off-target effects or even toxicity. To assess the broader protease activity of preferred compound 58, we tested it against a panel of 62 diverse proteases representative of the proteome (Table 6).

**TABLE 6:** Protease selectivity panel results for compound 58

Protease^*a*^	IC_50_ (μM)
Chymase	5.75
Chymotrypsin	0.12
Kallikrein 1	6.03
Kallikrein 7	9.31
Plasma kallikrein	4.4
Proteinase A	1.94
Proteinase K	0.35

a Sixty-two proteases were tested in a 10-dose response, with the top concentration tested at 10 μM. Only IC_50_s are shown. The full panel of proteases is given in the supplemental material.

Only 2/62 (3%) of the panel were inhibited with a submicromolar IC_50_ and 5/62 (8%) with an IC_50_ between 1 to 10 μM. Both chymotrypsin and proteinase K are serine proteases with a preference for large aromatic or aliphatic P1 groups and were, not surprisingly, the most potently inhibited at IC_50_s of 120 and 350 nM, respectively. Of the others, which were inhibited in the micromolar range, chymase, kallikreins, and proteinase A/K are all serine proteases.

### *In vitro* ADME properties.

Compound 58 was selected for further profiling in *in vitro* absorption, distribution, metabolism, and excretion (ADME) assays (Table 7). Calculated parameters, such as molecular weight and the numbers of hydrogen bond donors and acceptors, are all in the accepted range for an oral small-molecule drug. The calculated LogP of the compounds (cLogP) is on the low side, in agreement with the topological polar surface area (TPSA), which is slightly high but not uncommonly so for antibacterial agents. These data explain the good aqueous solubility (>0.4 mg/ml at pH 7.4); however, permeability is still in the acceptable range, which is supported by the observed cellular potency. There is clearly space to increase the cLogP with further optimization, which may further improve permeability and maintain solubility in an acceptable range. However, Log D (octanol/PBS partition coefficient measured at pH 7.4) is in a preferred range of 2.96, so any increases in lipophilicity should be minimal in order to maintain the favorable solubility profile. Plasma protein binding (PPB) was determined to be moderate, with little difference between species. Bound levels of 90.46% (mouse) and 89.07% (human) indicate a significant free fraction. To confirm that antibacterial inhibitory potency was preserved in the presence of plasma, the MIC_50_ for Mycobacterium bovis BCG, with the addition of 10% fetal bovine serum (FBS), was determined to be 1.8 μM for compound 1 and 1.5 μM for compound 58. Human liver microsome stability was moderate, with a half-life of just over 24 min. However, high clearance in mouse microsomes was observed, with a half-life of about 8 min. Inhibition of cytochrome P450 enzymes was not detected at the highest concentration tested (10 μM), reducing concerns regarding drug-drug interactions, an important concern for an anti-TB drug, which is likely to be used in combination.

**TABLE 7:** Physicochemical properties and *in vitro* ADME parameters of compound 58

Property	Value
Mol wt	433.28
No. of HBD^*a*^	4
No. of HBA^*b*^	9
cLogP^*c*^	0.66
Log D (measured at pH 7.4)	2.96
TPSA (Å^2^)^*d*^	137
Aq solubility (μg/ml) at pH 7.4^*e*^	449.8
Aq solubility (μg/ml) at pH 4^*e*^	421.5
PAMPA *P_e_* (× 10^−6^ cm/s)^*f*^	3.5
Plasma protein binding in mice (%)	90.46
Plasma protein binding in humans (%)	89.07
HLM *t*_1/2_ (min)^*g*^	24.29
MLM *t*_1/2_ (min)^*h*^	8.15
CYP450 3A4 IC_50_ (μM)^*i*^	>10
CYP450 2D6 IC_50_ (μM)^*i*^	>10

a HBD, hydrogen bond donors.

b HBA, hydrogen bond acceptors.

c Calculated as miLogP using Molinspiration property engine v2014.11 (http://www.molinspiration.com).

d TPSA, topological polar surface area (http://www.molinspiration.com).

e Thermodynamic aqueous (Aq) solubility.

f PAMPA, parallel artificial-membrane permeability assay; *P_e_*, permeability.

g HLM, human liver microsomes.

h MLM, mouse liver microsomes.

i CYP450, recombinant cytochrome P450 enzyme assay.

### Cytotoxicity data.

The 50% growth inhibition concentration (GI_50_; a determination of the minimal concentration of a compound that inhibits the growth of the cells by 50%) of compounds 1 and 58 were determined against Vero or HepG2 cells and found to be 250 or 400 μM (compound 1) and 500 or 500 μM (compound 58), respectively, by an MTS [3-(4,5-dimethylthiazol-2-yl)-5-(3-carboxymethoxyphenyl)-2-(4-sulfophenyl)-2H-tetrazolium] assay. This provides for a large therapeutic window in cells and reduces toxicity concerns.

### *In vivo* pharmacokinetics.

**FIG 10:**
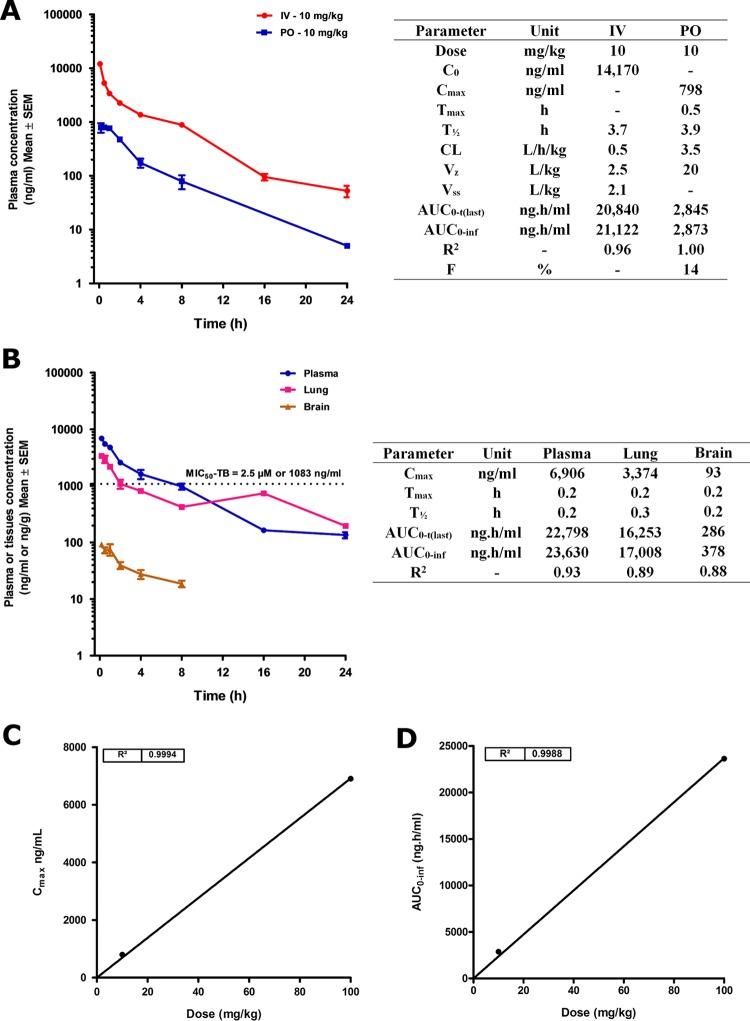
Intravenous/oral pharmacokinetic profile for compound 58 in mice (3 mice per time point). (A) i.v./p.o. concentrations of compound 58 plotted against time for up to 24 h, and pharmacokinetic parameters at a dose of 10 mg/kg; (B) tissue distribution plot of concentrations against time and pharmacokinetic parameters for orally administered compound 58 in plasma, lung, and brain at the higher dose of 100 mg/kg; (C) dose linearity plot of *C*_max_ versus dose; (D) dose linearity plot of AUC_0–inf_ versus dose. CL, clearance; F, variance ratio.

The pharmacokinetic profile of compound 58 was determined in mice following a single intravenous (i.v.) dose of 10 mg/kg of body weight and single oral doses of 10 and 100 mg/kg (Fig. 10A). At 10 mg/kg i.v., compound 58 reached a maximum concentration of 14,170 ng/ml (32.7 μM), well above the required MIC_50_ for activity against M. tuberculosis. The plasma concentration of compound 58 declined over time, with a mean elimination half-life of 3.7 h. The systemic plasma clearance was moderate at 0.5 liter/h/kg. The volumes of distribution at steady state (*V_ss_*) and during the terminal phase (*V_z_*) were 2.1 and 2.5 liter/kg, respectively, with an area under the concentration-time curve from time zero to the last time point [AUC_0–*t*(last)_] being 20,840 ng · h/ml. Overall, concentrations of compound 58 remained above its anti-M. tuberculosis MIC_50_ for approximately 4 h. No acute side effects were observed during tail vein injection in this study. No abnormal clinical signs were observed for up to 24 h after injection.

Following oral administration at 10 mg/kg in mice, concentrations of compound 58 could be quantified for up to 24 h. The mean maximal plasma concentration (*C*_max_) was 798 ng/ml, observed at 0.5 h after dosing, indicating rapid absorption. The mean exposure as calculated by an AUC from time zero to infinity (AUC_0–inf_) was 2,873 ng · h/ml, resulting in an absolute oral bioavailability of 14%. Although bioavailability was low, significant exposures were achieved orally, suggesting that higher doses may result in higher oral exposures, assuming dose linearity. The highest tolerated dose that is likely to be employed to maximize efficacy and dose linearity is an important concern for an antibacterial agent; hence, the higher dose of 100 mg/kg was studied with a determination of tissue concentrations in lung and brain (Fig. 10B). At 100 mg/kg, high concentrations of compound 58 were quantifiable for up to 24 h in plasma and lung and for up to 8 h in brain. The *C*_max_ was 6,906 ng/ml in plasma, 3,374 ng/g in lung, and 93 ng/g in brain. Peak concentrations were observed in a short time (time to maximum concentration of a drug in serum [*T*_max_] = 10 min), indicating rapid absorption. The mean exposure as calculated from the AUC_0–*t*(last)_ was 22,798 ng · h/ml in plasma, 16,253 ng · h/g in lung, and 286 ng · h/g in brain. Plasma concentrations were above the murine M. tuberculosis MIC_50_ for about 8 h. Lung concentrations were above the murine M. tuberculosis MIC_50_ for about 2 to 3 h but persisted longer than in plasma (half-life [*t*_1/2_] = 0.3 h), indicating good penetration of the target organ. Concentrations in brain, although measurable, were much lower than in plasma or lung and not quantifiable after 8 h. Dose linearity was very good, as assessed by comparing the plasma concentrations at 10 and 100 mg/kg for both the *C*_max_ (Fig. 10C) and the AUC to infinity (AUC_0–inf_) (Fig. 10D). The maximum tolerated dose was not determined. This preliminary work supports compound 58 as a drug-like template suitable for further optimization.

### Concluding remarks.

In this work, a series of novel analogues of the potent human-proteasome inhibitor, compound 1, were prepared as mycobacterial caseinolytic protease ClpP1P2 inhibitors. Compounds were characterized in a ClpP1P2 target-based cell reporter assay, confirming protease inhibition and, importantly, bacterial-cell penetration. All compounds were also tested in an antibacterial assay using a proteasome knockout strain of M. smegmatis, allowing growth activity SAR to be established without interference from bacterial-proteasome inhibition. In most cases, growth inhibition tracked ClpP1P2 activity, reassuring us that there were no other off-target effects. A key focus of this study was to gain an understanding of human-proteasome SAR in an effort to identify the first lead compounds with reduced proteasome activity, and hence less toxicity, while retaining ClpP1P2 activity. A preferred compound, compound 58, had low, single-digit, micromolar ClpP1P2 and growth-inhibitory activity while having 74-fold-reduced potency against the human proteasome. The growth-inhibitory potency of compound 58 against M. tuberculosis was gratifyingly in the same low-micromolar-concentration range, while GI_50_s were 200-fold higher in both the Vero and HepG2 cell lines. Compound 58 was active (<10 μM) against only 11% of a panel of 62 proteases and active (<1 μM) against only 2 proteases (3% of the panel). Compound 58 has favorable *in vitro* ADME properties for an antibacterial agent, including moderate protein binding, indicated by its MIC not being affected by additional serum. Oral/i.v. pharmacokinetics indicated moderate clearance and low bioavailability. However, oral exposures were linear between 10 and 100 mg/kg with plasma concentrations above the murine M. tuberculosis MIC_50_ for up to 8 h at the highest dose tested. This work demonstrates that potent inhibitors of ClpP1P2 are possible with reduced proteasome activity and paves the way for further studies, ultimately leading to new treatment options for drug-resistant M. tuberculosis.

## MATERIALS AND METHODS

### Chemistry and general experimental details.

All reactions requiring anhydrous conditions were carried out under a nitrogen atmosphere using oven-dried glassware (105°C), which was cooled under vacuum. All reaction solvents used, such as dichloromethane, toluene, and diethyl ether, were freshly collected from a solvent purification system (PureSolv MD-4; Innovative Technology, MA). All final compounds (boronic acid) were stored in a −20°C freezer under nitrogen. Proton nuclear magnetic resonance (^1^H NMR) and carbon NMR (^13^C NMR) spectra were recorded in CDCl_3_, CD_3_OD, and dimethyl sulfoxide-d_6_ (DMSO-d_6_), unless otherwise stated. ^1^H NMR (400 MHz) and ^13^C NMR (101 MHz) with complete proton decoupling were performed on a 400-MHz Bruker Ultra Shield NMR spectrometer. Chemical shifts were reported as δ in units of parts per million downfield from tetramethylsilane (δ, 0.00), with the residual solvent signal used as an internal standard, as follows: DMSO (2.50 ppm for ^1^H and 39.5 ppm for ^13^C NMR), CDCl_3_ (7.26 ppm for ^1^H and 77.2 ppm for ^13^C NMR), and CD_3_OD (3.31 ppm for ^1^H and 49.0 ppm for ^13^C NMR). Multiplicities were abbreviated as s (singlet), d (doublet), t (triplet), q (quartet), quintet, m (multiplet), dd (doublet of doublets), ddd (doublet of doublet of doublets), dt (doublet of triplets), dq (doublet of quartets), dm (doublet of multiplets), and br (broad). Coupling constants (*J*) were recorded in hertz. The ^13^C signal for the boron-attached carbon was very weak and broad, which was observed by heteronuclear multiple-quantum-coherence (HMQC) NMR. High-resolution electrospray ionization (ESI) mass spectra were obtained on a Bruker micrOTOF-Q II mass spectrometer. All tested compounds were >95% pure, as determined by reverse-phase high-pressure liquid chromatography (HPLC) on a Shimadzu SPD-20A HPLC system. The test compound was dissolved in methanol (MeOH) and injected through a 100-μl loop at a flow rate of 1.0 ml/min, with UV detection at 254 and 220 nm. Separation was carried out on a Zorbax SB-C_18_ column (250 mm by 4.6 mm, with a 5-μm inside diameter; Agilent). The purity of each compound was assessed from the area of the major peak in comparison with the total area of peaks obtained on the chromatogram. Boronic acids undergo facile dehydration and/or decomposition upon being heated (28). Therefore, melting points of boronic acids were not determined. All commercial reagents were purchased from Sigma-Aldrich, Fluka, Alfa Aesar, Merck, TCI, or Acros and were of the highest purity grade available. They were used without further purification unless specified. Compound 1 was purchased from Selleckchem.

### Compound 25.

Compound 25 was (*R*)-(3-methyl-1-(3-phenylpropanamido)butyl)boronic acid. To a round-bottom flask, 2-methylpropylboronic acid (222 mg, 2.17 mmol, 5.0 eq) and 1 N HCl (1.0 ml) were added to a biphasic mixture of ester 19 (150 mg, 0.437 mmol, 1.0 eq) in methanol (3 ml) and pentane (3 ml). The round-bottom flask was closed tightly, and the biphasic mixture was stirred vigorously at room temperature (rt) for 18 h. The reaction mixture was diluted with methanol (50 ml). The methanolic phase was washed with hexane (50 ml three times), and the combined hexane layer was extracted with methanol (50 ml once). The combined methanolic layers were evaporated *in vacuo*. The residue was purified by reverse-phase HPLC (80/20 MeOH-H_2_O [0.1% formic acid]) to give compound 25. It is a white solid (yield, 16%). ^1^H NMR (400 MHz, CD_3_OD) δ 7.45 to 6.87 (m, 5H), 2.99 (t, *J* = 7.4 Hz, 2H), 2.74 to 2.67 (m, 2H), 2.57 (t, *J* = 7.6 Hz, 1H), 1.61 to 1.45 (m, 1H), 1.22 (dd, *J* = 8.0, 6.7 Hz, 2H), and 0.88 (d, *J* = 6.6 Hz, 6H). ^13^C NMR (101 MHz, CD_3_OD) δ 179.3, 140.8, 129.6 (2C), 129.5 (2C), 127.6, 45.8, 41.0, 32.1, 26.9, 23.9, 22.8, 22.1. The low-resolution mass spectrometry (LRMS)-ESI *m/z* was 246.0 [M − H_2_O + H]^+^.

By using a procedure similar to that used for compound 25, the following boronic acids were synthesized. Note that final isolated yields were generally low due to the required preparative HPLC purification on a small scale.

### Compound 26.

Compound 26 was ((*R*)-1-((*S*)-2-amino-3-phenylpropanamido)-3-methylbutyl)boronic acid. This compound is a white solid (yield, 21%). ^1^H NMR (400 MHz, CD_3_OD) δ 7.41 (m, 5H), 4.14 (t, *J* = 7.0 Hz, 1H), 3.14 (t, *J* = 6.7 Hz, 2H), 3.05 (t, *J* = 7.5 Hz, 1H), 1.8 to 1.39 (m, 1H), 1.27 (t, *J* = 6.6 Hz, 2H), 0.87 (d, *J* = 6.5 Hz, 6H). ^13^C NMR (101 MHz, CD_3_OD) δ 170.4, 135.3, 130.6 (2C), 130.1 (2C), 128.9, 54.5, 40.9, 38.6, 26.6, 23.6, 22.2. The liquid chromatography mass spectrometry (LCMS)-ESI *m/z* was 277.1 [M − H]^+^.

### Compound 27.

Compound 27 was ((*R*)-1-((*S*)-3-phenyl-2-(pyrazine-2-carboxamido)propanamido)hexyl)boronic acid. It is a white solid (yield, 14%). ^1^H NMR (400 MHz, CD_3_OD) δ 9.17 (d, *J* = 1.5 Hz, 1H), 8.79 (d, *J* = 2.5 Hz, 1H), 8.69 (dd, *J* = 2.5, 1.5 Hz, 1H), 7.37 to 7.16 (m, 5H), 5.04 (t, *J* = 7.5 Hz, 1H), 3.32 to 3.15 (m, 2H), 2.56 (t, *J* = 7.9 Hz, 1H), 1.52 to 1.09 (m, 8H), 0.89 (t, *J* = 7.0 Hz, 3H). ^13^C NMR (101 MHz, CD_3_OD) δ 176.7, 165.1, 148.9, 144.8, 137.1, 130.5 (2C), 129.7 (2C), 128.2, 53.0, 46.8, 38.6, 33.1, 31.7, 28.4, 23.6, 14.4. The LCMS-ESI *m/z* was 397.2 [M − H]^+^.

### Compound 28.

Compound 28 was ((*R*)-cyclohexyl((*S*)-3-phenyl-2-(pyrazine-2-carboxamido)propanamido)methyl)boronic acid. It is a white solid (yield, 36%). ^1^H NMR (400 MHz, CD_3_OD) δ 9.19 (s, 1H), 8.80 (s, 1H), 8.70 (s, 1H), 7.42 to 7.05 (m, 5H), 5.05 (t, *J* = 7.7 Hz, 1H), 3.24 (d, *J* = 7.7 Hz, 2H), 2.34 (d, *J* = 8.9 Hz, 1H), 1.83 to 0.40 (m, 11H). ^13^C NMR (101 MHz, CD_3_OD) δ 176.5, 165.3, 148.5, 144.8, 137.1, 130.5 (2C), 129.0 (2C), 128.2, 53.1, 51.8, 39.5, 38.9, 32.4, 31.8, 27.6, 27.3, 27.2. The LCMS-ESI *m/z* was 409.0 [M − H]^+^.

### Compound 29.

Compound 29 was ((*R*)-2-cyclohexyl-1-((*S*)-3-phenyl-2-(pyrazine-2-carboxamido)propanamido)ethyl)boronic acid. It is a white solid (yield, 15%). The diastereomeric (dr) ratio was 3.5:1 (dr mixture at P1's center). The major-isomer data were as follows: ^1^H NMR (400 MHz, CD_3_OD) δ 9.18 (d, *J* = 1.3 Hz, 1H), 8.80 (d, *J* = 2.5 Hz, 1H), 8.71 to 8.67 (m, 1H), 7.36 to 7.18 (m, 5H), 5.04 (t, *J* = 7.7 Hz, 1H), 3.25 (dd, *J* = 7.7, 4.3 Hz, 2H), 2.73 (dd, *J* = 9.3, 5.8 Hz, 1H), 1.86 to 1.56 (m, 4H), 1.40 to 1.01 (m, 5H), 0.91 to 0.65 (m, 4H). ^13^C NMR (101 MHz, CD_3_OD) δ 176.7, 165.1, 148.9, 145.6, 144.8, 137.0, 130.5 (2C), 129.7 (2C), 128.3, 52.9, 43.5, 39.3, 38.7, 36.2, 35.3, 33.6, 27.7, 27.4, 27.2. The LCMS-ESI *m/z* was 423.2 [M − H]^+^.

### Compound 30.

Compound 30 was ((*R*)-3-cyclohexyl-1-((*S*)-3-phenyl-2-(pyrazine-2-carboxamido)propanamido)propyl)boronic acid. It is a white solid (yield, 19%). ^1^H NMR (400 MHz, CD_3_OD) δ 9.18 (s, 1H), 8.80 (d, *J* = 1.9 Hz, 1H), 8.69 (s, 1H), 7.33 to 7.09 (m, 5H), 5.04 (t, *J* = 7.6 Hz, 1H), 3.28 to 3.11 (m, 2H), 2.53 (dd, *J* = 8.2, 6.5 Hz, 1H), 1.72 to 1.60 (m, 5H), 1.51 to 1.30 (m, 1H), 1.32 to 0.97 (m, 8H), 0.95 to 0.72 (m, 2H). ^13^C NMR (101 MHz, CD_3_OD) δ 176.7, 165.1, 148.9, 144.8, 137.1, 130.5 (2C), 129.7 (2C), 128.3, 53.0, 47.1, 39.1, 38.6, 36.4, 34.5, 34.5, 29.2, 27.8, 27.5. The LCMS-ESI *m/z* was 437.2 [M − H]^+^. The high-resolution mass spectrometry (HRMS)–ESI *m/z* [M − H]^+^ were 437.2370 (calculated for C_23_H_30_BN_4_O_4_) and 437.2375 (found).

### Compound 31.

Compound 31 was ((*R*)-2-phenyl-1-((*S*)-3-phenyl-2-(pyrazine-2-carboxamido)propanamido)ethyl)boronic acid. It is a white solid (yield, 20%). ^1^H NMR (400 MHz, CD_3_OD) δ 9.17 (s, 1H), 8.79 (d, *J* = 2.3 Hz, 1H), 8.68 (s, 1H), 7.41 to 7.20 (m, 5H), 7.17 (t, *J* = 7.2 Hz, 2H), 7.10 (t, *J* = 6.4 Hz, 1H), 7.01 (d, *J* = 7.0 Hz, 2H), 5.01 (t, *J* = 8.0 Hz, 1H), 3.29 to 3.17 (m, 2H), 2.88 (dd, *J* = 9.8, 5.5 Hz, 1H), 2.79 (dd, *J* = 13.9, 5.5 Hz, 1H), 2.43 (dd, *J* = 13.9, 9.8 Hz, 1H). ^13^C NMR (101 MHz, CD_3_OD) δ 177.0, 165.1, 148.9, 145.5, 144.8, 144.8, 141.8, 137.1, 130.5 (2C), 129.9 (2C), 129.8 (2C), 129.3 (2C), 128.3, 127.0, 53.0, 38.6, 38.0 (2C). The LCMS-ESI *m/z* was 417.0 [M − H]^+^. The HRMS-ESI *m/z* [M − H]^+^ were 417.1744 (calculated for C_22_H_22_BN_4_O_4_) and 417.1746 (found).

### Compound 32.

Compound 32 was ((*R*)-3-phenyl-1-((*S*)-3-phenyl-2-(pyrazine-2-carboxamido)propanamido)propyl)boronic acid. It is a white solid (yield, 24%). ^1^H NMR (400 MHz, CD_3_OD) δ 9.18 (s, 1H), 8.80 (s, 1H), 8.70 (s, 1H), 7.36 to 7.25 (m, 4H), 7.26 to 7.16 (m, 3H), 7.18 to 7.07 (m, 3H), 5.06 (t, *J* = 7.6 Hz, 1H), 3.29 to 3.20 (m, 2H), 2.61 (dd, *J* = 8.0, 6.6 Hz, 1H), 2.57 to 2.44 (m, 2H), 1.82 to 1.64 (m, 1H), 1.61 to 1.47 (m, 1H). ^13^C NMR (101 MHz, CD_3_OD) δ 177.0, 165.2, 148.9, 145.6, 144.8, 143.6, 137.1, 130.5 (2C), 129.7 (2C), 129.4 (2C), 129.2 (2C), 128.3 (2C), 126.7, 53.0, 46.1, 38.5, 34.8, 33.8. The LCMS-ESI *m/z* was 431.1 [M − H]^+^. The HRMS-ESI *m/z* [M– H]^+^ were 431.1901 (calculated for C_23_H_24_BN_4_O_4_) and 431.1900 (found).

### Compound 33.

Compound 33 was ((*R*)-1-((*S*)-2-acetamido-3-phenylpropanamido)-3-methylbutyl)boronic acid. It is a white solid (yield, 15%). ^1^H NMR (400 MHz, CD_3_OD) δ 7.36 to 7.16 (m, 5H), 4.75 (t, *J* = 8.0 Hz, 1H), 3.12 to 2.98 (m, 2H), 2.62 (dd, *J* = 8.8, 6.5 Hz, 1H), 1.94 (s, 3H), 1.39 to 1.27 (m, 1H), 1.18 to 1.06 (m, 2H), 0.81 (d, *J* = 7.8 Hz, 6H). ^13^C NMR (101 MHz, CD_3_OD) δ 177.4, 173.2, 137.1, 130.4 (2C), 129.6 (2C), 128.2, 52.7, 44.8, 40.9, 38.6, 26.7, 23.8, 22.2, 22.0. The LCMS-ESI *m/z* was 319.1 [M − H]^+^.

### Compound 34.

Compound 34 was ((*R*)-3-methyl-1-((*S*)-3-phenyl-2-(2-phenylacetamido)propanamido)butyl)boronic acid. It is a white solid (yield, 18%). The dr ratio was 7.3:1 (dr mixture at P2's center). The major-isomer data were as follows. ^1^H NMR (400 MHz, CD_3_OD) δ 7.40 to 7.08 (m, 10H), 4.77 (t, *J* = 7.8 Hz, 1H), 3.50 (s, 2H), 3.14 to 2.98 (m, 2H), 2.60 (t, *J* = 8.0 Hz, 1H) 1.38 to 1.28 (m, 1H), 1.12 (t, *J* = 7.0 Hz, 2H), 0.82 (d, *J* = 6.5 Hz, 6H). ^13^C NMR (101 MHz, CD_3_OD) δ 177.3, 173.8, 137.0, 136.4, 130.4 (2C), 130.3, 130.1 (2C), 129.7 (2C), 129.6, 129.5 (2C), 128.2, 127.9, 44.6, 43.3, 40.8, 38.6, 26.7, 23.8, 22.0. The LCMS-ESI *m/z* was 395.1 [M − H]^+^.

### Compound 35.

Compound 35 was ((*R*)-1-((*S*)-2-benzamido-3-phenylpropanamido)-3-methylbutyl)boronic acid. It is a white solid (yield, 18%). ^1^H NMR (400 MHz, CD_3_OD) δ 7.80 to 7.75 (m, 2H), 7.58 to 7.51 (m, 1H), 7.48 to 7.42 (m, 2H), 7.31 (apparent d, *J* = 4.4 Hz, 4H), 7.27 to 7.20 (m, 1H), 4.97 (t, *J* = 8.0 Hz, 1H), 3.24 to 3.16 (m, 2H), 2.66 (t, *J* = 7.6 Hz, 1H), 1.42 to 1.28 (m, 1H), 1.16 (t, *J* = 7.3 Hz, 2H), 0.84 (d, *J* = 6.5 Hz, 6H). ^13^C NMR (101 MHz, CD_3_OD) δ 177.6, 170.3, 137.3, 134.9, 133.0, 130.5 (2C), 129.7 (2C), 129.5 (2C), 128.5 (2C), 128.2, 53.3, 43.3, 40.8, 38.4, 26.7, 23.8, 22.0 The LCMS-ESI *m/z* was 365.0 [M – H_2_O + H] ^+^. The HRMS (ESI) *m/z* [M − H]^+^ were 381.1995 (calculated for C_21_H_26_BN_2_O_4_) and 381.1999 (found).

### Compound 36.

Compound 36 was ((*R*)-3-methyl-1-((*S*)-2-(nicotinamido)-3-phenylpropanamido)butyl)boronic acid. It is a white solid (yield, 11%). ^1^H NMR (400 MHz, CD_3_OD) δ 9.13 (br s, 1H), 8.91 (br s, 1H), 8.51 (d, *J* = 8.0 Hz, 1H), 7.84 (s, 1H), 7.43 to 7.08 (m, 5H), 4.99 (t, *J* = 8.1 Hz, 1H), 3.28 to 3.11 (m, 2H), 2.68 (dd, *J* = 8.4, 6.9 Hz, 1H), 1.50 to 1.24 (m, 1H), 1.23 to 1.06 (m, 2H), 0.84 (d, *J* = 6.5 Hz, 6H). ^13^C NMR (101 MHz, CD_3_OD) δ 177.0, 166.2, 149.9, 146.8, 140.6, 137.1, 130.4 (2C), 129.8 (2C), 128.3, 53.5, 44.5, 40.9, 38.4, 26.7, 23.8, 22.0. The LCMS-ESI *m/z* was 366.0 [M –H_2_O + H]^+^. The HRMS-ESI *m/z* [M − H]^+^ were 382.1947 (calculated for C_20_H_25_BN_3_O_4_) and 382.1943 (found).

### Compound 37.

Compound 37 was ((*R*)-3-methyl-1-((*S*)-3-phenyl-2-(picolinamido)propanamido)butyl)boronic acid. It is a white solid (yield, 11%). ^1^H NMR (400 MHz, CD_3_OD) δ 8.55 (br s, 1H), 7.88 (br s, 2H), 7.49 (br s, 1H), 7.51 to 6.95 (m, 7H), 4.91 (br s, 1H), 2.58 (t, *J* = 7.3 Hz, 1H), 1.38 to 1.16 (m, 2H), 1.09 (t, *J* = 7.0 Hz, 2H), 0.75 (t, *J* = 5.4 Hz, 6H). ^13^C NMR (101 MHz, CD_3_OD) δ 177.0, 166.4, 150.3, 149.9, 138.8, 137.0, 130.5 (2C), 129.7 (2C), 129.5, 128.3, 123.3, 52.9, 43.4, 40.9, 38.9, 26.7, 23.8, 22.1. The LCMS-ESI *m/z* was 382.0 [M − H]^+^.

### Compound 38.

Compound 38 was ((*R*)-3-methyl-1-((*S*)-3-phenyl-2-(2-phenylpyrimidine-5-carboxamido)propanamido)butyl)boronic acid. It is a white solid (yield, 22%). The dr ratio was 6:1 (dr mixture at P2's center). The major-isomer data were as follows. ^1^H NMR (400 MHz, CD_3_OD) δ 9.15 (s, 2H), 8.55 to 8.43 (m, 2H), 7.66 to 7.40 (m, 3H), 7.37 to 7.19 (m, 5H), 5.01 (t, *J* = 8.1 Hz, 1H), 3.31 to 3.16 (m, 2H), 2.73 to 2.60 (m, 1H), 1.46 to 1.27 (m, 2H), 0.85 (d, *J* = 6.7 Hz, 6H). ^13^C NMR (101 MHz, CD_3_OD) δ 177.1, 167.5, 166.3, 157.8 (2C), 137.9, 137.2, 132.7, 130.5 (2C), 130.3, 129.7 (4C), 128.3, 126.33, 53.3, 44.7, 40.9, 38.4, 26.7, 23.8, 22.0. The LCMS-ESI *m/z* was 459.0 [M – H]^+^. The HRMS-ESI *m/z* [M − H]^+^ were 459.2214 (calculated for C _25_H_28_BN_4_O_4_) and 459.2216 (found).

### Compound 39.

Compound 39 was ((*R*)-3-methyl-1-((*S*)-3-phenyl-2-(6-phenylpyrimidine-4-carboxamido)propanamido)butyl) boronic acid. It is a white solid (yield, 19%). ^1^H NMR (400 MHz, CD_3_OD) δ 9.29 (s, 1H), 8.45 (s, 1H), 8.24 to 8.16 (m, 2H), 7.62 to 7.50 (m, 3H), 7.38 to 7.18 (m, 5H), 5.06 (t, *J* = 7.6 Hz, 1H), 3.28 (dd, *J* = 7.5, 5.8 Hz, 2H), 2.71 (t, *J* = 7.6 Hz, 1H), 1.51 to 138 (m, 1H), 1.21 (t, *J* = 8.8 Hz, 2H), 0.85 (d, *J* = 6.6 Hz, 6H). ^13^C NMR (101 MHz, CD_3_OD) δ 176.6, 167.6, 165.1, 159.40, 157.9, 137.2, 137.0, 132.8, 130.5 (2C), 130.2 (2C), 129.7 (2C), 128.5 (2C), 128.3, 115.0, 53.1, 44.4, 40.9, 38.7, 26.7, 23.7, 22.2. The LCMS-ESI *m/z* was 459.2 [M − H]^+^.

### Compound 40.

Compound 40 was ((*R*)-1-((*S*)-2-(indolizine-2-carboxamido)-3-phenylpropanamido)-3-methylbutyl)boronic acid. It is a white solid (yield, 21%). The dr ratio was 3.4:1 (dr mixture at P2's center). ^1^H NMR (400 MHz, CD_3_OD) δ 8.08 (d, *J* = 7.0 Hz, 1H), 7.86 (s, 1H), 7.40 to 7.16 (m, 7H), 6.82 to 6.66 (m, 2H), 6.58 (dd, *J* = 6.6 Hz, 1H), 4.98 (t, *J* = 8.0 Hz, 1H), 3.22 (dd, *J* = 8.0, 2.5 Hz, 2H), 2.65 (t, *J* = 8.0 Hz, 1H), 1.44 to 1.20 (m, 1H), 1.15 (dd, *J* = 8.0, 6.4 Hz, 2H), 0.84 (t, *J* = 6.6 Hz, 5H). ^13^C NMR (101 MHz, CD_3_OD) δ 177.7, 167.5, 137.4, 134.28, 130.5 (2C), 129.7 (2C), 128.2, 126.8, 120.8, 119.4, 113.1, 52.9, 45.0, 40.9, 38.5, 26.7, 23.9, 22.0. The LCMS-ESI *m/z* was 420.2 [M − H]^+^.

### Compound 41.

Compound 41 was ((*R*)-1-((*S*)-2-(indolizine-3-carboxamido)-3-phenylpropanamido)-3-methylbutyl)boronic acid. It is a gray solid (yield, 15%). The dr ratio was 4.4: 1 (dr mixture at P2's center). The major-isomer data were as follows. ^1^H NMR (400 MHz, CD_3_OD) δ 9.41 (d, *J* = 7.2 Hz, 1H), 7.55 to 7.50 (m, 2H), 7.36 to 7.27 (m, 5H), 7.26 to 7.20 (m, 1H), 6.98 (ddd, *J* = 8.9, 6.7, 1.1 Hz, 1H), 6.78 to 6.72 (m, 1H), 6.50 (d, *J* = 4.8 Hz, 1H), 4.98 (t, *J* = 7.9 Hz, 1H), 3.23 (dd, *J* = 7.9, 4.5 Hz, 2H), 2.66 (t, *J* = 7.6 Hz, 1H), 1.44 to 1.31 (m, 1H), 1.16 (t, *J* = 7.4 Hz, 2H), 0.84 (d, *J* = 7.5, Hz, 6H). ^13^C NMR (101 MHz, CD_3_OD) δ 178.2, 166.4, 163.7, 138.8 (2C), 137.5 (2C), 130.5, 129.7, 128.1, 128.0, 122.0, 119.9, 119.2, 113.3, 101.7, 52.6, 43.7, 40.9, 38.6, 26.7, 23.8, 22.0. The LCMS-ESI *m/z* was 420.0 [M − H]^+^.

### Compound 42.

Compound 42 was ((*R*)-3-methyl-1-((*S*)-3-phenyl-2-(5-phenyloxazole-2-carboxamido)propanamido)butyl)boronic acid. It is a white solid (yield, 16%). The dr ratio was 6:1 (dr mixture at P2's center). The major-isomer data were as follows. ^1^H NMR (400 MHz, CD_3_OD) δ 7.82 (d, *J* = 8.0 Hz, 2H), 7.67 (s, 1H), 7.51 to 7.39 (m, 3H), 7.34 to 7.20 (m, 5H), 4.99 (t, *J* = 7.8 Hz, 1H), 3.29 to 3.19 (m, 2H), 2.69 (t, *J* = 7.2 Hz, 1H), 1.46 to 1.31 (m, 1H), 1.22 to 1.16 (m, 2H), 0.85 (d, *J* = 6.5 Hz, 6H). ^13^C MR (101 MHz, CD_3_OD) δ 176.6, 157.0, 155.4, 154.8, 137.0, 130.8, 130.5 (2C), 130.2 (2C), 129.7, 128.3, 128.2, 126.0 (2C), 124.4, 53.1, 49.8, 44.5, 40.9, 38.3, 26.7, 23.8, 22.1. The LCMS-ESI *m/z* was 448.0 [M − H]^+^.

### Compound 43.

Compound 43 was ((*R*)-1-((*S*)-2-(benzo[*d*]thiazole-7-carboxamido)-3-phenylpropanamido)-3-ethylbutyl) boronic acid. It is a white solid (yield, 21%). ^1^H NMR (400 MHz, CD_3_OD) δ 9.32 (s, 1H), 8.25 (d, *J* = 8.1 Hz, 1H), 8.09 (d, *J* = 7.5 Hz, 1H), 7.69 (dd, *J* = 7.8, 7.8 Hz, 1H), 7.39 to 7.19 (m, 5H), 5.02 (t, *J* = 8.1 Hz, 1H), 3.27 (d, *J* = 8.5 Hz, 2H), 2.68 (t, *J* = 7.6 Hz, 1H), 1.42 to 1.32 (m, 1H), 1.16 (t, *J* = 7.4 Hz, 2H), 0.84 (d, *J* = 6.6 Hz, 6H). ^13^C NMR (101 MHz, CD_3_OD) δ 177.4, 167.8, 161.0, 155.0, 137.2, 134.3, 130.5 (2C), 130.3, 129.7 (2C), 129.6, 128.5, 128.2, 127.5, 127.3, 125.2, 53.6, 44.6, 41.0, 38.4, 26.5, 23.9, 22.0. The LCMS-ESI *m/z* was 438.1 [M-H]^+^. The HRMS-ESI *m/z* [M − H]^+^ were 438.1668 (calculated for C_22_H_25_BN_3_O_4_S) and 438.1663 (found).

### Compound 44.

Compound 44 was ((*R*)-3-methyl-1-((*S*)-3-phenyl-2-((*S*)-3-phenyl-2-(pyrazine-2-carboxamido)propanamido)butyl)boronic acid. It is a white solid (yield, 13%). ^1^H NMR (400 MHz, CD_3_OD) δ 9.14 (s, 1H), 8.78 (s, 1H), 8.66 (s, 1H), 7.35 to 7.11 (m, 10H), 4.84 to 4.80 (m, 2H), 3.26 to 2.98 (m, 4H), 2.66 (t, *J* = 7.5 Hz, 1H), 1.49 to 1.36 (m, 1H), 1.16 (t, *J* = 7.2 Hz, 2H), 0.85 (d, *J* = 7.1 Hz, 6H). ^13^C NMR (101 MHz, CD_3_OD) δ 176.9, 173.0, 164.9, 148.8, 145.6, 144.8, 144.7, 137.8, 137.0, 130.4 (2C), 130.3 (2C), 129.7 (2C), 129.5 (2C), 128.1, 128.0, 55.9, 52.7, 44.5, 40.9, 38.8, 38.6, 26.7, 23.9, 22.0. The LCMS-ESI *m/z* was 530.0 [M − H]^+^.

### Compound 45.

Compound 45 was ((*R*)-3-methyl-1-((*S*)-3-phenyl-2-(piperidine-1-carboxamido)propanamido)butyl)boronic acid. It is a white solid (yield, 20%). ^1^H NMR (400 MHz, CD_3_OD) δ 7.36 to 6.94 (m, 5H), 4.57 (t, *J* = 8.0 Hz, 1H), 3.10 to 2.87 (m, 2H), 2.55 (t, *J* = 8.0 Hz, 1H), 1.58 to 1.20 (m, 11H), 1.12 to 1.02 (m, 2H), 0.77 (d, *J* = 4.7 Hz, 3H), 0.75 (d, *J* = 4.7 Hz, 3H). ^13^C NMR (101 MHz, CD_3_OD) δ 179.0, 158.8, 137.7, 130.5 (2C), 130.4 (2C), 129.6, 128.0, 54.2, 46.0, 45.0, 40.9, 38.7, 26.8, 26.7 (2C), 25.4, 23.8, 22.0. The LCMS-ESI *m/z* was 388.2 [M − H]^+^.

### Compound 46.

Compound 46 was ((*R*)-1-((*S*)-2-acetamido-3-phenylpropanamido)-2-phenylethyl)boronic acid. It is a white solid (yield, 14%). ^1^H NMR (400 MHz, CD_3_OD) δ 7.30 to 6.98 (m, 8H), 6.85 (d, *J* = 7.0 Hz, 2H), 4.62 (t, *J* = 7.9 Hz, 1H), 3.08 to 2.84 (m, 2H), 2.77 to 2.59 (m, 2H), 2.20 (dd, *J* = 13.9, 10.1 Hz, 1H), 1.81 (s, 3H). ^13^C NMR (101 MHz, CD_3_OD) δ 177.8, 173.2, 142.0, 137.2, 130.5, 130.2 (2C), 129.9 (2C), 129.8 (2C), 129.4 (2C), 129.3 (2C), 128.3, 127.0, 52.7, 38.6, 38.0, 22.2. The LCMS-ESI *m/z* was 353.0 [M − H]^+^.

### Compound 47.

Compound 47 was ((*R*)-1-((*S*)-2-(benzo[*d*]thiazole-7-carboxamido)-3-phenylpropanamido)-3-cyclohexylpropyl)boronic acid. It is a white solid (yield, 23%). ^1^H NMR (400 MHz, CD_3_OD) δ 9.31 (s, 1H), 8.25 (dd, *J* = 8.1, 0.9 Hz, 1H), 8.08 (d, *J* = 7.5 Hz, 1H), 7.68 (dd *J* = 7.8 Hz, 1H), 7.38 to 7.18 (m, 5H), 5.07 to 4.95 (m, 1H), 3.30 to 3.24 (m, 2H), 2.52 (dd, *J* = 8.7, 6.0 Hz, 1H), 1.23 to 1.64 (m, 4H), 1.57 to 0.73 (m, 11H). ^13^C NMR (101 MHz, CD_3_OD) δ 177.3, 167.9, 160.9, 155.2, 137.3, 134.3, 130.5 (2C), 129.7 (2C), 128.5, 128.2, 127.5, 127.3, 125.3, 53.7, 47.4, 39.1, 38.3, 36.6, 34.5, 34.5, 29.1, 27.8, 27.5 (2C). The LCMS-ESI *m/z* was 492.1 [M − H]^+^.

### Compound 48.

Compound 48 was ((*R*)-1-((*S*)-2-benzamido-3-phenylpropanamido)-3-cyclohexylpropyl)boronic acid. It is a white solid (yield, 43%). ^1^H NMR (400 MHz, CD_3_OD) δ 7.85 to 7.65 (m, 2H), 7.59 to 7.41 (m, 3H), 7.36 to 7.11 (m, 5H), 4.97 (t, *J* = 8 Hz, 1H), 3.28 to 3.13 (m, 2H), 2.49 (dd, *J* = 8.6, 6.1 Hz, 1H), 1.75 to 1.60 (m, 5H), 1.50 to 0.64 (m, 11H). ^13^C NMR (101 MHz, CD_3_OD) δ 177.5, 170.3, 137.4, 134.9, 133.0, 130.5 (2C), 130.3, 129.7 (2C), 129.5 (2C), 128.5 (2C), 128.2, 53.4, 47.5, 39.1, 38.4, 36.6, 34.5, 34.5, 29.1, 27.8, 27.5. The LCMS-ESI *m/z* was 435.2 [M − H]^+^.

### Compound 49.

Compound 49 was ((*R*)-3-cyclohexyl-1-((*S*)-2-(2-fluorobenzamido)-3-phenylpropanamido)propyl)boronic acid. It is a white solid (yield, 18%). ^1^H NMR (400 MHz, CD_3_OD) δ 7.68 to 7.62 (m, 1H), 7.56 to 7.47 (m, 1H), 7.35 to 7.16 (m, 7H), 5.05 to 4.92 (m, 1H), 3.19 (dd, *J* = 7.8, 3.0 Hz, 2H), 2.51 (dd, *J* = 8.7, 6.0 Hz, 1H), 1.75 to 1.61 (m, 4H), 1.53 to 1.35 (m, 1H), 1.35 to 0.71 (m, 10H). ^13^C NMR (101 MHz, CD_3_OD) δ 177.1, 166.6, 161.5 (d, *J* = 250.2 Hz), 137.1, 134.5 (d, *J* = 8.8 Hz), 131.5 (d, *J* = 2.3 Hz), 130.5, 130.4 (2C), 129.7 (2C), 128.3, 125.6 (d, *J* = 3.5 Hz), 123.5 (d, *J* = 13.5 Hz), 117.2 (d, *J* = 22.9 Hz), 53.4, 47.3, 39.1, 38.6, 36.5, 34.5, 34.5, 29.1, 27.8, 27.5 (2C). The LCMS-ESI *m/z* was 453.2 [M − H]^+^.

### Compound 50.

Compound 50 was ((*R*)-3-cyclohexyl-1-((*S*)-2-(2-methyl-2-(1*H*-pyrrol-1-yl)propanamido)-3-phenylpropanamido)propyl)boronic acid. It is a white solid (yield, 45%). ^1^H NMR (400 MHz, CD_3_OD) δ 7.36 to 7.17 (m, 3H), 7.17 to 7.10 (m, 2H), 6.78 (t, *J* = 2.2 Hz, 2H), 6.53 (d, *J* = 7.5 Hz, 2H), 6.17 (t, *J* = 2.2 Hz, 2H), 4.74 to 4.69 (m, 1H), 3.0 (dd, *J* = 13.7, 7.0 Hz, 2H), 2.91 (dd, *J* = 13.7, 8.1 Hz, 1H), 2.50 (dd, *J* = 8.6, 6.1 Hz, 1H), 1.75 to 1.65 (m, 3H), 1.69 (s, 3H), 1.62 (s, 3H), 1.53 to 1.36 (m, 1H), 1.34 to 1.03 (m, 8H), 0.97 to 0.79 (m, 2H). ^13^C NMR (101 MHz, CD_3_OD) δ 176.7, 176.6, 136.7, 130.5 (2C), 129.7 (2C), 128.3, 120.0 (2C), 110.2 (2C), 63.1, 52.7, 46.9, 39.1, 38.5, 36.5, 34.6, 34.5, 29.1, 27.8, 27.5, 26.7 (2C), 26.7. The LCMS-ESI *m/z* was 466.2 [M − H]^+^.

### Compound 51.

Compound 51 was ((*R*)-1-((*S*)-2-benzamido-3-phenylpropanamido)-3-phenylpropyl)boronic acid. It is a white solid (yield, 30%). ^1^H NMR (400 MHz, CD_3_OD) δ 7.71 to 7.12 (m, 2H), 7.45 to 7.39 (m, 1H), 7.36 to 7.29 (m, 2H), 7.24 to 7.16 (m, 4H), 7.15 to 6.98 (m, 6H), 4.89 (t, *J* = 8.0 Hz, 1H), 3.18 to 3.06 (m, 2H), 2.48 (dd, *J* = 8.5, 6.1 Hz, 1H), 2.43 to 2.30 (m, 2H), 1.65 to 1.55 (m, 1H), 1.47 to 1.31 (m, 1H). ^13^C NMR (101 MHz, CD_3_OD) δ 177.7, 170.4, 143.5, 137.5, 134.9, 133.2, 130.9, 130.5 (2C), 129.7 (2C), 129.5 (2C), 129.4 (2C), 129.2 (2C), 128.5 (2C), 128.3, 128.5, 128.6, 126.7, 53.4, 46.4, 38.3, 34.9, 33.9. The LCMS-ESI *m/z* was 429.1 [M − H]^+^. The HRMS-ESI *m/z* [M − H]^+^ were 429.1996 (calculated for C_25_H_26_BN_2_O_4_) and 429.1989 (found).

### Compound 52.

Compound 52 was ((*R*)-3-phenyl-1-((*S*)-3-phenyl-2-(4-(trifluoromethyl)benzamido)propanamido)propyl)boronic acid. It is a white solid (yield, 20%). ^1^H NMR (400 MHz, CD_3_OD) δ 7.94 (d, *J* = 8.1 Hz, 2H), 7.77 (d, *J* = 8.2 Hz, 2H), 7.36 to 7.28 (m, 4H), 7.27 to 7.20 (m, 3H), 7.15 to 7.05 (m, 3H), 5.00 (t, *J* = 8.0 Hz, 1H), 3.31 to 3.15 (m, 2H), 2.59 (dd, *J* = 8.5, 6.2 Hz, 1H), 2.52 to 2.45 (m, 2H), 1.77 to 1.64 (m, 1H), 1.56 to 1.42 (m, 1H). ^13^C NMR (101 MHz, CD_3_OD) δ 177.5, 168.9, 143.6, 138.6, 137.3, 134.4 (q, *J* = 32.5 Hz), 130.5 (2C), 129.7 (2C), 129.4 (2C), 129.3 (2C), 129.2 (2C), 128.3, 126.7, 126.5 (q, *J* = 3.8 Hz), 53.6, 46.4, 38.3, 34.9, 33.9. The LCMS-ESI *m/z* was 497.1 [M − H]^+^. The HRMS-ESI *m/z* [M − H]^+^ were 497.1870 (calculated for C_26_H_25_BF_3_N_2_O_4_) and 497.1875 (found).

### Compound 53.

Compound 53 was ((*R*)-1-((*S*)-2-(2-fluorobenzamido)-3-phenylpropanamido)-3-phenylpropyl)boronic acid. It is a white solid (yield, 25%). ^1^H NMR (400 MHz, CD_3_OD) δ 7.65 (td, *J* = 7.5, 1.7 Hz, 1H), 7.56 to 7.48 (m, 1H), 7.35 to 7.28 (m, 4H), 7.28 to 7.18 (m, 5H), 7.18 to 7.09 (m, 3H), 5.0 (t, *J* = 7.8 Hz, 1H), 3.21 (dd, *J* = 7.8, 3.3 Hz, 2H), 2.60 (dd, *J* = 8.6, 6.0 Hz, 1H), 2.452 to 2.41 (m, 2H), 1.78 to 1.66 (m, 1H), 1.58 to 1.41 (m, 1H). ^13^C NMR (101 MHz, CD_3_OD) δ 177.3, 166.7, 161.5 (d, *J* = 250.2 Hz), 143.6, 137.0, 134.5 (d, *J* = 8.8 Hz), 130.5 (2C), 131.5 (d, *J* = 2.3 Hz), 129.8 (2C), 125.6 (d, *J* = 3.5 Hz), 129.4 (2C), 129.2 (2C), 128.3, 126.7, 123.5 (d, *J* = 13.5 Hz), 117.2 (d, *J* = 22.9 Hz), 53.4, 49.0, 46.1, 38.5, 34.9, 33.9. The LCMS-ESI *m/z* was 477.1 [M − H]^+^. The HRMS-ESI *m/z* [M − H]^+^ were 447.1901 (calculated for C_25_H_25_BFN_2_O_4_) and 447.1908 (found).

### Compound 54.

Compound 54 was ((*R*)-1-((*S*)-2-(2-methyl-2-(1*H*-pyrrol-1-yl)propanamido)-3-phenylpropanamido)-3-phenylpropyl)boronic acid. It is a white solid (yield, 50%). ^1^H NMR (400 MHz, CD_3_OD) δ 7.59 to 6.99 (m, 1H), 6.79 (t, *J* = 2.1 Hz, 1H), 6.17 (t, *J* = 2.1 Hz, 1H), 4.79 to 4.70 (m, 1H), 3.01 (dd, *J* = 13.7, 7.1 Hz, 1H), 2.92 (dd, *J* = 13.7, 8.2 Hz, 1H), 2.59 (dd, *J* = 8.4, 6.3 Hz, 1H), 2.56 to 2.43 (m, 1H), 1.78 to 1.66 (m, 1H), 1.69 (s, 1H), 1.62 (s, 1H), 1.59 to 1.43 (m, 1H). ^13^C NMR (101 MHz, CD_3_OD) δ 176.9, 176.6, 143.5, 136.7, 130.5, 129.8, 129.4, 129.3, 128.3, 126.7, 120.0, 119.9, 119.9, 110.2, 110.1, 110.0, 63.1, 52.8, 45.0, 38.3, 34.1, 33.5, 26.2, 26.7. The LCMS-ESI *m/z* was 460.2 [M − H]^+^. The HRMS-ESI *m/z* [M − H]^+^ were 460.2418 (calculated for C_26_H_31_BN_3_O_4_) and 460.2420 (found).

### Compound 55.

Compound 55 was ((*R*)-3-phenyl-1-((*S*)-3-phenyl-2-(2-phenylpyrimidine-5-carboxamido)propanamido)propyl)boronic acid. It is a white solid (yield, 43%). ^1^H NMR (400 MHz, CD_3_OD) δ 9.13 to 9.01 (m, 2H), 8.49 to 8.43 (m, 2H), 7.57 to 7.07 (m, 15H), 5.03 (t, *J* = 8.0 Hz, 1H), 3.28 to 3.16 (m, 2H), 2.65 to 2.59 (m, 1H), 2.62 (t, *J* = 7.4 Hz, 1H), 2.49 (t, *J* = 7.0 Hz, 2H), 1.78 to 1.65 (m, 1H), 1.58 to 1.44 (m, 1H). ^13^C NMR (101 MHz, CD_3_OD) δ 177.3, 167.5, 166.3, 157.8, 143.6, 137.9, 137.2, 132.8, 130.5 (2C), 130.35, 129.8 (2C), 129.7 (4C), 129.4 (2C), 129.3 (2C), 128.4, 126.70, 126.31, 53.4, 46.2, 38.3, 34.9, 33.9. The LCMS-ESI *m/z* was 507.1 [M − H]^+^.

### Compound 56.

Compound 56 was ((*R*)-3-methyl-1-((*S*)-2-(pyrazine-2-carboxamido)propanamido)butyl)boronic acid. It is a white solid (yield, 20%). ^1^H NMR (400 MHz, CD_3_OD) δ 9.23 (s, 1H), 8.81 (d, *J* = 2.4 Hz, 1H), 8.71 (s, 1H), 4.86 (q, *J* = 7.2 Hz, 1H), 2.76 (t, *J* = 7.6 Hz, 1H), 1.65 (dt, *J* = 13.4, 6.7 Hz, 1H), 1.57 (d, *J* = 7.2 Hz, 1H), 1.35 (t, *J* = 7.3 Hz, 2H), 0.91 (d, *J* = 6.6 Hz, 1H). ^13^C NMR (101 MHz, CD_3_OD) δ 178.6, 165.3, 148.8, 145.9, 144.9, 144.8, 47.4, 44.5, 40.9, 27.0, 23.6, 22.6, 17.7. The LCMS-ESI *m/z* was 307.1 [M − H]^+^.

### Compound 57.

Compound 57 was ((*R*)-1-((*S*)-3-(4-hydroxyphenyl)-2-(pyrimidine-4-carboxamido)propanamido)-3-phenylpropyl)boronic acid. It is a white solid (yield, 34%). ^1^H NMR (400 MHz, CD_3_OD) δ 9.19 (s, 1H), 8.79 (s, 1H), 8.69 (s, 1H), 7.26 to 7.05 (m, 8H), 6.72 (d, *J* = 8.5 Hz, 2H), 4.98 (t, *J* = 7.5 Hz, 1H), 3.24 to 3.10 (m, 2H), 2.61 (dd, *J* = 8.1, 6.5 Hz, 1H), 2.57 to 2.44 (m, 2H), 1.79 to 1.68 (m, 1H), 1.63 to 1.50 (m, 1H). ^13^C NMR (101 MHz, CD_3_OD) δ 177.2, 165.2, 157.8, 148.9, 145.6, 144.8, 143.6, 131.6 (2C), 129.5 (2C), 129.3 (2C), 127.5, 126.6, 116.5 (2C), 53.2, 44.5, 37.9, 35.0, 34.0. The LCMS-ESI *m/z* was 477.1 [M − H]^+^. The HRMS-ESI *m/z* [M − H]^+^ were 447.1849 (calculated for C_23_H_24_BN_4_O_5_) and 447.1843 (found).

### Compound 58.

Compound 58 was ((*R*)-3-phenyl-1-((*S*)-2-(pyrazine-2-carboxamido)-3-(pyridin-2-yl)propanamido)propyl)boronic acid. It is a pale-pink solid (yield, 23%). ^1^H NMR (400 MHz, CD_3_OD) δ 9.17 (s, 1H), 8.80 (d, *J* = 1.4 Hz, 1H), 8.73 to 8.68 (m, 2H), 8.34 (t, *J* = 7.9 Hz, 1H), 7.90 (d, *J* = 7.9 Hz, 1H), 7.80 (t, *J* = 6.6 Hz, 1H), 7.80 (t, *J* = 6.6 Hz, 1H), 7.27 to 7.05 (m, 5H), 5.32 (t, *J* = 7.0 Hz, 1H), 3.77 (dd, *J* = 14.6, 6.0 Hz, 1H), 3.58 (dd, *J* = 14.5, 8.5 Hz, 1H), 2.79 (d, *J* = 6.8 Hz, 1H), 2.60 (t, *J* = 7.9 Hz, 2H), 1.88 to 1.64 (m, 2H). ^13^C NMR (101 MHz, CD_3_OD) δ 165.5, 154.8, 149.0, 145.5, 144.9, 144.8, 144.5, 143.5, 129.4 (2C), 129.3 (2C), 128.8, 126.8, 126.2, 51.5, 44.1, 49.0, 37.2, 34.5, 33.7. The LCMS-ESI *m/z* was 432.1 [M − H]^+^. The HRMS-ESI *m/z* [M − H]^+^ were 432.1852 (calculated for C_22_H_23_BN_5_O_4_) and 432.1848 (found).

### Molecular modeling.

The M. tuberculosis ClpP1P2 X-ray structure (Protein Data Bank [PDB] accession number 4U0G) (29), human-proteasome X-ray structure (accession numbers 4R3O and 4R67) (30), and yeast-proteasome X-ray structure (accession number 5CZ7) (31) were downloaded from the Protein Data Bank (http://www.rcsb.org) and prepared using the protein preparation wizard in Maestro release 2016-1 (Maestro, MacroModel, Schrödinger, LLC) using standard settings. This included the addition of hydrogen atoms, bond assignments, removal of water molecules further than 5 Å from the ligand, protonation state assignment, and optimization of the hydrogen bond network. The proteasome structures were superimposed using structural alignment, and the cocrystallized inhibitor from the yeast structure was merged into the human-proteasome structure and covalently attached to the catalytic serines. Root mean square deviations (RMSD) of the inhibitor heavy atoms differ by 0.43 Å from those of the human-proteasome structure with bortezomib (PDB accession number 5LF3), which was published after this work was carried out (33). In the M. tuberculosis ClpP1P2 structure, the cocrystallized ligand was replaced with the bioactive conformation of compound 1 from the X-ray structure of the Lon-like protease MtaLonC (PDB accession number 4FWD) (34). This was done by manually positioning compound 1 so that a covalent bond could be formed between the boronic acid and the catalytic serine. All of the backbone donors and acceptors of compound 1 formed hydrogen bonds with ClpP1P2 and the proteasome. The final ClpP1P2 and human-proteasome–compound 1 complex was then subjected to 200 steps of Polak-Ribiere conjugate gradient (PRCG) minimization using MacroModel release 2016-1, the OPLS3 force field (35), and the generalized Born/hydrophobic solvent accessible surface area (GB/SA) solvation model (36). Compound 1 is expected to be neutral at physiological pH, but the proximity of the base in the catalytic triad may cause one of the boronate hydroxy groups to be deprotonated. Both neutral and deprotonated forms were modeled, and no significant difference was found between the minimized complexes. Further modeling of the human proteasome was done using the chymotrypsin-like proteolytic site where compound 1 is bound to the threonine of subunit beta type 5 (PDB chain L), with one of the boronate hydroxyls deprotonated. Further modeling of the ClpP1P2 was done using the binding site where compound 1 is bound to the serine of proteolytic subunit 1 (chain I), with one of the boronate hydroxy compounds deprotonated. Residue numbering was taken from the UniProt entries (accession number P9WPC5 for ClpP1 and accession numbers P28074 and P20618 for human-proteasome units β5 and β1, respectively; http://www.uniprot.org) and is not necessarily consistent with the PDB residue numbers.

The remaining inhibitors were manually modeled into ClpP1P2 and the human proteasome by mutating the P1, P2, or CAP of compound 1 in the above-described complex. The final structure was then subjected to 200 steps of PRCG minimization using the OPLS3 force field (35) and GB/SA solvation model (36), while atoms more than 9 Å from the inhibitor were constrained.

### Biology. (i) Bacterial strains, mammalian cells, and culture conditions.

M. smegmatis mc^2^ 155 (ATCC 700084), M. bovis BCG (ATCC 35734), and M. tuberculosis H37Rv (ATCC 27294) wild-type strains and derived M. smegmatis Δ*prcAB*-mRFP-SsrA reporter strains were maintained in Middlebrook 7H9 medium (Difco) supplemented with 0.5% (vol/vol) glycerol, 0.05% (vol/vol) Tween 80, and 10% (vol/vol) Middlebrook ADC (albumin-dextrose-catalase) (Difco). When appropriate, hygromycin B (Roche), kanamycin (Sigma-Aldrich), and/or streptomycin (Sigma-Aldrich) was added. Enumeration of bacteria was performed by plating them on Middlebrook 7H10 (Difco) agar plates containing 0.5% (vol/vol) glycerol and 10% (vol/vol) Middlebrook OADC (oleic acid-albumin-dextrose-catalase) (Difco). HepG2 cells (HB-8065) were grown in Dulbecco's modified Eagle's medium (DMEM) and cultured at 37°C in a 5% CO_2_ atmosphere and DMEM (Gibco) complemented with 10% heat-inactivated fetal bovine serum (FBS; Gibco), penicillin (100 U/ml; Gibco), and streptomycin (100 μg/ml; Gibco).

### (ii) M. smegmatis Δ*prcAB* proteasome knockout strain.

In order to obtain an M. smegmatis mc^2^ 155 *prcAB* null mutant, i.e., a strain in which the two genes encoding the two mycobacterial proteasome subunits (*prcA* and *prcB*) were deleted, we performed recombineering as previously described (22). A double-stranded 1,000-bp allelic-exchange substrate (AES) was constructed by stitch-PCR linking a fragment of 500 bp with homology to the region upstream of the *prcA* gene to another 500-bp fragment with homology to the region downstream of the *prcB* gene using flanking primer sets (see Table S1 in the supplemental material).

Electrocompetent cells of M. smegmatis containing plasmid pJV53 (Kan^r^) were mixed with 200 ng of the AES and 100 ng of cotransforming plasmid pTCS-mcs (Strep^r^). pJV53 was a gift from Graham F. Hatfull. pTCS-mcs was a gift from Dirk Schnappinger (Addgene plasmid 31288). Cotransformation with pTCS-mcs, carrying a streptomycin resistance cassette, was used to select the electrocompetent subpopulation of bacteria. Transformed cells were recovered by shaking them for 4 h at 37°C in 7H9 medium supplemented with 10% ADC and 0.05% Tween 80 and then plated on 7H10 agar supplemented with 10% oleic acid-albumin-catalase, 25 μg/ml streptomycin, and 25 g/ml kanamycin. One hundred streptomycin-resistant transformants were streaked on 7H10 agar and tested by colony PCR. Colony PCR was performed by resuspending the cells in 200 μl H_2_O and boiling them at 95°C for 20 min, followed by immediate cooling on ice, and an aliquot comprising 1/10 of the volume of the mixture was used as a template for PCR. Recombinants, i.e., colonies in which the *prcAB* genes were deleted, were detected using a pair of forward/reverse flanking primers and a pair of forward/reverse internal/flanking primers (Table S1). PCR parameters used for detection of recombinants were as follows: *Taq* DNA polymerase, with an initial denaturation at 95°C for 3 min, followed by cycle denaturation at 95°C for 30 s, annealing at 55°C for 30 s, extension at 72°C for 30 s for 25 cycles, and a final extension at 72°C for 5 min. Detected recombinants were restreaked several times on nonselective 7H10 agar plates in order to remove recombineering plasmid pJV53 (Kan^r^). Targeted deletion of the *prcAB* genes was confirmed by discriminatory PCR and sequencing using primers flanking or specific to the *prcAB* locus (Table S1). Amplicons were detected and visualized on agarose gel stained with Sybr-safe (Promega).

### (iii) M. smegmatis Δ*prcAB*-mRFP-SsrA and ClpP1P2 inhibition assay.

The plasmid pGMEH-p38-mRFP-SsrAec3 (Hygro^r^) carries the mCherry RFP gene fused to the ClpP1P2-specific SsrA tag cloned downstream of the p38 strong mycobacterial promoter and an hygromycin resistance cassette. pGMEH-P38-che-ssrAec3 was a gift from Dirk Schnappinger (Addgene plasmid 27059). The plasmid was electroporated into M. smegmatis Δ*prcAB* to generate M. smegmatis Δ*prcAB*-mRFP-SsrA. Transformants were recovered on 7H10 agar supplemented with 25 μg/ml streptomycin and 50 μg/ml hygromycin and grown in 7H9 broth supplemented with the same concentrations of streptomycin and hygromycin. Precultures were then harvested at mid-log phase, diluted to an OD_600_ of 0.2 in complete 7H9 medium, and dispensed into 96-well plates (200 μl/well) in the presence of 2-fold serially diluted compounds. M. smegmatis p38-mRFP-SsrA alone was used as a negative control, whereas M smegmatis p38-mRFP-SsrA treated with compound 1 was used as a positive control. Fluorescence signal acquisition was determined after 3 h of incubation using an M200 Pro plate reader (Tecan). Red fluorescence was acquired under excitation/emission at wavelengths of 587/630 nm. Relative fluorescence was plotted as a function of drug concentration. The maximum fluorescence value obtained with compound 1 was taken as the maximum (i.e., 100%) of ClpP1P2 inhibition. The IC_50_ for ClpP1P2 (i.e., the concentration required to inhibit 50% of ClpP1P2's activity) was determined in three independent replicates.

### MIC determination.

Turbidity-based growth inhibition was performed to assess the antimycobacterial potencies of the synthesized compounds. M. smegmatis Δ*prcAB* or M. tuberculosis H37Rv strain precultures were harvested at mid-log phase and diluted to an OD_600_ of 0.05 in complete 7H9 medium. Bacterial suspensions were then dispensed in 96-well plates (200 μl/well, M. smegmatis) in the presence of a twofold serial dilution of compound tested ranging from 100 to 0.2 µM and incubated for 24 h (M. smegmatis) or 5 days (M. tuberculosis) at 37°C with shaking (100 rpm). Cells were manually resuspended, and their OD was measured at 600 nm on the M200Pro plate reader (Tecan). The percentage of growth was determined relative to that of an untreated control and plotted as a function of drug concentration. The MIC_50_ was determined in three independent replicates.

For experiments with additional serum, 10% deactivated fetal bovine serum (Gibco) was added to complete 7H9 medium to assess the serum's effect on the MIC where indicated in the tables. Bacterial suspensions were then processed as described above.

### Mammalian proteasome CT-like peptidase inhibition assay.

A cell-based CT-like peptidase assay was performed using the Proteasome-Glo cell-based assay reagent (Promega) according to the manufacturer's guidelines. Briefly, HepG2 cells (10^4^ cells/well) were treated with the compounds indicated in the figures for 2 h, followed by incubation with the luminescent substrate for 10 min. Luminescence was detected with a Tecan M200 Pro plate reader. Relative luminescence units (RLU) were plotted as a function of drug concentration, and the IC_50_ for the proteasome (i.e., the concentration required to inhibit 50% of the proteasome's activity) was determined. Compound 1 was used as a positive control.

### Protease panel.

Protease panel testing was carried out by Reaction Biology Corporation.

### *In vitro* ADME.

A parallel artificial-membrane permeability assay (PAMPA) and mouse liver microsome (MLM), human liver microsome (HLM), aqueous solubility, Log D, protein binding, and cytochrome P450 2D6 and 3A4 assays were carried out at Piramal Pharma Solutions.

### Cytotoxicity assays.

The MTS (Promega) reduction assay was used according to manufacturer's guidelines to assess Vero (ATCC CCL-8) and HepG2 (ATCC HB-8065) cell viability. After 20,000 cells were exposed to compounds, absorbance was read with a Tecan M200 Pro plate reader at 570 nm. Optical density was plotted as a function of drug concentration, and cytocidal concentrations were determined. Carbonyl cyanide *m*-chlorophenylhydrazone (CCCP) was used as a cytotoxic positive control. Cytotoxicity was determined twice in independent experiments.

### Pharmacokinetics.

Female ICR mice (aged ∼6 to ∼8 weeks; 3 animals per time point) were used for all studies. Studies were performed as per approved internal protocols for animal care and use. Doses were administered as clear solutions in 50% polyethylene glycol 400 (PEG400) plus 50% dextrose and 5% water (D5W). Animals were sacrificed by overdose of CO_2_, and blood was collected through cardiac puncture at 5 min (10 min *per os* [p.o.]), 30 min, and 1, 2, 4, 8, 16, and 24 h after administration in tubes containing K3 EDTA as an anticoagulant. The samples were centrifuged, and the plasma was separated and stored at −70°C until analysis. Plasma samples were processed and analyzed by LC-tandem MS. Pharmacokinetic parameters were estimated by noncompartmental methods using WinNonlin (version 5.2; Pharsight, CA).

## Supplementary Material

Supplemental material
