# Supplementary material for: Towards Selective Mycobacterial ClpP1P2 Inhibitors with Reduced Activity against the Human Proteasome
Source: Antimicrob Agents Chemother. 2017 Apr 24;61(5):e02307-16. doi: 10.1128/AAC.02307-16 (PMC5404560; doi:10.1128/AAC.02307-16)
Supplement: Supplemental material [file supp_61_5_e02307-16__index.html]

Towards Selective Mycobacterial ClpP1P2 Inhibitors with Reduced Activity against the Human Proteasome — Supplemental material 

# Towards Selective Mycobacterial ClpP1P2 Inhibitors with Reduced Activity against the Human Proteasome

## Supplemental material

- Supplemental file 1 -

  Supplemental material

  PDF, 762K
