# Supplementary material for: Towards Selective Mycobacterial ClpP1P2 Inhibitors with Reduced Activity against the Human Proteasome
Source: Antimicrob Agents Chemother. 2017 Apr 24;61(5):e02307-16. doi: 10.1128/AAC.02307-16 (PMC5404560; doi:10.1128/AAC.02307-16)
Supplement: Supplemental material [file AAC.02307-16_zac005176106s1.pdf]

Supporting Information

Towards Selective Mycobacterial ClpP1P2 Inhibitors with Reduced Activity Against the Human Proteasome

Wilfried Moreira,[‡,a] Sridhar Santhanakrishnan,[‡,a,b] Grace J. Y. Ngan,[a] Choon Bing Low,[c] Kanda Sangthongpitag,[c] Anders Poulsen,[c,d] Brian W. Dymock,[b]# Thomas Dick[a]

[a]Department of Microbiology and Immunology, Yong Loo Lin School of Medicine, National University of Singapore, Singapore

[b]Department of Pharmacy, National University of Singapore, Singapore

[c]Experimental Therapeutics Center, Agency for Science, Technology and Research (A*STAR), Singapore

[d]Department of Chemistry, National University of Singapore, 3 Science Drive 3, Singapore 117543

[‡]These authors contributed equally.

#Address correspondence to Brian W. Dymock, phadbw@nus.edu.sg.

Table of Contents

1. **Synthesis of (*S*)-3-phenyl-2-(pyrazine-2-carboxamido)propanoic aacid (6) 3**
2. **Synthesis of CAP precursors 7(c-i), 7k 4**
3. **Synthesis of amine esters 8(b-d) 16**
4. **Synthesis of CAP esters 9(a-o) 17**
5. **Synthesis of CAP carboxylic acids 10(a-o) 20**
6. **Synthesis of aldimines 13(a-g) 27**
7. **Synthesis of amino boronate salts 16(a-g) 31**
8. **Synthesis of diamides (18a-ff/19/21) and compound 22 ....32**
9. **NMR spectra of compound 58 ....33**
10. **Protease Panel Testing of compound 58 ....35**

1. Synthesis of (*S*)-3-phenyl-2-(pyrazine-2-carboxamido)propanoic aacid (6)

Reaction 1: Under nitrogen, *N*, *O*-bis(trimethylsilyl)acetamide (BSA) (7.40 mL, 30.30 mmol, 2.0 eq) was added to a stirred solution of *L*-phenylalanine **2** (2.5 g, 15.15 mmol, 1.0 eq) in dry CH_2Cl_2 (50 mL) at room temperature and allowed to stir for 16 h at same temperature to get *N*,*O*-bis(trimethylsilyl)-*L*-phenylalanine solution **3**.

Reaction 2: *N N'*-carbonyldiimidazole (4.91 g, 30.30 mmol, 2.0 eq) was added to the stirred suspension of pyrazine-2-carboxylic acid **4** (2.81 g, 22.70 mmol, 1.5 eq) in dry CH_2Cl_2 (50 mL) at room temperature under nitrogen. The reaction mixture was allowed to stir for 16 h to yield pyrazinecarboxylic acid imidazolide solution **5**. Then the reaction mixture was cooled to -40 ^{o}C, and then a solution of *N*,*O*-Bis(trimethylsilyl)-*L*-phenylalanine **3** was added drop wise during 30 min. The reaction mixture slowly warm to room temperature during 2 h and stirring was continued for 16 h at room temperature. To the reaction mixture a solution of citric acid (5 g in 50 mL of water) was added and stirred for 10 min. The aqueous phase was separated and extracted with CH_2Cl_2 (2×50 mL). The combined organic layers were diluted with ether (100 mL) and dried over Na_2SO_4. The solvent was removed under vacuum at 35 ^{o}C to give (pyrazine-2-carbonyl)-*L*-phenylalanine (**6**) as off white solid (61% yield). ^{1}H NMR (400 MHz, DMSO-d6) δ 9.13 (s, 1H), 8.87-8.86 (m, 1H), 8.82 (d, *J* = 8.0 Hz, 1H), 8.73 (d, *J* = 3.6 Hz, 1H), 7.23-7.16 (m, 5H), 4.77-4.71 (m, 1H), 3.22-3.20 (m, 2H); ^{13}C NMR (400 MHz, DMSO-d6) δ 172.3, 162.5, 147.7, 144.0, 143.4, 143.3, 137.3, 129.0, 128.1, 126.4, 53.4, 36.2; LCMS-ESI (*m/z*): 272.1 $[M + H]^+$.

2. Synthesis of CAP precursors 7(c-i), 7k

2-phenylpyrimidine-5-carboxylic acid (7c):

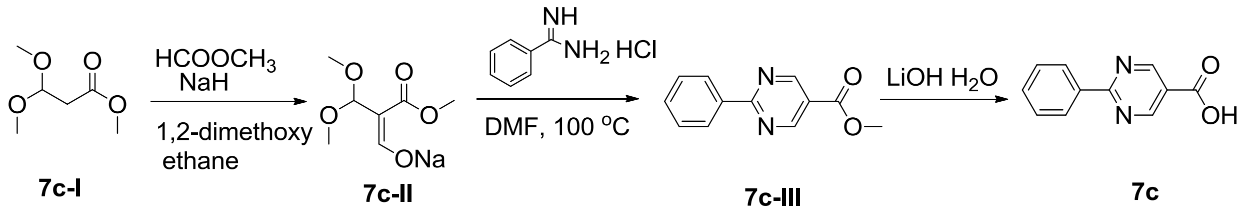

Step 1: Synthesis of sodium (*Z*)-2-(dimethoxymethyl)-3-methoxy-3-oxoprop-1-en-1-olate (7c-II)

Methyl formate (1.86 g, 31.04 mmol, 2.3 eq) was added to a solution of methyl 3,3-dimethoxypropanoate (**7c-I**) (2.0 g, 13.50 mmol, 1 eq) in 1,2 dimethoxy ethane (15 mL). The reaction mixture was cooled to 0 °C and NaH (0.8 g, 20.25 mmol, 1.5 eq) was added portion wise. The reaction mixture was allowed to stir at 40 °C for 10 min., cooled to 0 °C, stirred for 1 h, and then the reaction mixture was allowed to stir at room temperature for 12 h. Then diethyl ether (20 mL) was added to the reaction mixture, filtered the solid, dried under vacuum and co-distilled with toluene to yield the sodium (*Z*)-2-(dimethoxymethyl)-3-methoxy-3-oxoprop-1-en-1-olate (**7c-II**) as off-white solid.

Step 2: Synthesis of methyl 2-phenylpyrimidine-5-carboxylate (7c-III)

Under nitrogen, benzimidine hydrochloride (1.51 g, 7.66 mmol, 1.2 eq) was added to the sodium (*Z*)-2-(dimethoxymethyl)-3-methoxy-3-oxoprop-1-en-1-olate (**7c-II**) (1.0 g, 6.38 mmol, 1.0 eq) in dry DMF (12 mL) at room temperature. The reaction mixture was allowed to stir at 100 °C for 3 h under N_2 atmosphere. Ice-cold water (20 mL) was added to the reaction mixture. The resulting solids were filtered, dried under vacuum to yield desired product methyl 2-phenylpyrimidine-5-carboxylate (**7c-III**) as off-white solid (20%). ^{1}H NMR (400 MHz, DMSO-d6) δ 9.32 (s, 2H), 8.47 (t, *J*= 6.0 Hz, 2H), 7.63-7.56 (m, 3H), 3.93 (s, 3H); LCMS-ESI (*m/z*): 215.1 [M + H]+.

Step 3: Synthesis of 2-phenylpyrimidine-5-carboxylic acid (7c)

Lithium hydroxide monohydrate (0.147 g, 3.50 mmol, 3.0 eq) was added to a solution of methyl 2-phenylpyrimidine-5-carboxylate (**7c-III**) (0.25 g, 1.16 mmol, 1.0 eq) in THF (8 mL), methanol (8 mL) and water (3 mL). The reaction mixture was allowed to stir at room temperature for 3 h. The reaction mixture was concentrated under vacuum. The residue was taken in water (5 mL), and pH was adjusted to 3 using 1N HCl. The solids were filtered, washed with water and dried under vacuum to yield 2-phenylpyrimidine-5-carboxylic acid (**7c**) as off-white solid (85% yield). ^{1}H NMR (400 MHz, DMSO-d6) δ 13.73 (bs, 1H), 8.47 (d, *J*= 6.8 Hz, 2H), 7.60-7.53 (m, 3H); LCMS-ESI (*m/z*): 201.0 [M + H]+.

Synthesis of 6-phenylpyrimidine-4-carboxylic acid (7d)

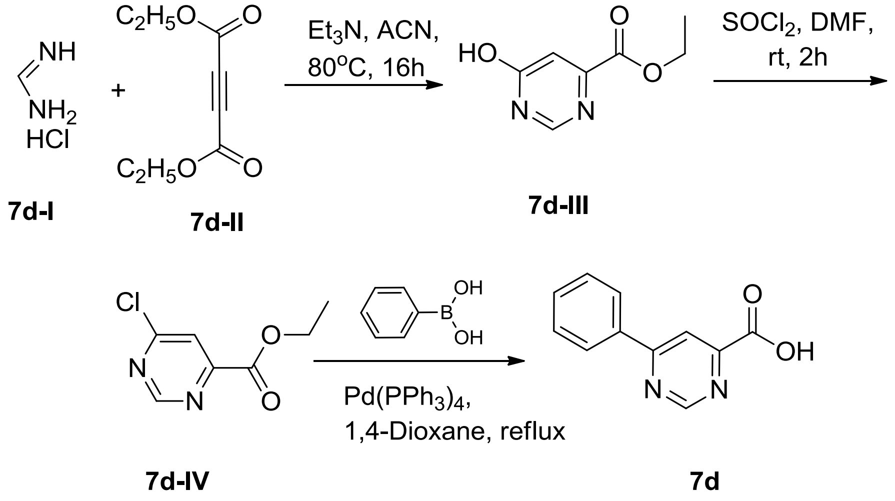

Step 1: Synthesis of ethyl 6-hydroxypyrimidine-4-carboxylate (7d-III)

Formamidine hydrochloride (**7d-I**) (1.0 g, 12.49 mmol, 1.0 eq) was added to a stirred solution of diethyl but-2-ynedioate (**7d-II**) (2.12 g, 12.49 mmol, 1.0 eq) in acetonitrile (25 mL) at room temperature. Then triethylamine (1.73 mL, 12.49 mmol, 1.0 eq) was added drop wise at room temperature and the reaction mixture was heated to 80 °C and stirred for 16 h. The

reaction mixture was cooled to 0 °C and the resulting solids were filtered, dried under vacuum. The crude was purified by silica-gel (230-400 mesh) column chromatography using 2% MeOH/$CHCl_3$ as eluting solvent to yield ethyl 6-hydroxypyrimidine-4-carboxylate (**7d-III**) as brown solid (71%yield). LCMS-ESI (*m/z*): 169.0 [M + H]+.

Step 2: Synthesis of ethyl 6-chloropyrimidine-4-carboxylate (7d-IV)

Under nitrogen, thionylchloride (0.55 g, 4.46 mmol, 1.5 eq) was added to a solution of ethyl 6-hydroxypyrimidine-4-carboxylate (**7d-III)** (0.5 g, 2.976 mmol, 1.0 eq) in dry DMF (5 mL) at 0 °C. The reaction mixture was allowed to stir at 0 °C to 10 °C for 1 h. To the reaction mixture 50 mL of aq.sodiumbicarbonate was added and extracted with EtOAc (3×50 mL). The combined organic layer was washed with ice-cold water (50 mL × 3) dried over sodium sulphate, concentrated to yield crude product. The crude was purified by silica-gel (230-400 mesh) column chromatography using 10% EtOAc/pet ether as eluting solvent to yield ethyl 6-chloropyrimidine-4-carboxylate (**7d-IV**) as brown solid (36% yield). ^{1}H NMR (400 MHz, DMSO-d6) δ 9.24 (s, 1H), 8.17 (s, 1H), 4.39 (q, J = 7.2 Hz, 2H), 1.34 (t, J = 7.2 Hz, 3H); LCMS-ESI (*m/z*): 187.0 $[M + H]^+$.

Step 3: Synthesis of 6-phenylpyrimidine-4-carboxylic acid (7d)

Phenylboronic acid (0.65 g, 5.376 mmol, 1.0 eq) and sat.aq.$NaHCO_3$ (10 mL) were added to a stirred solution of ethyl 6-chloropyrimidine-4-carboxylate (**7d-IV**) (1.0 g, 5.376 mmol, 1.0 eq) in 1,4-dioxane (20 mL) at room temperature. The reaction mixture was de-oxygenated with argon for 30 min. To the reaction mixture tetrakis(triphenylphosphane)palladium (0) (621 mg, 0.537 mmol, 0.1 eq) was added and de-oxygenated with argon for another 30 min. The reaction mixture was allowed to stir at 90-100 °C for 20 h under argon. The reaction mixture was filtered over celite, washed with 50% methanol/$CHCl_3$ (100 mL) and the filtrate was concentrated. The residue was taken in water (20 mL) and washed with ethyl acetate (10 mL × 2). The aq.layer pH was adjusted to 6-7 using 1N HCl and the resulting solid was

filtered. The solid was dried to afford title compound (**7d**) as brown solid (74% yield). LCMS-ESI (*m/z*): 201.0 [M + H]$^+$.

Synthesis of indolizine-3-carboxylic acid (7e)

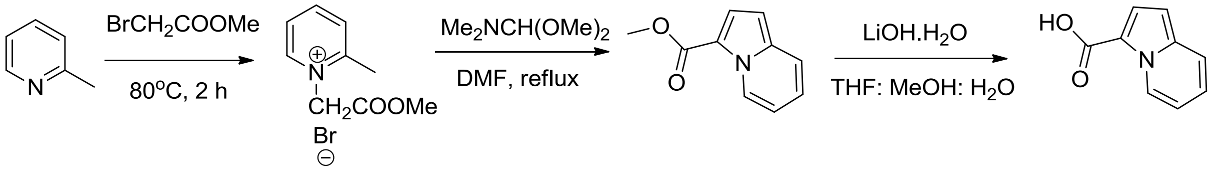

7e-I **7e-II** **7e-III** **7e**

Step 1 and Step 2: Synthesis of methyl indolizine-3-carboxylate (7e-III)

2-Methyl pyridine (9.4 g, 100.93 mmol, 1.0 eq.) and methyl bromo acetate (10.0 mL, 100.93 mmol, 1.0 eq.) were heated at 80 °C for 2 h. The reaction was cooled to room temperature. Then DMA (24 mL) and DMF (100 mL) were added and heated to reflux for 5 h. The reaction mixture was cooled to room temperature and diluted with EtOAc (400 mL) then washed with ice cold water (3 × 100 mL) and followed by brine solution. The organic layer was separated, dried over Na_2SO_4 and evaporated under vacuum. The resulting crude residue was purified by flash column chromatography on silica gel (230-400 mesh size) using petroleum ether/ethyl acetate (10-12%) as eluant. The desired product (**7e-III**) was isolated as pale brown liquid (29% yield for two steps); LCMS-ESI (*m/z*): 176.1 [M + H]$^+$.

Step 3: Synthesis of indolizine-3-carboxylic acid (7e)

LiOH.H_2O (1.2 g, 28.57 mmol, 2.0 eq.) was added to the stirred solution of methyl indolizine-3-carboxylate (**7e-III**) (2.5 g, 14.28 mmol, 1.0 eq.) in THF: MeOH: H_2O (7:2:1, 10 mL) at 0 °C. Then the reaction mixture was allowed to stir for 16 h. The solvents were evaporated under vacuum to get crude residue. Water (10 mL) was added and the aqueous layer was washed with ethyl acetate (3 × 30 mL). The aqueous layer was acidified pH 6 with 20% citric acid solution. The resulting solid was filtered and dried under vacuum. The desired product (**7e**) was isolated as white solid (67% yield); ^{1}H NMR (400 MHz, DMSO-d6) δ 9.39

(d, J = 7.2 Hz, 1H), 7.63 (d, J = 8.4 Hz, IH), 7.41(d, J = 4.0 Hz, 1H), 7.06 (t, J = 7.2 Hz, IH), 6.90 (t, J = 6.8 Hz, IH), 6.54 (d, J = 4.0 Hz, 1H), LCMS-ESI (m/z): 162.0 $[M + H]^+$.

Synthesis of indolizine-2-carboxylic acid (7f)

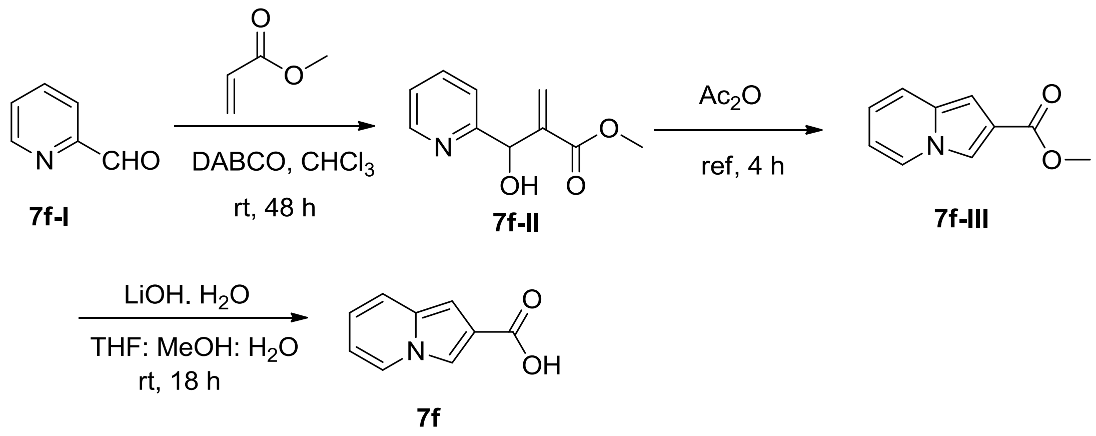

Step 1: Synthesis of methyl 2-(hydroxy(pyridin-2-yl)methyl)acrylate (7f-II)

Methyl acrylate (0.85 mL, 9.34 mmol, 1.0 eq.), and 1, 4-diazobicyclo (2,2,2) octane (52 mg, 0.467 mmol, 0.05 eq) were added to a suspension of picolinaldehyde (**7f-I**) (1 g, 9.34 mmol, 1 eq) in chloroform (10 mL) at room temperature. Then the reaction mixture was allowed stir at room temperature for 48 h. The reaction mixture was evaporated under vacuum to get crude residue. The resulting crude residue was purified by flash column chromatography on silica gel (230-400 mesh size) using petroleum ether/ethyl acetate (25%) as eluant. The desired product (**7f-II**) was isolated as dark yellow oil (44% yield); LCMS-ESI (m/z): 194.1 $[M + H]^+$.

Step 2: Synthesis of methyl indolizine-2-carboxylate (7f-III)

The suspension of methyl 2-(hydroxy(pyridin-2-yl)methyl)acrylate (**7f-II**) (500 mg, 2.56 mmol, 1.0 eq.) in acetic anhydride (6 mL) was refluxed for 4 h under nitrogen atmosphere. The reaction mixture was cooled to room temperature and poured in to the cold sat. aq. $NaHCO_3$ solution (20 mL) and stirred for 1 h. The aqueous layer was extracted with $CHCl_3$ (10 mL × 3) and the combined organic layer was dried over Na_2SO_4 and evaporated under

vacuo. The resulting crude residue was purified by flash column chromatography on silica gel (230-400 mesh size) using petroleum ether/ethyl acetate (5%) as eluant. The desired product (**7f-III**) was isolated as white solid (66% yield); LCMS-ESI (*m/z*): 176.0 $[M + H]^+$.

Step 3: Synthesis of indolizine-2-carboxylic acid (7f)

LiOH.H_2O (143 mg, 3.43 mmol, 2.0 eq.) was added to a stirred solution of methyl indolizine-2-carboxylate (**7f-III**) (300 mg, 1.714 mmol, 1.0 eq.) in THF: MeOH: H_2O (7:2:1, 10 mL) at 0 ^oC. Then the reaction mixture was allowed stir for 16 h. The solvents were evaporated under vacuum to get crude residue. Water (10 mL) was added and the aqueous layer was washed with ethyl acetate (3×30 mL). The aqueous layer was acidified to pH 6 with 20% citric acid solution. The resulting solid was filtered washed d with water and pet ether (20 mL) and dried under vacuum. Further the solid was purified by flash column chromatography on silica gel (230-400 mesh size) using petroleum ether/ethyl acetate (30%) as eluant. The desired product (**7f**) was isolated as white solid (180 mg, 62.0% yield); LCMS-ESI (*m/z*): 162.0 $[M + H]^+$.

Synthesis of lithium 5-phenyloxazole-2-carboxylate (7g)

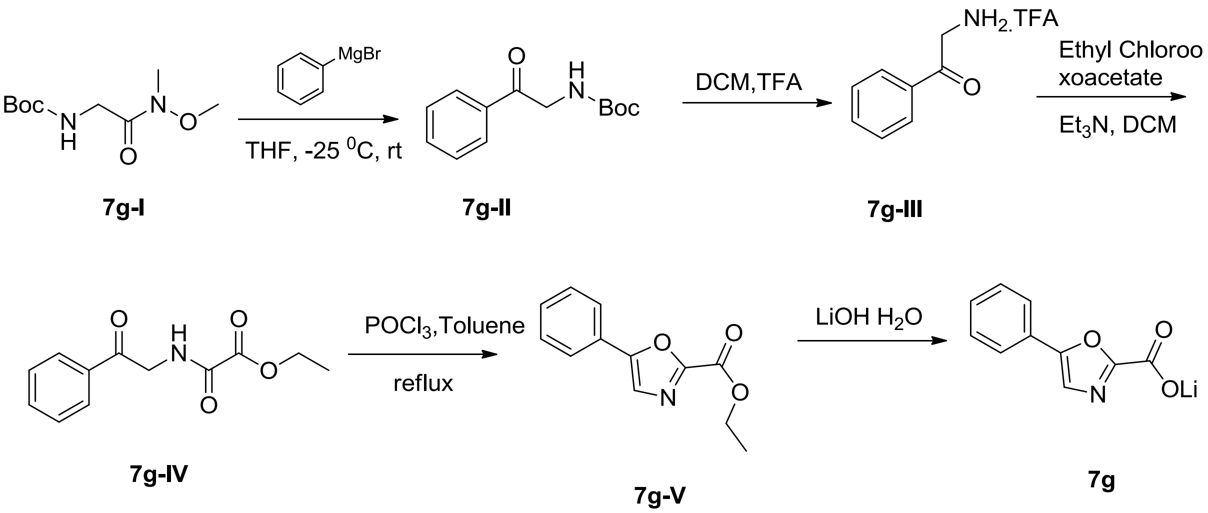

Step 1: Synthesis of tert-butyl (2-oxo-2-phenylethyl)carbamate (7g-II)

Under nitrogen, freshly prepared phenyl magnesium bromide (1 M in THF, 18.5 mL) was added drop-wise to a stirred solution of *tert*-butyl (2-(methoxy(methyl)amino)-2-oxoethyl)carbamate (**7g-I**) (2.0 g, 9.174 mmol, 1.0 eq.) in dry THF (25 mL), at -25 ^{o}C, The reaction mixture was stirred for 1 h at -25 ^{o}C then allowed to stir at room temperature for 16 h. The reaction mixture was cooled to 0 ^{o}C and sat. ammonium chloride solution was added drop-wise. The reaction mixture was extracted in to ethyl acetate (100 mL). The organic layer was washed with brine solution (20 mL), dried over sodium sulphate, concentrated to yield crude product. The crude was purified by silica-gel (230-400mesh) column chromatography using 15% EtOAc/Pet ether as eluting solvent to yield *tert*-butyl (2-oxo-2-phenylethyl)carbamate (**7g-II**) as a gummy liquid (74% yield); LCMS-ESI (*m/z*): 136.0 [M + H-100]$^{+}$ (de-boc mass was observed)

Step 2: Synthesis of 2,2,2-trifluoro-1-((2-oxo-2-phenylethyl)-l4-azanyl)ethan-1-one (7g-III)

Trifluoro acetic acid (0.72 g, 6.38 mmol, 3.0 eq) was added to a stirred solution of *tert*-butyl (2-oxo-2-phenylethyl)carbamate (**7g-II**) (0.5 g, 2.12 mmol, 1.0 eq.) in dichloromethane (10 mL) at 0 ^{o}C. The reaction mixture was allowed to stir at room temperature for 16 h. The reaction mixture was concentrated under vacuum and the residue was taken in diethyl ether (10 mL). The resulting solid was filtered, dried under vacuum to yield desired product as brown solid (**7g-III**) (76% yield); ^{1}H NMR (400 MHz, DMSO-d6) δ 8.33 (bs, 2H), 8.02 (t, *J*= 7.2 Hz, 2H), 7.76-7.58 (m, 3H), 4.62 (s, 2H).

Step 3: Synthesis of ethyl 2-oxo-2-((2-oxo-2-phenylethyl)amino)acetate (7g-IV)

Triethylamine (1.01 mL, 22 mmol, 3.0 eq) was added to a solution of 2,2,2-trifluoro-1-((2-oxo-2-phenylethyl)-l4-azanyl)ethan-1-one (**7g-III**) (0.6 g, 2.409 mmol, 1.0 eq.) in dry DCM (20 mL), at 0 ^{o}C and stirred for 5min. Then ethylchlorooxoacetate (0.361 g, 2.649 mmol, 1.1 eq) was added drop wise to the reaction mixture at 0 ^{o}C. The reaction mixture was allowed to

stir at 0 °C to 5 °C for 2 h. Ice cold water (50 mL) was added to the reaction mixture and extracted with DCM (50 mL). The organic layer was washed with brine solution (20 mL), dried over sodium sulphate, concentrated to yield crude product. The crude was purified by silica-gel (230-400 mesh) column chromatography using 40% EtOAc/Pet ether as eluting solvent to yield ethyl 2-oxo-2-((2-oxo-2-phenylethyl)amino)acetate (**7g-IV**) as pale brown solid (71% yield); ^{1}H NMR (400 MHz, $CDCl_3$) δ 8.07 (bs, 1H), 8.00 (d, *J*= 7.6 Hz, 2H), 7.67-7.51 (m, 3H), 4.83 (d, *J*= 4.4 Hz, 2H) 4.40 (q, *J*= 6.8 Hz, 2H), 1.42 (t, *J*= 6.8 Hz, 3H).

Step 4: Synthesis of ethyl 5-phenyloxazole-2-carboxylate (7g-V)

$POCl_3$ (0.23 mL, 2.55 mmol, 3.0 eq) was added to a stirred solution of ethyl 2-oxo-2-((2-oxo-2-phenylethyl)amino)acetate (**7g-IV**) (0.2 g, 0.851 mmol, 1.0 eq) in dry toluene (3 mL) at room temperature. The reaction mixture was refluxed for 16 h. The reaction mixture was cooled to 0 °C, and then carefully basified with Sat.aq.$NaHCO_3$ solution. The reaction mixture was extracted with ethyl acetate (20 mL). The organic layer was washed with brine solution (10 mL), dried over sodium sulphate, concentrated to yield crude product. The crude was purified by silica-gel (230-400 mesh) column chromatography using10% EtOAc/Pet ether as eluting solvent to yield ethyl 5-phenyloxazole-2-carboxylate (**7g-V**) as yellow solid (56% yield); ^{1}H NMR (400 MHz, $CDCl_3$) δ 7.70-7.68 (m, 2H), 7.45-7.19 (m, 4H), 4.43 (q, *J*= 6.8 Hz, 2H), 1.39 (t, *J*= 6.8 Hz, 3H).

Step 5: Synthesis of lithium 5-phenyloxazole-2-carboxylate (7g)

$LiOH.H_2O$ (0.17 g, 4.14 mmol, 3.0 eq) was added to a solution of ethyl 5-phenyloxazole-2-carboxylate (**7g-V**) (0.3 g, 1.382 mmol, 1.0 eq.) in MeOH: H_2O (10 mL: 2 mL) at 0 °C. The reaction mass was allowed to stir at rt for 2 h. The reaction mixture was concentrated under vacuum, co-distilled with toluene to yield lithium 5-phenyloxazole-2-carboxylate (**7g**) (crude 99% yield).

Synthesis of benzo[*d*]thiazole-7-carboxylic acid (7h)

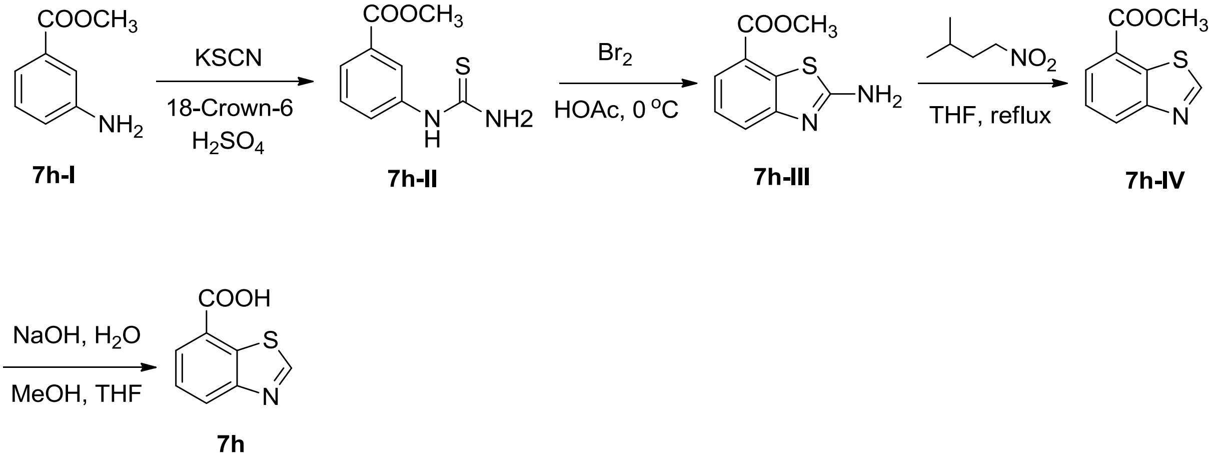

Step 1: Synthesis of methyl 3-thioureidobenzoate (7h-II)

Sulphuric acid (0.92 mL) was added drop wise to a stirred solution of methyl 3-aminobenzoate (**7h-I**) (5.0 g, 33.077 mmol, 1.0 eq), in chlorobezene (40 mL) at -10 °C. After 15 min potassium thiocyanate (KSCN) (3.37 g, 34.73 mmol, 1.05 eq) was added portion wise for 30 min and then 18-crown-6 (0.88 g, 3.307 mmol, 0.1 eq) was added at -10 °C. Then the reaction mixture was heated to 100 °C for 16 h. The reaction mixture was cooled to room temperature. The resulting solid was filtered and washed with chlorobezene (30 mL) and pet ether (50 mL). The residue was taken in water (30 mL) and stirred for 30 min. Then the solid was filtered and washed with water and dried under vacuum to yield the pale yellow color solid (**7h-II**) as desired product (89%); LCMS-ESI (*m/z*): $[M + H]^+$

Step 2: Synthesis of methyl 2-aminobenzo[*d*]thiazole-7-carboxylate (7h-III)

Bromine in acetic acid (7.0 mL, 7.14 mmol, 1.5 eq) was added drop wise to a stirred solution of methyl 3-thioureidobenzoate (**7h-II**) (1.0 g, 4.76 mmol, 1.0 eq) in AcOH (5 mL) at 0 °C and then the reaction mixture was heated to 75 °C for 4 h. The reaction mixture was cooled to room temperature and ether (15 mL) was added and the resulting solid was filtered. The solid was taken in sat. $NaHCO_3$ (10 mL) and stirred vigorously with for 15 min. The solid was filtered and washed with water and dried under vacuum to yield the methyl 2-

aminobenzo[d]thiazole-7-carboxylate (**7h-III**) as pale brown solid (42%); LCMS-ESI (*m/z*): $[M + H]^+$

Step 3: Synthesis of methyl benzo[*d*]thiazole-7-carboxylate (7h-IV)

Isopentyl nitrile (3.1 mL, 23.269 mmol, 2.2 eq) was added to a stirred solution of methyl 2-aminobenzo[d]thiazole-7-carboxylate (**7h-III**) (2.2 g, 10.576 mmol, 1.0 eq) in THF (30 mL) at room temperature and heated to reflux for 5 h. Solvent was evaporated under vacuum. The resulting crude product was purified by flash column chromatography on silica gel (230-400 mesh size) using petroleum ether/ethyl acetate (8-10%) as eluant to yield methyl benzo[*d*]thiazole-7-carboxylate (**7h-IV**) as pale yellow liquid (75% yield); LCMS-ESI (*m/z*): 194.1$[M + H]^+$

Step 4: Synthesis of benzo[*d*]thiazole-7-carboxylic acid (7h)

LiOH.H_2O (650 mg, 15.544 mmol, 2.0 eq) was added to the stirred solution of methyl benzo[d]thiazole-7-carboxylate (1.5 g, 7.772 mmol, 1.0 eq.) in THF: MeOH: H_2O (7:2:1, 30 mL) at 0 ^oC. Then the reaction mixture was allowed to stir for 16 h at room temperature. The reaction mixture was evaporated under vacuum to get crude residue. The crude residue was taken in 10 mL of water and acidified to pH 4 with 10% citric acid at 0 ^oC. The resulting solid was filtered and washed with H_2O (20 mL), pet ether (20 mL× 3), dried under vacuum to yield the benzo[d]thiazole-7-carboxylic acid (**7h**) as white solid (86% yield); LCMS-ESI (*m/z*): 180 $[M + H]^+$

Synthesis of piperidine-1-carbonyl chloride (7i)

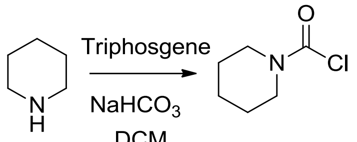

7i

Piperdine (3.52 mL, 35.22 mmol, 1.0 eq) in DCM (10 mL) was added drop wise to the stirred solution of triphosgene (7.31 g, 24.65 mmol, 0.7 eq) and $NaHCO_3$ (5.22 g, 70.45 mmol, 2.0

eq) in DCM (40 mL) at -10 °C and the reaction mixture was allowed to stir at rt for 6 h. The reaction mixture was filtered to remove the solids, and then the filtrate was concentrated under vacuum to get the desired product (**7i**) (4.5 g) which was used for next step without further purification.

Synthesis of 2-methyl-2-(1*H*-pyrrol-1-yl)propanoic acid (7k)

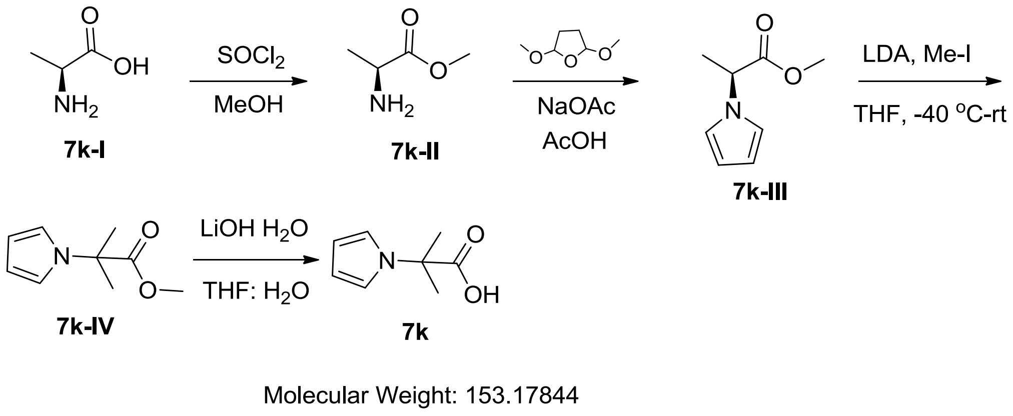

Step 1: Synthesis of methyl alaninate (7k-II)

$SOCl_2$ (12.43 mL, 168.36 mmol, 3.0 eq) was added to a stirred solution of alanine (**7k-I)** (5.0 g, 56.12 mmol 1.0 eq) in MeOH (50 mL) at RT and then the reaction mixture was heated to reflux for 16 h. The solvent was evaporated under vacuum to yield the methyl *L*-alaninate (**7k-II)** as yellow oil (quantitative yield). ^{1}H NMR (400 MHz, DMSO-d6) δ 8.52 (bs, 2H), 4.10 (bs, 1H), 3.74 (s, 3H), 1.41 (d, *J*= 7.2 Hz, 3H).

Step 2: Synthesis of methyl 2-(1H-pyrrol-1-yl)propanoate (7k-III)

NaOAc was added to a solution of 2,5-dimethoxytetrahydrofuran (2.56 mL, 19.38 mmol, 1.0 eq) and methyl alaninate (2.0 g, 19.38 mmole, 1.0 eq) in acetic acid (10 mL) at room temperature. Then the reaction mixture was heated at 80 °C for 3 h. Acetic acid was removed under vacuum and the residue was dissolved in ether (50 mL). The organic layer was washed with H_2O (2×50 mL) and dried over Na_2SO_4 and evaporated under vacuo. The resulting crude product was purified by flash column chromatography on silica gel (230-400 mesh size)

using DCM as eluent. The resulting product (**7k-III)** was isolated as dark yellow oil (27%). ^{1}H NMR (400 MHz, $CDCl_3$) δ 6.75 (t, *J*= 2.0 Hz, 2H), 6.19 (t, *J*= 2.0 Hz, 2H), 4.77 (q, *J*= 7.2 Hz, 1H), 3.72 (s, 3H), 1.73 (d, *J*= 7.2 Hz, 3H).

Step 3: Synthesis of methyl 2-methyl-2-(1H-pyrrol-1-yl)propanoate (7k-IV)

Under nitrogen, *n*-BuLi [2.1 mL, 2.155 mmol, 1.1 eq, 1.0 M in hexane] was added to a stirred solution of DIPA (0.357 mL, 2.547 mmol, 1.3 eq) in dry THF (5 mL) at 0 ^{o}C and stirred for 30 min at same temperature. The reaction mixture was cooled to -40 ^{o}C and then methyl 2-(1H-pyrrol-1-yl)propanoate (**7k-III**) (300 mg, 1.959 mmol, 1.0 eq) in dry THF (5 mL) was added and then stirred at 0 ^{o}C for 30 min. Again the reaction mixture was cooled to -40 ^{o}C and then iodomethane (0.183 mL, 2.94 mmol, 1.5 eq) was added to the reaction mixture and allowed to stir at 0 ^{o}C for 1 h. The reaction was quenched with sat.NH_4Cl (30 mL) at 0 ^{o}C and extracted with EtOAc (2×50 mL). The combined organic layer was washed brine (10 mL) and dried over Na_2SO_4.The solvent was evaporated under vacuum to yield the desired product (**7k-IV**) as yellow oil, which was used further without purification. ^{1}H NMR (400 MHz, $CDCl_3$) δ 6.80 (t, *J*= 2.4 Hz, 2H), 6.19 (t, *J*= 2.4 Hz, 2H), 3.69 (s, 3H), 1.79 (s, 6H).

Step 4: Synthesis of 2-methyl-2-(1H-pyrrol-1-yl)propanoic acid (7k-IV)

$LiOH.H_2O$ (2.017 g, 47.85 mmol, 2.0 eq.) was added to the stirred solution of methyl 2-methyl-2-(1*H*-pyrrol-1-yl)propanoate (**7k-III**) (4.0 g, 23.92 mmol, 1.0 eq.) in THF:H_2O (8:2, 40 mL) at 0 ^{o}C. Then the reaction mixture was allowed stirred at room temperature for 16 h. The reaction mixture was evaporated under vacuum to get crude residue. The crude reside was dissolved in H_2O (40 mL) and washed with ethylacetate (50 mL) to remove the impurities. The aqueous layer was acidified to pH 2 with 1N HCl and extracted with EtOAc (3 × 50 mL). The organic layer was evaporated under vacuum to yield the yellow oil (**7k-IV**) (49% yield). ^{1}H NMR (400 MHz, $CDCl_3$) δ 6.83 (t, *J*= 2 Hz, 2H), 6.20 (t, *J*= 2 Hz, 2H), 1.81 (s, 6H).

3. Synthesis of amine esters 8(b-d)

Synthesis of (*S*)-methyl 2-aminopropanoate hydrochloride (8b)

$SOCl_2$ (12.43 mL, 168.36 mmol, 3.0 eq) was added to a stirred solution of *L*-alanine (5.0 g, 56.12 mmol 1.0 eq) in MeOH (50 mL) at RT and then the reaction mixture was allowed to reflux for 16 h. The solvent was evaporated under vacuum to yield the methyl L-alaninate (**8b**) as yellow oil (quantitative yield). ^{1}H NMR (400 MHz, DMSO-d6) δ 8.52 (bs, 2H), 4.10 (bs, 1H), 3.74 (s, 3H), 1.41 (d, *J*= 7.2, Hz, 3H).

Synthesis of *L*-Tyrosine methyl ester hydrochloride (8c)

Under nitrogen, thionyl chloride (6.87 mL, 82.87 mmol, 3.0 eq) was added drop wise to a suspension of L-tyrosine (5 g, 27.62 mmol, 1.0 eq) in methanol (40 mL) at rt. This mixture was heated to reflux for 16 h. The solvent were evaporated in vacuum and co-distilled with pet ether (2 × 20 mL), to get the methyl L-tyrosinate (**8c**) as a half white solid (6.5 g, quantitative yield); ^{1}H NMR (400 MHz, DMSO-d6) δ 8.68 (bs, 3H), 6.99 (d, *J*=8.4 Hz, 2H), 6.73 (d, *J*=8.4 Hz, 2H), 4.12 (bs, 1H), 3.65 (s, 3H), 3.08 (dd, *J*= 6.0, 14.4 Hz, 1H), 2.99 (dd, *J*= 7.2, 14.0 Hz, 1H).

Synthesis of [(S)-methyl 2-amino-3-(pyridin-2-yl)propanoate hydrochloride] (8d)

Under nitrogen, thionyl chloride (4.67 mL, 56.39 mmol, 3 eq) was added drop wise to a suspension of (*S*)-2-((tert-butoxycarbonyl)amino)-3-(pyridin-2-yl)propanoic acid (5.0 g, 18.79 mmol, 1.0 eq) in methanol (50 mL) at room temperature. The solvent was evaporated in vacuum and washed with pet ether to yield the desired compound as off-white solid, which was used for next step without further purification (quantitative yield); ^{1}H NMR (400 MHz, DMSO-d6) δ 9.04 (bs, 2H), 8.76 (d, *J*= 4.8 Hz, 1H), 8.37 (t, *J*= 7.6 Hz, 1H), 7.95 (d, *J*= 8.0 Hz, 1H), 7.81 (t, *J*= 6.4 Hz, 1H), 4.66 (m, 1H), 3.66 (bs, 5H); LCMS-ESI (*m/z*): 181.1 $[M + H]^+$.

4. Synthesis of CAP esters 9(a-o)

Synthesis of (*S*)-methyl 3-phenyl-2-(2-phenylacetamido)propanoate (9a)

Under nitrogen, *N*,*N*-diisopropylethylamine (DIPEA) (13.5 mL, 77.14 mmol, 3.5 eq.) was added drop wise to the stirred suspension 2-phenylacetic acid (**7b**) (3.0 g, 22.04 mmol, 1.0 eq.), *L*-Phenylalanine methyl ester hydrochloride (**8a**) (4.74 g, 22.04 mmol, 1.0 eq) and 2-(1*H*-Benzotriazole-1-yl)-1,1,3,3-tetramethyluronium tetrafluoroborate (9.2 g, 28.65 mmol, 1.3 eq.) in dry CH_2Cl_2 (70 mL) at 0 °C. The reaction mixture slowly warm to room temperature and was allowed to stir for 6 h at room temperature. The reaction mixture was diluted with CH_2Cl_2 (50 mL), washed with aq.$NaHCO_3$ (100 mL), H_2O (2 × 100 mL) and brine solution (100 mL). The organic layer was separated, dried over Na_2SO_4 and evaporated under vacuum. The resulting crude product was purified by flash column chromatography on silica gel (230-400 mesh size) using petroleum ether/ethyl acetate (25-30%) as eluent to give (**9a**) as a off white solid (78% yield). ^{1}H NMR (400 MHz, $CDCl_3$) δ 7.49-7.30 (m, 2H), 7.28-7.20 (m, 4H), 7.12 (dd, *J*= 1.6, 6.4 Hz, 2H), 6.63 (bs, 1H), 6.97-6.93 (m, 2H), 4.78-4.75 (m, 1H), 3.75 (s, 3H), 3.61 (s, 2H), 3.19-3.01 (m, 2H).

Following a procedure similar to that of **9a**, the following ester intermediates were synthesized.

(*S*)-methyl 2-benzamido-3-phenylpropanoate (9b)

^{1}H NMR (400 MHz, $CDCl_3$) δ 7.71 (dd, *J*= 1.6, 6.8 Hz, 2H), 7.50-7.38 (m, 3H), 7.30-7.21 (m, 3 H), 7.12 (dd, *J*= 1.6, 6.4 Hz, 2H), 6.63 (bs, 1H), 5.10-5.07 (m, 1H), 3.69 (s, 3H), 3.22-3.03 (m, 2H).

(*S*)-methyl 3-phenyl-2-(2-phenylpyrimidine-5-carboxamido)propanoate (9c)

Yield: 55%. ^{1}H NMR (400 MHz, $CDCl_3$) δ 9.01 (s, 1H), 8.48 (dd, *J* = 8.0, 2.0 Hz, 2H), 7.55-7.48 (m, 3H), 7.34-7.27 (m, 3H), 7.13 (t, *J* =2.0 Hz, 2H), 6.62 (d, *J* = 7.2 Hz, 1H), 5.11 (q, *J* = 5.6 Hz, 1H), 3.81 (s, 3H), 3.35-3.22 (m, 2H); LCMS-ESI (*m/z*): 362.2 [M + H]+.

(*S*)-methyl 3-phenyl-2-(6-phenylpyrimidine-4-carboxamido)propanoate (9d)

Yield: 52%. LCMS-ESI (*m/z*): 362.2 $[M + H]^+$

(*S*)-methyl 2-(indolizine-3-carboxamido)-3-phenylpropanoate (9e)

Yield: 67%. LCMS-ESI (*m/z*): 323.2 $[M + H]^+$.

(*S*)-methyl 2-(indolizine-2-carboxamido)-3-phenylpropanoate (9f)

Yield: 67%; LCMS-ESI (*m/z*): 323.2 $[M + H]^+$.

(*S*)-methyl 2-(benzo[*d*]thiazole-7-carboxamido)-3-phenylpropanoate (9h)

Yield: 68%. LCMS-ESI (*m/z*): 341.1 $[M + H]^+$

(*S*)-methyl 2-(2-fluorobenzamido)-3-phenylpropanoate (9j)

Yield: 84%. ^{1}H NMR (400 MHz, $CDCl_3$) δ 8.05 (td, *J*= 1.6, 7.6, Hz, 1H), 7.49-7.44 (m, 1H), 7.32-7.16 (m, 6H), 7.10 (dd, *J*= 12.0, 8.4 Hz, 1H), 5.11-5.06 (m, 1H), 3.75 (s, 3H), 3.30-3.18 (m, 2H).

(*S*)-methyl 2-(2-methyl-2-(1*H*-pyrrol-1-yl)propanamido)-3-phenylpropanoate (9k)

Yield: 62%. ^{1}H NMR (400 MHz, $CDCl_3$) δ 7.27-7.22 (m, 3H), 6.94-6.92 (m, 2H), 6.73 (t, *J*= 2.0 Hz, 2H), 6.23 (t, *J*= 2.0 Hz, 2H), 5.41 (d, *J*= 7.6 Hz, 1H) 4.72-4.67 (m, 1H), 3.68 (s, 3H), 3.04 (dd, *J*= 14.0, 5.2 Hz, 1H), 2.86 (dd, *J*= 14.0, 7.2 Hz, 1H), 1.74 (s, 3H), 1.66 (s, 3H).

(*S*)-methyl 3-phenyl-2-(4-(trifluoromethyl)benzamido)propanoate (9l)

Yield: 81%. ^{1}H NMR (400 MHz, $CDCl_3$) δ 7.81 (d, *J*= 8.4 Hz, 2H), 7.68 (d, *J*= 8.4 Hz, 2H), 7.32-7.24 (m, 3H), 7.12 (dd, *J*= 1.6, 7.6 Hz, 2H), 6.62 (d, *J*= 7.2 Hz, 1H), 5.11-5.06 (m, 1H), 3.79 (s, 3H), 3.22-3.20 (m, 2H).

(*S*)-methyl 2-(pyrazine-2-carboxamido)propanoate (9m)

Yield: 84%. LCMS-ESI (*m/z*): 210.1 $[M + H]^+$.

(*S*)-methyl 3-(4-hydroxyphenyl)-2-(pyrazine-2-carboxamido)propanoate (9n)

Yield: 67%. ^{1}H NMR (400 MHz, $CDCl_3$) δ 9.34 (d, *J*=1.2 Hz, 1H), 8.74 (d, *J*=1.2 Hz, 1H), 8.52 (m, 1H), 8.22 (d, *J*=8.0 Hz, 1H), 6.98 (d, *J*=6.4 Hz, 2H), 6.73 (d, *J*=8.4 Hz, 2H), 5.02 (m, 1H), 3.74 (s, 3H), 3.15 (m, 2H).

(*S*)-methyl 2-(pyrazine-2-carboxamido)-3-(pyridin-2-yl)propanoate (9o)

Yield: 63%. ^{1}H NMR (400 MHz, $CDCl_3$) δ 9.35 (d, J=1.6 Hz, 1H), 9.05 (d, J=7.6 Hz, 1H), 8.72 (d, J = 2.4 Hz, 1H), 8.55 (m, 2H), 7.60 (dt, J=1.6, 7.6 Hz, 1H), 7.17 (m, 2H), 5.19 (m, 1H), 3.71 (s, 3H), 3.49 (dd, J=8.0, 14.8 Hz, 1H), 3.40 (dd, J=5.2, 15.2 Hz, 1H).

(*S*)-methyl 3-phenyl-2-(piperidine-1-carboxamido)propanoate (9i)

Diisopropyl ethyl amine (2.36 mL, 13.94 mmol, 3.0 eq.) was added to a solution of L-phenylalanine methyl ester hydrochloride (8**a**) (1.0 g, 4.647 mmol, and 1.0 eq) in CH_2Cl_2 (15 mL) at 0 °C. Then the reaction mixture was stirred at the same temperature for 30 min, and then piperidine-1-carbonyl chloride (**7i**) (683 mg, 4.647 mmol, 1.0 eq) in CH_2Cl_2 (5 mL) was added. Then the reaction mixture was allowed to stir at room temperature for 16 h. The reaction mixture was diluted with CH_2Cl_2 (10 mL), washed with H_2O (2 × 10 mL), brine solution (10 mL), then the organic layer was separated, dried over Na_2SO_4 and evaporated under vacuo. The resulting crude product was purified by flash column chromatography on silica gel (230-400 mesh size) using petroleum ether/ethyl acetate (25-30%) as eluent. The resulting product (**9i**) was isolated as off white solid (58% yield). ^{1}H NMR (400 MHz, $CDCl_3$) δ 7.31-7.21 (m, 3H), 7.12-7.10 (m, 2H), 4.85-4.76 (m, 2H), 3.71 (s, 3H), 3.33-3.22 (m, 4H), 3.17-3.10 (m, 2H), 1.61-1.48 (m, 6H). LCMS-ESI (*m/z*): 291.2 $[M + H]^+$.

(*S*)-methyl 3-phenyl-2-(5-phenyloxazole-2-carboxamido)propanoate (9g)

Under nitrogen, lithium 5-phenyloxazole-2-carboxylate (**7g**) (450 mg, 2.307 mmol, 1.0 eq.) was taken in dry CH_2Cl_2 (10 mL) and dry DMF (5 mL). The reaction mixture was cooled to 0 °C, and then *N,N*-diisopropylethylamine (0.89 g, 6.92 mmol, 3.0 eq) was added followed by TBTU (0.74 g, 2.307 mmol, 1.0 eq) and L-phenylalanine methyl ester hydrochloride (**8a**) (0.49 g, 2.307 mmol, 1.0 eq) under nitrogen. After stirring at room temperature for 48 h, 25 mL of water was added to the reaction mixture and extracted with CH_2Cl_2 (2×25 mL). The combined organic layer was washed with brine solution (20 mL), dried over sodium sulphate. The solvent was removed under reduced pressure and the resulting crude was purified by

silica-gel (230-400 mesh) column chromatography using 20% EtOAc/Pet ether as eluting solvent to yield (*S*)-methyl (5-phenyloxazole-2-carbonyl)-L-phenylalaninate (**9g**) as a gummy liquid (41% yield). ^{1}H NMR (400 MHz, $CDCl_3$) *δ* 7.78 (d, *J* = 7.2 Hz, 2H), 7.51-7.19 (m, 7H), 7.11 (d, *J* = 7.2 Hz, 2H), 5.10-4.90 (m, 1H), 3.78 (s, 3H), 3.31-3.15 (m, 2H).

5. Synthesis of CAP carboxylic acids 10(a-o)

Synthesis of (*S*)-3-phenyl-2-(2-phenylacetamido)propanoic acid (10a)

LiOH.H_2O (1.49 g, 35.33 mmol, 3.0 eq.) was added to the stirred solution of (*S*)-methyl (2-phenylacetyl)-L-phenylalaninate (**9a**) (3.5 g, 11.77 mmol, 1.0 eq.) in THF: H_2O (8:2, 20 mL) at 0 ^oC, then the reaction mixture was allowed stirred at room temperature for 16 h. The solvents were evaporated under vacuum. To the residue, water was added and acidified with 20% citric acid solution at 0 ^oC. The resulting solids were filtered, washed with water (20 mL). The solids were co-distilled with toluene (20 mL × 2) to afford **10a** as white solid (45% yield). ^{1}H NMR (400 MHz, DMSO-d6) *δ* 8.39 (d, *J*= 8.0 Hz, 1H), 7.26-7.18 (m, 8 H), 7.11 (d, *J*= 6.8 Hz, 1H), 4.45-4.42 (m, 1H), 3.40 (d, *J*= 13.6 Hz, 2H), 3.06 (dd, *J*= 14.0, 4.8 Hz, 1H), 2.86 (dd, *J*= 13.6, 9.6 Hz, 1H); ^{13}C NMR (400 MHz, DMSO-d6) *δ* 172.9, 169.9, 137.5, 136.1, 129.1, 128.9, 128.1, 128.0, 126.3, 126.1, 53.5, 41.9, 36.8; LCMS-ESI (*m/z*): 284.4$[M + H]^+$.

Synthesis of (*S*)-2-benzamido-3-phenylpropanoic acid (10b)

LiOH.H_2O (720 mg, 16.95 mmol, 2.0 eq.) was added to the stirred solution (*S*)-methyl 2-benzamido-3-phenylpropanoate (**9b**) (2.2 g, 8.475 mmol, 1.0 eq.) in THF: H_2O (8:2, 20 mL) at 0 ^oC. The reaction mixture was allowed to stir at room temperature for 2 h. The solvents were evaporated under vacuum. To the residue, water was added and acidified with 20% citric acid solution at 0 ^oC. The resulting solids were filtered, washed with water (20 mL). The solids were co-distilled with toluene (20 mL × 2) to afford (**10b**) as a half white solid

(67% yield). ^{1}H NMR (400 MHz, DMSO-d6) δ 8.65 (d, *J*= 8.0 Hz, 1H), 7.79 (d, *J*= 7.6 Hz, 2H), 7.53-7.15 (m, 8H), 4.64-4.59 (m, 1H), 3.22-3.04 (m, 2H); ^{13}C NMR (400 MHz, DMSO-d6) δ 173.4, 166.5, 138.4, 134.2, 131.4, 129.2, 128.39, 128.32, 127.4, 126.4, 54.4, 36.5; LCMS-ESI (*m/z*): 270.1[M + H]$^+$.

Synthesis of (*S*)-3-phenyl-2-(2-phenylpyrimidine-5-carboxamido)propanoic acid (10c)

To a solution of (S)-methyl 3-phenyl-2-(2-phenylpyrimidine-5-carboxamido)propanoate (**9c**) (1.1 g, 3.04 mmol, 1 eq) in methanol (20 mL), at rt, solution of LiOH.H_2O (0.384 g, 9.14 mmol, 3 eq) in 10 mL of water was added. The reaction mass was allowed to stir at rt for 3 h. The reaction mass was concentrated under vacuum, To the residue, water was added and acidified with 20% citric acid solution at 0 °C. The solids were filtered, washed with water (20 mL), pentane (20 mL) and dried under vacuum to yield desired product (**10c**) (81% yield). ^{1}H NMR (400 MHz, DMSO-d6) δ 12.96 (bs, 1H), 9.18 (d, *J* = 5.6 Hz, 3H), 8.44 (d, *J* = 7.2, 1.2 Hz, 2H), 7.61-7.54 (m, 3H), 7.35-7.18 (m, 3H), 4.70-4.64 (m, 1H), 3.24 (dd, *J* = 14.0, 4.8 Hz, 1H), 3.05 (dd, *J* = 13.6, 10.0 Hz, 1H); LCMS-ESI (*m/z*): 348.2 [M + H]+.

Synthesis of (*S*)-3-phenyl-2-(6-phenylpyrimidine-4-carboxamido)propanoic acid (10d)

LiOH.H_2O (0.384 g, 9.14 mmol, 3.0 eq) was added to the solution of (*S*)-methyl 3-phenyl-2-(6-phenylpyrimidine-4-carboxamido)propanoate (**9d**) (1.1 g, 3.047 mmol, 1.0 eq) in methanol (30 mL), THF (10 mL) and water (5 mL), at 0 °C. Then the reaction mass was allowed to stir at rt for 20 h. The reaction mixture was concentrated under vacuum. To the residue, 10 mL of water was added and washed with EtOAc (20 mL × 2). The aqueous layer was separated and pH was adjusted to 2-3 using 20% citric acid solution. The solids were filtered, washed with water (20 mL). The solids were co-distilled with toluene (20 mL × 2) to afford 6-phenylpyrimidine acid (**10d**) as white solid (61%). ^{1}H NMR (400 MHz, DMSO-d6) δ 13.10 (bs, 1H), 9.37 (d, *J* = 1.6 Hz, 1H), 9.03 (d, *J* = 8.0 Hz, 1H), 8.44 (s, 1H), 8.28-8.26 (m, 1H),

7.62-7.55 (m, 3H), 7.26-7.16 (m, 5H), 4.79-4.74 (m, 1H), 3.25-3.23 (m, 2H); LCMS-ESI (*m/z*): 348.2 [M + H]+.

Synthesis of (*S*)-2-(indolizine-3-carboxamido)-3-phenylpropanoic acid (10e)

LiOH.H_2O (140 mg, 3.416 mmol, 2.0 eq.) was added to the stirred solution of (*S*)-methyl 2-(indolizine-3-carboxamido)-3-phenylpropanoate (**9e**) (550 mg, 1.708 mmol, 1.0 eq.) in THF: MeOH: H_2O (7:2:1, 20 mL) at 0 °C. Then the reaction mixture was allowed stir for 16 h. The solvents were evaporated under vacuum to get crude residue. To the residue, water was added and pH was adjusted to 2-3 using 20% citric acid solution at 0 °C. The solids were filtered, washed with water (20 mL) and dried. The residue was purified by flash column chromatography on silica gel (230-400 mesh size) using petroleum ether/ethyl acetate (30-35%) as eluant. The desired product was isolated as grey color solid (**10e**) (77% yield); ^{1}H NMR (400 MHz, DMSO-d6) δ 12.74 (bs 1H), 9.45 (d, *J* = 7.6 Hz, 1H), 8.37 (d, *J* = 8.8 Hz, 1H), 7.64 (d, *J* = 4.0 Hz, IH), 7.57 (d, *J* = 9.2 Hz, 1H), 7.34-7.14 (5H, m), 6.99-6.95 (m, 1H), 6.80-6.76 (m, 1H), 6.51 (d, *J* = 4.8 Hz, 1H), 4.65-4.60 (1H, m), 3.18 (dd, *J* = 14.0, 4.8 Hz, 1H) 3.06 (dd, *J* = 13.2, 10.4 Hz, 1H); LCMS-ESI (*m/z*): 309.1 $[M + H]^+$.

Synthesis of (*S*)-2-(indolizine-2-carboxamido)-3-phenylpropanoic acid (10f)

LiOH.H_2O (156.3 mg, 3.73 mmol, 2.0 eq) was added to the stirred solution of (S)-methyl 2-(indolizine-2-carboxamido)-3-phenylpropanoate (**10f**) (600 mg, 1.86 mmol, 1.0 eq) in THF: MeOH: H_2O (7:2:1, 20 mL) at 0 °C. Then the reaction mixture was allowed to stir for 4 h. The solvents were evaporated under vacuum to get crude residue. To the residue, water was added and pH was adjusted to 2-3 using 20% citric acid solution at 0 °C. The solids were filtered, washed with water (20 mL) and pet ether (10 mL× 3), dried under vacuum to get the desired product as off white solid (81% yield); ^{1}H NMR (400 MHz, DMSO-d6) δ 8.35 (d, *J* = 8.8 Hz, 1H), 8.25 (d, *J* = 7.6 Hz, IH), 7.93 (bs, 1H), 7.42 (d, *J* = 9.2 Hz, 1H), 7.31-7.14 (5H, m), 6.77 (s, 1H), 6.71 (dd, *J* =8.8, 6.8 Hz, 1H), 6.58 (t, *J* = 6.0 Hz, 1H), 4.60-4.55 (1H, m),

3.19-3.15 (dd, *J* = 13.2, 4.0 Hz, 1H) 3.08-3.02 (dd, *J* = 13.2, 10.0 Hz, 1H); LCMS-ESI (*m/z*): 309.1 $[M + H]^+$.

Synthesis of (*S*)-3-phenyl-2-(5-phenyloxazole-2-carboxamido)propanoic acid (10g)

To a solution of methyl (5-phenyloxazole-2-carbonyl)-L-phenylalaninate (**9g**) (0.5 g, 1.42 mmol, 1.0 eq) in MeOH (10 mL), 0 °C, LiOH.H_2O (0.12 g, 2.87 mmol, 2.0 eq), water (2 mL) were added. The reaction mass was allowed to stir at 0 °C for 2 h. The reaction mass was concentrated under vacuum, and to the residue, water was added and pH was adjusted to 2-3 using 20% citric acid solution at 0 °C. The solids were filtered, washed with water (20 mL) and dried. The solid was filtered, co-distilled with toluene, diethyl ether to yield (5-phenyloxazole-2-carbonyl)-L-phenylalanine (**10g**) as pale brown solid (83% yield). ^{1}H NMR (400 MHz, DMSO-d6) δ 8.90 (d, *J* = 7.6 Hz, 1H), 7.09 (s, 1H), 7.82 (d, *J* = 7.2 Hz, 2H), 7.54-7.17 (m, 8H), 4.62-4.59 (m, 1H), 3.38-3.14 (m, 2H); ^{13}C NMR (400 MHz, DMSO-d6) δ 172.3, 154.3, 153.6, 152.6, 139.6, 129.5, 129.1, 129.0, 128.1, 126.3, 124.5, 123.4, 54.1, 36.1; LCMS-ESI (*m/z*): 337.0 $[M + H]^+$

Synthesis of (*S*)-2-(benzo[*d*]thiazole-7-carboxamido)-3-phenylpropanoic acid (10h)

LiOH.H_2O (74 mg, 1.764 mmol, 2.0 eq.) was added to the stirred solution of methyl (benzo[d]thiazole-7-carbonyl)-L-phenylalaninate (**9h**) (300 mg, 0.882 mmol, 1.0 eq.) in THF: MeOH: H_2O (7:2:1, 10 mL) at 0 °C. Then the reaction mixture was allowed stir for 5 h at room temperature. The reaction mass was concentrated under vacuum and to the residue, water was added and pH was adjusted to 2-3 using 20% citric acid solution at 0 °C. The solid was filtered and washed with H_2O (5 mL), pet ether (10 mL× 3), dried under vacuum to get the (benzo[*d*]thiazole-7-carbonyl)-L-phenylalanine product (**10h**) as off white solid (63% yield); ^{1}H NMR (400 MHz, DMSO-d6) δ 12.73 (bs, 1H), 9.43 (s, IH), 9.15 (d, *J* = 8.0 Hz, 1H), 8.26 (d, *J* = 8.4 Hz, 1H), 8.18 (d, *J* = 7.2 Hz, IH), 7.69 (d, *J* = 7.2 Hz, IH), 7.35-7.14

(5H, m), 4.73-4.67 (1H, m), 3.24 (dd, *J* = 14.0, 4.4 Hz, 1H) 3.13 (dd, *J* = 14, 11.2 Hz, 1H); LCMS-ESI (*m/z*): 327.1 $[M + H]^+$

Synthesis of (S)-3-phenyl-2-(piperidine-1-carboxamido)propanoic acid (10i)

LiOH.H_2O (828 mg, 19.64 mmol, 3.0 eq.) was added to the stirred solution of methyl (piperidine-1-carbonyl)-L-phenylalaninate (**9i**) (1.9 g, 6.548 mmol, 1.0 eq.) in THF: H_2O (8:2, 20 mL) at 0 ^oC, then the reaction mixture was allowed stirred at room temperature for 6 h. The reaction mass was concentrated under vacuum and to the residue, water was added and pH was adjusted to 2-3 using 20% citric acid solution at 0 ^oC. The solid was filtered and washed with H_2O (5 mL), pet ether (10 mL× 3), dried under vacuum. The desired product (**10i**) was isolated as off white solid (72% yield). ^1H NMR (400 MHz, DMSO-d6) δ 7.24-7.17 (m, 5H), 6.44 (d, *J*= 7.6 Hz, 1H), 4.22-4.17 (m, 1H), 3.21-2.90 (m, 6H), 1.50-1.34 (m, 6H); ^{13}C NMR (400 MHz, DMSO-d6) δ 174.4, 156.9, 138.7, 129.2, 127.8, 125.9, 55.5, 44.3, 36.8, 25.2, 24.0; LCMS-ESI (*m/z*): 277.2 $[M + H]^+$.

Synthesis of (S)-2-(2-fluorobenzamido)-3-phenylpropanoic acid (10j)

LiOH.H_2O (630 mg, 14.95 mmol, 2.5 eq.) was added to the stirred solution of methyl (2-fluorobenzoyl) phenylalaninate (**9j**) (1.8 g, 5.98 mmol, 1.0 eq.) in THF: H_2O (8:2, 20 mL) at 0 ^oC for 0.5 h, Then the reaction mixture was allowed stirred at room temperature for 1 h. The solvents were evaporated under vacuum to get crude residue. The reaction mixture was concentrated under vacuum and to the residue water was added and pH was adjusted to 2-3 using 20% citric acid solution at 0 ^oC. The solid was filtered and washed with H_2O (5 mL), pet ether (10 mL× 3), dried under vacuum. The desired product (**10j**) was isolated as white solid (73% yield). ^1H NMR (400 MHz, DMSO-d6) δ 8.33-8.03 (m, 1H), 7.57-7.48 (m, 2H), 7.29-7.16 (m, 7H), 4.57-4.52 (m, 1H), 3.19 (dd, *J*= 13.6, 4.8 Hz, 1H), 3.03 (dd, *J*= 14.0, 4.8 Hz, 1H); ^{13}C NMR (400 MHz, DMSO-d6) δ 172.6, 163.0, 137.9, 132.6, 130.1, 129.2, 127.9, 126.2, 124.4, 123.2, 116.2, 115.9, 54.5, 36.5; LCMS-ESI (*m/z*): 288.1$[M + H]^+$.

Synthesis of (S)-2-(2-methyl-2-(1*H*-pyrrol-1-yl)propanamido)-3-phenylpropanoic acid (10k) $LiOH.H_2O$ (429 mg, 10.18 mmol, 2.0 eq.) was added to the stirred solution of methyl (2-methyl-2-(1H-pyrrol-1-yl)propanoyl)-L-phenylalaninate (**9k**) (1.6 g, 5.09 mmol, 1.0 eq.) in THF: H_2O (7:3, 20 mL) at 0 ^oC, then the reaction mixture was allowed stirred at room temperature for 2 h. The reaction mixture was concentrated under vacuum and to the residue water was added and pH was adjusted to 2-3 using 20% citric acid solution at 0 ^oC. The solid was filtered and washed with H_2O (5 mL), pet ether (10 mL× 3), dried under vacuum. The desired product (**10k**) was isolated as off white solid (80% yield). ^1H NMR (400 MHz, DMSO-d6) δ 7.25-7.06 (m, 3H), 7.07 (d, *J*= 6.8 Hz, 1H), 6.85 (d, *J*= 8.0 Hz, 1H), 6.72 (t, *J*= 2.0 Hz, 1H), 6.01 (t, *J*= 2.0 Hz, 1H), 4.44-4.39 (m, 1H), 3.02 (dd, *J*= 13.6, 4.8 Hz, 1H), 2.89 (dd, *J*= 13.6, 8.8 Hz, 1H), 1.53 (d, *J*= 2.8 Hz, 6H).^{13}C NMR (400 MHz, DMSO-d6) δ 172.7, 172.3, 137.1, 129.1, 128.0, 126.3, 118.3, 108.1, 61.2, 53.2, 36.1, 26.3, 26.1; LCMS-ESI (*m/z*): 301.1 $[M + H]^+$.

Synthesis of (*S*)-3-phenyl-2-(4-(trifluoromethyl)benzamido)propanoic acid (10l)

$LiOH.H_2O$ (394 mg, 9.401 mmol, 3.0 eq.) was added to the stirred solution of methyl (4-(trifluoromethyl)benzoyl)-L-phenylalaninate (**9l**) (1.4 g, 3.133 mmol, 1.0 eq.) in THF:H_2O (8:2, 20 mL) at 0 ^oC for 1 h. The reaction mixture was concentrated under vacuum. To the residue water was added and pH was adjusted to 2-3 using 20% citric acid solution at 0 ^oC. The solid was filtered and washed with H_2O (5 mL), pet ether (10 mL× 3), dried under vacuum. The desired product (**10l**) was isolated as half white solid (82% yield). ^1H NMR (400 MHz, DMSO-d6) δ 8.41 (d, *J*= 7.6 Hz, 1H), 7.94 (d, *J*= 8.0 Hz, 2 H), 7.77 (d, *J*= 8.4 Hz, 2H), 7.24-7.08 (m, 5H), 4.43-4.40 (m, 1H), 3.24 (dd, *J*= 13.2, 4.0 Hz, 1H), 3.05 (dd, *J*= 13.2, 8.4 Hz, 1H); ^{13}C NMR (400 MHz, DMSO-d6) δ 173.6, 164.3, 139.3, 138.6, 130.9, 130.6, 129.2, 127.9, 127.7, 125.7, 125.2, 125.1, 122.6, 56.0, 37.2; LCMS-ESI (*m/z*): 336.1$[M - H]^+$.

Synthesis of (*S*)-2-(pyrazine-2-carboxamido)propanoic acid (10m)

LiOH.H_2O (1.2 g, 28.68 mmol, 3.0 eq.) was added to the stirred solution of methyl (pyrazine-2-carbonyl)-L-alaninate (**9m**) (2.0 g, 9.56 mmol, 1.0 eq.) in THF:: H_2O (8:2, 30 mL) at 0 ^oC, then the reaction mixture was allowed stirred at room temperature for 1 h. Then the reside was dissolved in H_2O (20 mL), washed with ethyl acetate. The aqueous layer was acidified to pH 2 with 1N HCl and extracted with 10% MeOH/$CHCl_3$ (3×100 mL) and the organic solvents were removed under vacuum to yield a gummy liquid which was washed with ether (50 mL) to yield **10m** as a white solid (30% yield). ^1H NMR (400 MHz, DMSO-d6) δ 12.80 (bs, 1H), 9.18 (s, 1H), 8.96 (d, *J*= 7.2 Hz, 1H), 8.90 (d, *J*= 2.4 Hz, 1H), 8.76-8.75 (m, 1H), 4.49 (t, *J*= 7.2 Hz, 1H), 1.43 (d, *J*= 7.2 Hz, 3H); ^{13}C NMR (400 MHz, DMSO-d6) δ 173.4, 162.4, 147.6, 144.3, 143.4, 143.3, 47.7, 17.1; LCMS-ESI (*m/z*): 196.0 $[M + H]^+$.

Synthesis of (*S*)-3-(4-hydroxyphenyl)-2-(pyrazine-2-carboxamido)propanoic acid (10n)

LiOH.H_2O (587 mg, 13.927 mmol, 2.5 eq.) was added to the stirred solution of methyl (pyrazine-2-carbonyl)-L-tyrosinate (**9n**) (1.6 g, 5.571 mmol, 1.0 eq.) in THF : H_2O (8:2, 20 mL) at 0 ^oC, then allowed to stir at rt for 1 h. The reaction mixture was concentrated under vacuum. To the residue water was added and pH was adjusted to 2-3 using 20% citric acid solution at 0 ^oC. The solid was filtered and washed with H_2O (5 mL), pet ether (10 mL× 3), dried under vacuum. The desired product (**10n**) as half white solid (78% yield); ^1H NMR (400 MHz, DMSO-d6) δ 12.94 (bs, 1H), 9.15 (d, *J*=1.6 Hz, 1H), 8.87 (d, *J*=2.4 Hz, 1H), 8.72 (m, 2H), 7.00 (d, *J*=8.4 Hz, 2H) 6.62 (d, *J*=8.4 Hz, 2H), 4.66 (m, 1H), 3.09 (m, 2H); ^{13}C NMR (400 MHz, DMSO-d6) δ 172.4, 162.4, 155.9, 147.8, 144.0, 143.4, 130.0, 127.2, 115.1, 53.6, 35.2; LCMS-ESI (*m/z*): 288.1$[M + H]^+$.

Synthesis of (*S*)-2-(pyrazine-2-carboxamido)-3-(pyridin-2-yl)propanoic acid (10o)

LiOH.H_2O (954 mg, 22.71 mmol, 2.5 eq.) was added to the stirred solution of methyl (*S*)-2-(pyrazine-2-carboxamido)-3-(pyridin-2-yl)propanoate (**9o**) (2.6 g, 9.055 mmol, 1 eq) in THF: H_2O (8:2, 30 mL) at 0 ^oC, then the reaction mixture was allowed to stir at same temperature

for 1 h. The reaction mixture was concentrated under vacuum. To the residue water was added and pH was adjusted to 2-3 using 20% citric acid solution at 0 ^{o}C. The aqueous layer was extracted with 10% MeOH/$CHCl_3$ (3× 100 mL) and the organic layer was separated, dried over Na_2SO_4 and evaporated under vacuum to get crude product, which was washed with ether (2× 25 mL) to yield the desired product (**10o**) as off white solid (57% yield); ^{1}H NMR (400 MHz, DMSO-d6) δ 9.13 (m, 2H), 8.86 (d, *J*=2.4 Hz, 1H), 8.72 (m, 1H), 8.48 (d, *J*=4.4 Hz, 1H), 7.66 (dt, *J*=1.6, 7.6 Hz, 1H), 7.27 (d, *J*=7.6, Hz, 1H), 7.20 (m, 1H), 4.94 (m, 1H), 3.35 (m, 2H); ^{13}C-NMR (400 MHz, DMSO-d6) δ 172.5, 162.7, 157.6, 149.1, 147.9, 144.2, 143.6, 136.8, 124.0, 122.0, 51.9, 38.2; LCMS-ESI (*m/z*): 273.2 $[M+H]^{+}$.

6. Synthesis of aldimines 13(a-g)

(*R*,*E*)-2-methyl-*N*-(3-methylbutylidene)propane-2-sulfinamide (13a)

A solution of isovaleraldehyde (**11a**) (10.8 mL, 99.01 mmol, 2.0 eq.) in dichloromethane (50 mL) was added drop wise to a stirred suspension of *R*-(+)-2-methyl-2-propane sulfinamide (**12**) (6.0 g, 49.50 mmol, 1.0 eq.), copper sulfate.5H_2O (61.8 g, 247.52 mmol, 5 eq.) and molecular sieves (4 Å, 60 g) in dichloromethane (250 mL) at room temperature. After stirring at room temperature for 48 h, the reaction mixture was filtered through celite and washed with dichloromethane (150 mL). The filtrate was then concentrated *in vacuo*. The resulting residue was purified by flash column chromatography on silica gel (230-400 mesh size) using petroleum ether/ethyl acetate (5-8%) as eluant. The desired aldimine (**13a**) was isolated as a colorless liquid (59% yield). ^{1}H NMR (400 MHz, $CDCl_3$) δ 8.05 (t, *J*= 5.2 Hz, 1H), 2.37 (m, 2H), 2.07 (m, 1H), 1.19 (s, 9H), 0.98 (d, *J*= 6.8 Hz, 6H). LCMS-ESI (*m/z*): 190.1 [M + H]+.

Following a procedure similar to that of **13a**, the following intermediates were synthesized.

(*R*,*E*)-*N*-hexylidene-2-methylpropane-2-sulfinamide (13b)

Yield: 62%. LCMS-ESI (*m/z*): 204.1[M + H]$^+$

(*R*,*E*)-*N*-(2-cyclohexylethylidene)-2-methylpropane-2-sulfinamide (13c)

Following same procedure of **13a** but stirred for 64 h at room temperature.

Yield: 11%; LCMS-ESI (*m/z*): 230.2 [M + H]$^+$.

(*R*,*E*)-*N*-(3-cyclohexylpropylidene)-2-methylpropane-2-sulfinamide (13d)

Yield: 53%; ^{1}H NMR (400 MHz, $CDCl_3$) δ 8.05 (t, *J*= 4.8 Hz, 1H), 2.52-2.49 (m, 2H), 2.33 (t, *J*= 7.6, Hz, 1H) 1.701.63 (m, 4H), 1.50-1.46 (m, 3H), 1.27-1.10 (m, 11H), 0.94-0.849 (m, 3H).

(*R*,*E*)-2-methyl-*N*-(2-phenylethylidene)propane-2-sulfinamide (13e)

Yield: 65%. LCMS-ESI (*m/z*): 224.1[M + H]$^+$

***(R*,*E*)-2-methyl-N-(3-phenylpropylidene)propane-2-sulfinamide (13f)**

Yield: 85%; ^{1}H NMR (400 MHz, $CDCl_3$) δ 8.11 (t, *J*= 4.4 Hz, 1H) 7.24 (m, 5H), 2.97 (m, 2H), 2.87 (m, 2H); LCMS-ESI (*m/z*): 238.1[M + H]$^+$.

(*R*,*E*)-*N*-(cyclohexylmethylene)-2-methylpropane-2-sulfinamide (13g)

Following same procedure of **13a** but stirred for 72 h at room temperature. Yield: 38%; LRMS-ESI (*m/z*): 216[M + H]$^+$.

(*R*)-2-methyl-N-((*R*)-3-methyl-1-(4,4,5,5-tetramethyl-1,3,2-dioxaborolan-2-yl)butyl)propane-2-sulfinamide (15a)

To a solution of tricyclohexylphosphine tetrafluoroborate (47 mg, 0.13 mmol, 0.012 eq.) in toluene (2.0 mL) were added aqueous copper sulfate solution (32 mg in 4.0 mL water, 0.13 mmol, 0.012 eq.) and benzylamine (0.06 mL, 0.529 mmol, 0.05 eq.) sequentially. The reaction mixture was stirred at room temperature for 10 min and then diluted with 12.0 mL of

toluene. To this mixture a solution of aldimine (**11a**) (2.0 g, 10.58 mmol, 1.0 eq.) in toluene (6 mL) and bispinacolatodiboron (**14**) (5.37 g, 21.164 mmol, 2.0 eq.) were added. Colour change was observed over a period of 10 min (blue to grey and then to a brown colour clear solution). The reaction mixture was stirred at room temperature for 20 h. The reaction mixture was diluted with ethyl acetate (20 mL) and filtered through deactivated silica gel [Silica gel: water (100: 20)]. The filtrate was concentrated *in vacuo*. The resulting residue was further purified by rapid column chromatography using deactivated silica gel eluting with (8 – 10%) ethyl acetate/petether to give low melting white solid of (**15a**) in 22% yield. ^{1}H NMR (400 MHz, $CDCl_3$) δ 3.11-3.06 (m, 2H), 1.76-1.45 (m, 3H), 1.25 (d, *J*= 5.6 Hz, 12H), 1.19 (s, 9H), 0.93 (d, *J*= 6.8 Hz, 6H); LCMS-ESI (*m/z*): 318.2 $[M + H]^+$.

Following a procedure similar to that of **15a**, the following intermediates were synthesized.

(*R*)-2-methyl-*N*-((*R*)-1-(4,4,5,5-tetramethyl-1,3,2-dioxaborolan-2-yl)hexyl)propane-2-sulfinamide (15b)

Yield: 30%; ^{1}H NMR (400 MHz, $CDCl_3$) δ 3.19 (d, *J*= 6.4 Hz, 1H), 3.02 (q, *J*= 6.4 Hz, 1H), 1.71-1.65 (m, 2H), 1.41-1.27 (m, 6H), 1.25 (d, *J*= 5.2 Hz, 12H), 1.19 (s, 9H), 0.86 (t, *J*= 6.8, 3H); LCMS-ESI (*m/z*): 332.1$[M + H]^+$

(*R*)-*N*-((*R*)-2-cyclohexyl-1-(4,4,5,5-tetramethyl-1,3,2-dioxaborolan-2-yl)ethyl)-2-methylpropane-2-sulfinamide (15c)

Yield: 28%; ^{1}H NMR (400 MHz, $CDCl_3$) δ 3.12-3.08 (m, 1H), 3.03 (d, *J*= 6.46z, 1H), 1.75-1.47 (m, 9H), 1.25-1.18 (m, 21H), 0.92-0.86 (m, 2H); ^{13}C NMR (101 MHz, $CDCl_3$) δ 83.4, 55.9, 40.9, 35.1, 33.4, 33.2, 26.5, 26.2, 24.9, 24.4, 22.5 LCMS-ESI (*m/z*): 358.2 $[M + H]^+$.

(*R*)-*N*-((*R*)-3-cyclohexyl-1-(4,4,5,5-tetramethyl-1,3,2-dioxaborolan-2-yl)propyl)-2-methylpropane-2-sulfinamide (15d)

Yield: 24%; ^{1}H NMR (400 MHz, $CDCl_3$) δ 3.14 (d, *J*= 6.4 Hz, 1H), 2.99 (q, *J*= 7.2 Hz, 1H), 1.69-1.60 (m, 9H), 1.34-1.12 (m, 25H), 0.90-0.85 (m, 3H); ^{13}C NMR (400 MHz, $CDCl_3$) δ

83.8, 55.8, 37.6, 34.3, 33.3, 33.1, 30.9, 26.6, 26.2, 24.9, 25.0, 24.8, 24.4, 22.5; LCMS-ESI (*m/z*): 372.3 $[M + H]^+$.

(*R*)-2-methyl-*N*-((*R*)-2-phenyl-1-(4,4,5,5-tetramethyl-1,3,2-dioxaborolan-2-yl)ethyl)propane-2-sulfinamide (15e)

Yield: 36%; ^{1}H NMR (400 MHz, $CDCl_3$) δ 7.27-7.14 (m, 5H), 3.34 (q, *J*= 6.8 Hz, 1H), 3.20 (d, *J*= 6.8 Hz, 1H), 3.0 (dd, *J*= 6.4, 1.6 Hz, 2H), 1.23-1.17 (m, 21H); LCMS-ESI (*m/z*): 352.2$[M + H]^+$

(*R*)-2-methyl-*N*-((*R*)-3-phenyl-1-(4,4,5,5-tetramethyl-1,3,2-dioxaborolan-2-yl)propyl)propane-2-sulfinamide (15f)

Yield: 30%; ^{1}H NMR (400 MHz, $CDCl_3$) δ 7.21 (m, 5H), 3.29 (d, *J*= 6.8 Hz, 1H), 3.09 (q, *J*= 6.8 Hz, 1H), 2.70 (m, 2H), 2.00 (m, 2H), 1.26 (d, *J*= 2.8 Hz, 12H), 1.23 (s, 3H), 1.19 (s, 6H); ^{13}C NMR (101 MHz, $CDCl_3$) δ 141.8, 128.4 (2C), 128.3 (2C), 125.8, 84.1 (2C), 56.0, 35.3, 33.1, 24.9, 24.8 (4C), 24.5 (3C), 22.5; LCMS-ESI (*m/z*): 366.2 $[M + H]^+$.

(*R*)-*N*-((*R*)-cyclohexyl(4,4,5,5-tetramethyl-1,3,2-dioxaborolan-2-yl)methyl)-2-methylpropane-2-sulfinamide (15g)

Under nitrogen, a solution of freshly prepared (ICy)CuO*t*Bu (60 mg, 0.18 mmol, 0.1 equiv) in dry toluene (1.0 mL) was added to the stirred mixture of bis(pinacolato)diboron (**14**) (665 mg, 2.65 mmol, 1.5 equiv) and hexyl sulfinyl imine (**13g**) (380 mg, 1.77 mmol, 1.0 equiv) in dry toluene (5 mL) at room temperature. The stirring was continued for 14 h at room temperature. The reaction mixture was diluted with ethyl acetate (20 mL) and filtered through deactivated silica gel [Silica gel: water (100: 20)]. The filtrate was concentrated *in vacuo*. The resulting residue was further purified by rapid column chromatography using deactivated silica gel [Silica gel: water (100: 20)] eluting with 8 –10% ethyl acetate/hexane to give to yield the desired product (**15g**) as a white solid (33% yield).^{1}H NMR (400 MHz, $CDCl_3$) δ

3.33 (d, J = 7.0 Hz, 1H), 2.94 – 2.83 (m, 1H), 1.61 (s, 8H), 1.27 (s, 6H), 1.25 (s, 6H), 1.24 (s, 3H), 1.19 (s, 8H). LCMS-ESI (*m/z*): 344.0 $[M + H]^+$.

7. Synthesis of amino boronate salts 16(a-g)

(*R*)-3-methyl-1-(4,4,5,5-tetramethyl-1,3,2-dioxaborolan-2-yl)butan-1-amine (16a)

Under nitrogen, dry methanol (0.9 mL, 22.08 mmol, 10.0 equiv) was added to a solution of boronate (**15a**) (700 mg, 2.21 mmol, 1.0 eq) in dry 1,4-dioxane (10 mL) at room temperature. Then 4.0 M HCl [0.7 mL, 2.65 mmol, 1.2 equiv (solution in 1,4-dioxane)] was added and the resulting mixture was stirred at room temperature for 2 h. The solvent was removed under reduced pressure and the resulting solid was triturated with 3:1 mixture of hexanes:Et_2O to obtain the desired product (**16a**) (61%) as a white solid.

^1H NMR (400 MHz, $CDCl_3$) δ 8.17 (s, 1H), 2.93 (br s, 1H), 1.90 (dt, J = 13.3, 6.6 Hz, 1H), 1.77 (dt, J = 14.9, 7.6 Hz, 1H), 1.68 – 1.56 (m, 1H), 1.28 (s, 12H), 0.94 (d, J = 6.5 Hz, 6H). ^{13}C NMR (101 MHz, $CDCl_3$) δ 85.0, 38.5, 36.0 (br), 25.1, 24.9, 24.6, 22.5, 22.4. LRMS-ESI (*m/z*): 214.0$[M + H]^+$

Following a procedure similar to that of **16a**, the following intermediates were synthesized.

(*R*)-1-(4,4,5,5-tetramethyl-1,3,2-dioxaborolan-2-yl)hexan-1-amine (16b)

Yield: 58%. LRMS-ESI (*m/z*): 228.2$[M + H]^+$

(*R*)-2-cyclohexyl-1-(4,4,5,5-tetramethyl-1,3,2-dioxaborolan-2-yl)ethanamine (16c)

Yield: 55%. LRMS-ESI (*m/z*): 253.0$[M + H]^+$

(*R*)-3-cyclohexyl-1-(4,4,5,5-tetramethyl-1,3,2-dioxaborolan-2-yl)propan-1-amine (16d)

Yield: 83%. LRMS-ESI (*m/z*): 268.2$[M + H]^+$

(*R*)-2-phenyl-1-(4,4,5,5-tetramethyl-1,3,2-dioxaborolan-2-yl)ethanamine (16e)

Yield: 71%. LRMS-ESI (*m/z*): 248.0$[M + H]^+$

(*R*)-3-phenyl-1-(4,4,5,5-tetramethyl-1,3,2-dioxaborolan-2-yl)propan-1-amine (16f)

Yield: 63%. LRMS-ESI (*m/z*): (*m/z*): 262.0[M + H]$^+$

(*R*)**-cyclohexyl(4,4,5,5-tetramethyl-1,3,2-dioxaborolan-2-yl)methanamine (16g)**

Yield: 80%. LRMS-ESI (*m/z*): 240.0[M + H]$^+$

8. Synthesis of diamides (18a-ff/19/21) and compound 22

General procedure for the preparation of diamides (18a-ff/19/21)

Under nitrogen, a solution of *i*Pr_2NEt (5.0 eq) in dry dichloromethane was added drop wise to a suspension of ammonium salt 1**6a-g** (1.0 eq), acids (benzoic acid/picolinic acid/nicotinic acid/***N***-Boc-*L*-phenylalanine/3-phenylpropanoic acid**/6/10a-o) (**1.2 eq), and TBTU (1.0 eq) in dry dichloromethane (0.2 molar) at 0 ^{o}C. The mixture was slowly warm to room temperature and stirred for 16 h. The reaction mixture was diluted with ethyl acetate. The organic layer was washed with 3% aqueous potassium carbonate, 3% aqueous citric acid water and brine. The organic phase was dried over sodium sulphate and the solvent was removed under vacuum. The residues **18a-ff/19/21** were used without further purification.

(*S*)-2-amino-N-((*R*)-3-methyl-1-(4,4,5,5-tetramethyl-1,3,2-dioxaborolan-2-yl)butyl)-3-phenylpropanamide hydrochloride (22)

Under nitrogen, 4.0 M HCl (as a solution in 1,4-dioxane, 0.8 mL, 3.19 mmol, 3.5 equiv) was added drop wise to a solution of *N*-Boc-boronate **21** (420 mg, 0.91 mmol, 1.0 equiv) in dry CH_2Cl_2 (5.0 mL) at 0 ^{o}C. The reaction mixture was slowly warmed to room temperature and stirred for 12 h. The mixture was concentrated under reduced pressure and the resulting solid was washed with hexane and dried. The crude solid **22** was carried on to the next step without purification. LRMS-ESI (*m/z*): 361.3 [M + H]$^+$.

9. NMR spectra of compound 58

^{1}H NMR (400 MHz) of compound **58** in CD_3OD

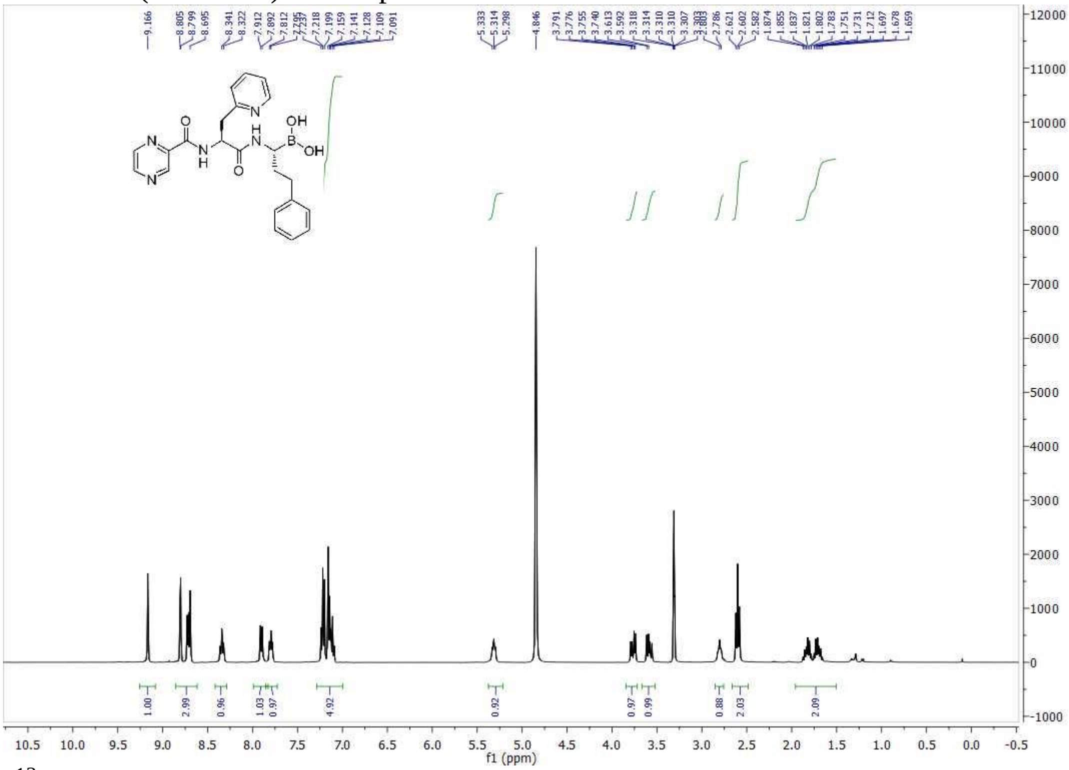

^{13}C NMR (101 MHz) of compound **58** in CD_3OD

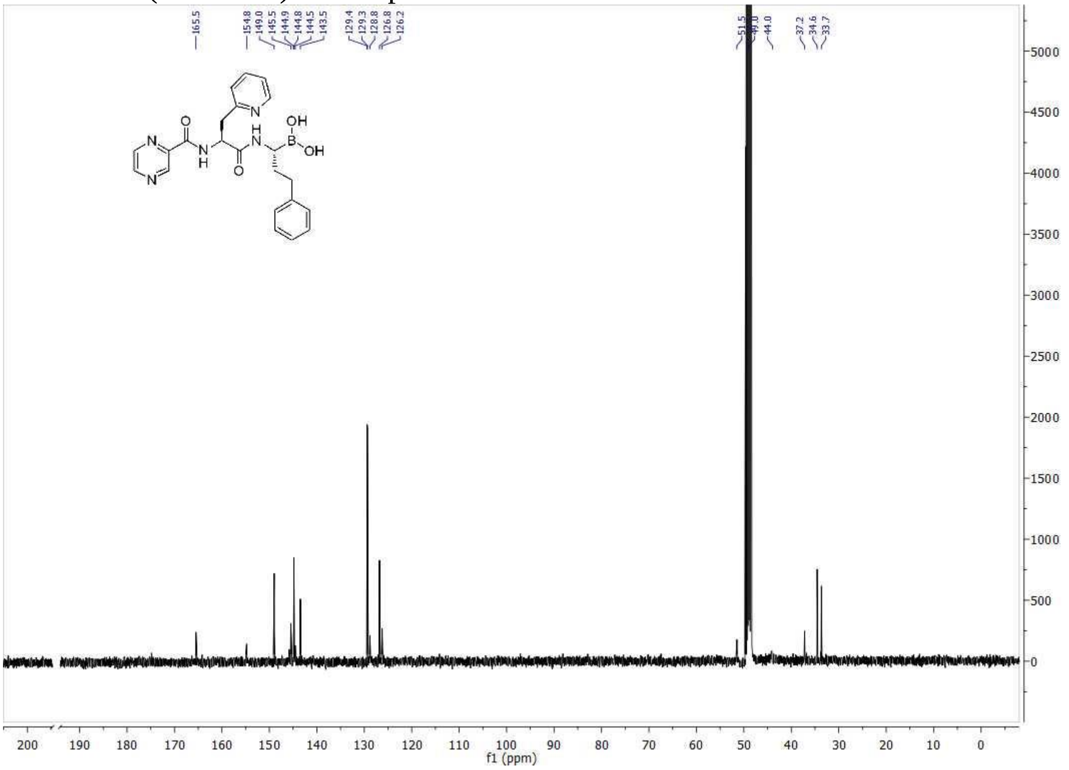

HMQC of compound **58** in CD_3OD

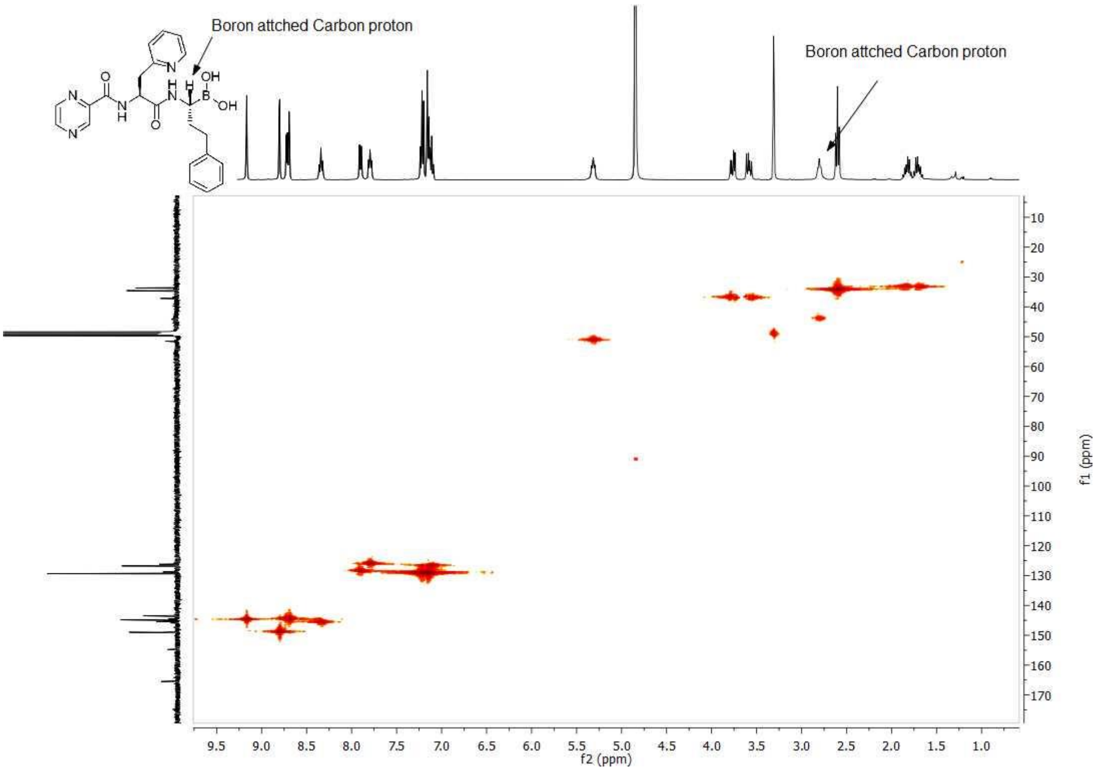

10. Protease Panel Testing of compound 58

Target	Super-Family	IC_{50} (μM)	Control Cpd IC_{50} (μM)	Control Cpd ID
ACE1	metallopeptidase		3.79E-08	Captopril
ACE2	metallopeptidase		1.35E-09	ACE 2 inhibitor
ADAM10	metallopeptidase		8.11E-08	GM6001
BACE1	aspartic peptidase		2.66E-07	B-Secretase inhibitor IV
Calpain 1	cysteine peptidase		9.80E-08	E64
Caspase 1	cysteine peptidase		5.45E-08	IETD-CHO
Caspase 2	cysteine peptidase		6.26E-07	IETD-CHO
Caspase 3	cysteine peptidase		9.13E-10	DEVD-CHO
Caspase 4	cysteine peptidase		2.73E-06	IETD-CHO
Caspase 5	cysteine peptidase		2.13E-08	IETD-CHO
Caspase 6	cysteine peptidase		9.04E-09	DEVD-CHO
Caspase 7	cysteine peptidase		1.10E-09	DEVD-CHO
Caspase 8	cysteine peptidase		1.66E-09	IETD-CHO
Caspase 9	cysteine peptidase		5.53E-08	IETD-CHO
Caspase 10	cysteine peptidase		1.77E-08	IETD-CHO
Caspase 11	cysteine peptidase		6.80E-07	IETD-CHO
Cathepsin B	cysteine peptidase		9.87E-09	E64
Cathepsin C	cysteine peptidase		6.07E-07	E64
Cathepsin G	cysteine peptidase		3.83E-06	Chymostatin
Cathepsin H	cysteine peptidase		1.98E-07	E64
Cathepsin L	cysteine peptidase		9.99E-09	E64
Cathepsin S	cysteine peptidase		9.39E-09	E64
Cathepsin V	cysteine peptidase		1.18E-08	E64
**Chymase**	**serine peptidase**	**5.75E-06**	**9.15E-08**	**Chymostatin**
**Chymotrypsin**	**serine peptidase**	**1.22E-07**	**1.30E-09**	**Chymostatin**
DPP IV	serine peptidase		1.18E-07	P32/98
DPP VIII	serine peptidase		9.40E-07	P32/98
DPP IX	serine peptidase		2.21E-06	P32/98
Elastase	serine peptidase		1.81E-06	Gabexate mesylate (GM)
FVIIa	serine peptidase			Gabexate mesylate (GM)
FXa	serine peptidase		5.30E-06	Gabexate mesylate (GM)
FXIa	serine peptidase		5.07E-07	Gabexate mesylate (GM)
HIV-1	aspartic peptidase		6.99E-08	Pepstatin A
**Kallikrein 1**	**serine peptidase**	**6.03E-06**	**2.67E-05**	**Leupeptin**
Kallikrein 5	serine peptidase		8.06E-06	Gabexate mesylate (GM)
**Kallikrein 7**	**serine peptidase**	**9.31E-06**	**2.93E-05**	**Gabexate mesylate (GM)**
Kallikrein 12	serine peptidase		3.51E-07	Gabexate mesylate (GM)
Kallikrein 13	serine peptidase		1.76E-05	Gabexate mesylate (GM)
Kallikrein 14	serine peptidase		9.69E-07	Gabexate mesylate (GM)
Matriptase 2	serine peptidase		7.56E-06	Gabexate mesylate (GM)
MMP 1	metallopeptidase		7.41E-10	GM6001
MMP 2	metallopeptidase		7.12E-10	GM6001

Target	Super-Family	IC_{50} (μM)	Control Cpd IC_{50} (μM)	Control Cpd ID
MMP 3	metallopeptidase		1.63E-08	GM6001
MMP 7	metallopeptidase		4.38E-09	GM6001
MMP 8	metallopeptidase		5.88E-10	GM6001
MMP 9	metallopeptidase		7.77E-10	GM6001
MMP 10	metallopeptidase		7.54E-09	GM6001
MMP 12	metallopeptidase		1.17E-09	GM6001
MMP 13	metallopeptidase		5.35E-10	GM6001
MMP 14	metallopeptidase		1.24E-09	GM6001
Neprilysin	metallopeptidase		4.22E-08	Phosphoramidon
Papain	cysteine peptidase		9.36E-10	E64
Plasma Kallikrein	serine peptidase	4.40E-06	4.75E-07	Gabexate mesylate (GM)
Plasmin	serine peptidase		9.36E-07	Gabexate mesylate (GM)
Proteinase A	serine peptidase	1.94E-06	9.86E-05	Leupeptin
Proteinase K	serine peptidase	3.52E-07	1.91E-07	Proteinase K inhibitor
TACE	metallopeptidase		9.10E-09	GM6001
Thrombin a	serine peptidase		3.73E-06	Gabexate mesylate (GM)
Trypsin			6.69E-08	Gabexate mesylate (GM)
Tryptase b2			8.73E-08	Gabexate mesylate (GM)
Tryptase g1			5.06E-08	Gabexate mesylate (GM)
Urokinase			1.00E-07	Gabexate mesylate (GM)
